# In vitro reconstitution of chromatin replication recapitulates symmetric histone recycling

**DOI:** 10.1038/s41467-026-76520-5

**Published:** 2026-08-11

**Authors:** Fritz Nagae, Shizuko Endo, Yasuto Murayama, Tsuyoshi Terakawa

**Affiliations:** 1 Department of Biophysics, Graduate School of Science, Kyoto University, Kyoto, Japan; 2 Department of Chromosome Science, National Institute of Genetics, Shizuoka, Japan; 3 Department of Genetics, Graduate University for Advanced Studies (SOKENDAI), Shizuoka, Japan; 4 Present Address: Department of Bioscience, Technical University of Munich, Garching, Germany

**Keywords:** Nucleosomes, Replisome

## Abstract

Symmetric histone recycling is vital for maintaining epigenetic inheritance upon eukaryotic DNA replication. Recent genome-wide studies have uncovered key determinants of this process, but how these factors collectively support parental histone transfer remains incompletely understood. Here, we successfully reconstitute histone recycling with 24 purified proteins and analyze the products digested by Micrococcal nuclease with Repli-pore-seq, the newly developed pipeline combining nanopore sequencing and deep-learning-based classification. As a result, we identify histones symmetrically recycled as tetrasomes or hexasomes on nucleosome-favorable sequences. We also observe the discordance of the recycled position between lagging and leading strands on the GC-rich DNA sequences. Moreover, removal of Pol δ, Pol32, Dpb3/4, Ctf4, Csm3/Tof1, or Mrc1 disrupts the balance of histone recycling between the two daughter strands, whereas removal of Ctf4, Csm3/Tof1, or Mrc1 additionally alters the positions at which histones were recycled. Furthermore, addition of the lagging-strand maturation factors Fen1 and Cdc9 enhances histone recycling to the lagging strand. These findings provide critical insights into the molecular players and mechanisms underlying symmetric histone recycling.

## Abstract (web summary)

Symmetric recycling of parental histones to newly replicated DNA is vital for maintaining cell identity. Repli pore-seq reveals the reconstitution of this process from purified proteins, providing an in vitro system for studying parental histone recycling.

## Introdution

Epigenetic information, which is encoded in chromatin, is crucial to regulate transcriptional patterns and to determine functions and fates of cells^1–8^. Faithful inheritance of epigenetic information across generations is essential for maintaining cellular identities. The nucleosome, a basic unit of chromatin, is a protein/DNA complex in which 147 base-pairs (bp) of DNA wraps around a histone octamer composed of one H3/H4 tetramer and two H2A/H2B dimers^9^. Post-translational modifications (PTMs) in histones are one representative carrier of epigenetic information to regulate gene expressions^4,6,10^. Recycling modified histones from parental chromatin to either of two replicated DNA strands is the first step of epigenetic inheritance, reportedly coupled with DNA replication^1–8,11^. Symmetric recycling of parental histones to the replicated strands is vital for maintaining cellular identity after cell division^12–15^.

Deep-sequencing-based studies using eSPAN^16,17^ and SCAR-seq^18,19^ have demonstrated that parental histones are symmetrically recycled to the lagging and leading strands at a replication fork. The techniques have identified protein regions to regulate symmetric histone recycling^17,19–31^. Mcm2, a subunit of a replicative helicase, has a histone-binding domain on its N-terminal tail and contributes to recycling to the lagging strand^19^. Additionally, other subunits of replisome (Ctf4^20^, Pol α^17,20,21^, Pol δ^21,22^, PCNA^22^, and Tof1^23^) are proposed to participate in the recycling to the lagging strand. On the other hand, Dpb3 and Dpb4, non-essential subunits of Pol ε, are essential for recycling to the leading strand^24^. Mrc1, one subunit of the replisome, is involved in histone recycling to both lagging and leading strands^25–27^. A histone chaperone FACT, captures parental histones ahead of a replication fork and contributes to recycling to both strands^28,29^. A deep-sequencing study for cells with combinatorial mutations in Mcm2, Dpb3/4, and Pob3 (a subunit of FACT) demonstrated that defects of histone recycling to daughter strands are additive^29–31^, indicating the coexistence of multiple histone-recycling pathways. Together, these cell-based experiments suggested that the symmetry of histone recycling relies on a balance among the recycling pathways.

Although these genome-wide sequencing studies have revealed symmetric histone recycling, how the replisome itself contributes to the symmetry has been elusive. In cellular environments, coexisting enzymes, such as transcription, repair, and nucleosome remodeling machinery potentially modulate replication progression and nucleosome formation and positioning^32–35^. Moreover, epigenetic modification enzymes restore histone modification patterns by introducing certain PTMs, including those commonly used as markers of parental histones, onto newly synthesized histones during chromatin replication^6,10^. Because this restoration process is tightly coupled to parental histone recycling at the replication fork in vivo, it is difficult to distinguish the direct contribution of histone recycling itself from downstream chromatin restoration processes and to mechanistically dissect the role of individual replication factors in epigenetic inheritance. Furthermore, combinatorial protein depletion disrupted cell growth and downstream analyses, making it challenging to identify the minimal protein list for symmetric histone recycling. Therefore, in vitro reconstitution of chromatin replication, which allows adding or removing proteins from the system and isolating the contribution to histone recycling, is highly desirable.

In vitro reconstitution of biological systems is a powerful tool for understanding the fundamental mechanisms of complex cellular processes. The reconstitution of eukaryotic DNA replication identified the minimal protein list of DNA replication and molecular mechanisms of the process^36–38^. In addition, a combination of the in vitro DNA replication and other reconstitutions (*e.g*., nucleosome^39–44^, R-loop^45^, or cohesin^46^) has successfully revealed the coordination of eukaryotic DNA replications and the other processes. These studies demonstrated that the reconstituted replisome can recycle histones onto the replicated DNA strands in the absence or presence of histone chaperones^39,47^. However, the gel electrophoresis-based assays used in the studies did not provide critical information on the recycling ratios of the lagging and leading strands and the recycling positions, which has hindered elucidation of the molecular players and mechanisms underlying symmetric histone recycling.

Here, we reconstituted histone recycling with 24 purified proteins and analyzed the products digested by Micrococcal nuclease with the newly developed pipeline called Repli-pore-seq, in which nanopore sequencing and deep-learning-based classification were combined. The results confirmed that histones are symmetrically recycled to the lagging and leading strands, forming tetrasomes or hexasomes on DNA sequences energetically favorable for nucleosome formation. Interestingly, we identified positional discordance of recycled histones between the lagging and leading strands on GC-rich DNA regions, though they have the same DNA sequence. Furthermore, removal of Pol δ, Ctf4, Csm3/Tof1, Mrc1, Pol32, and Dpb3/4 disrupted histone recycling symmetry in a manner consistent with previous in vivo studies^20–22,25,48^. In addition, removal of Ctf4, Csm3/Tof1, or Mrc1 altered the positions at which histones were recycled. Finally, the addition of the lagging-strand maturation factors Fen1 and Cdc9 increased recycling to the lagging strand, suggesting that lagging-strand maturation modulates histone recycling symmetry. These findings shed light on the molecular components and mechanisms that drive symmetric histone recycling, paving the way for a deeper understanding of epigenetic transmission.

## Results

### In vitro reconstitution of chromatin replication-coupled histone recycling

**Fig. 1:**
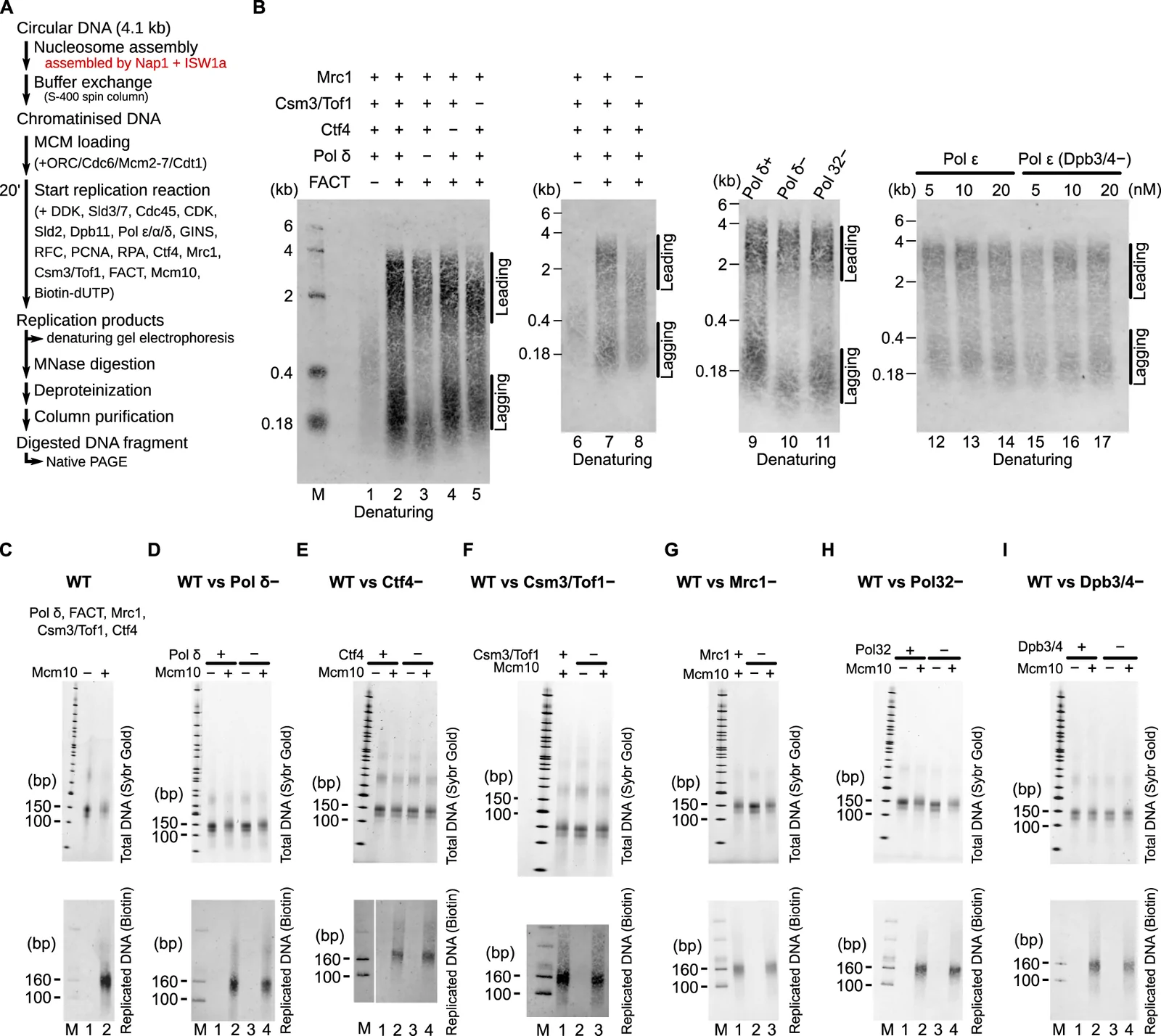
In vitro reconstitutions of chromatin replication. **A** Schematic of the in vitro DNA replication assay using plasmid DNA assembled into nucleosomes. **B** Alkaline denaturing agarose gel showing replicated DNA prior to MNase digestion. ‘M’ denotes a marker (See the Methods). The experiment was repeated three times giving consistent results. **C–I** Native polyacrylamide gel analysis of total DNA (top) and replicated DNA (bottom) after MNase digestion. Replication reactions in (**C**) were performed according to the protocol shown in (**A**). Reactions in (**D–G**) were carried out with Pol δ, Ctf4, Csm3/Tof1, or Mrc1 omitted during the firing step. Reactions in (**H**) and (**I**) were performed using mutant Pol δ and Pol ε lacking the non-catalytic subunits Pol32 and Dpb3/4, respectively. ‘M’ denotes a marker (See the Methods). The experiment was repeated twice giving consistent results. Source data are provided as a Source Data file.

We mixed purified proteins for *Saccharomyces cerevisiae* DNA replication and histone chaperone FACT with chromatinized DNA substrates to reconstitute in vitro chromatin replication, as described previously^38,39,43^. After incubation of the 4.1-kbp plasmid DNA substrate harboring an ARS consensus sequence (ACS) site with histones, Nap1, and ISW1a, chromatinized DNA was recovered by gel-filtration (Fig. 1A and Supplementary Fig. 1A, lane 5). In contrast, when an identical mixture lacking DNA was subjected to the same gel-filtration procedure, histones were scarcely detected in the eluate (Supplementary Fig. 1A, lane 7). These results indicate that the gel-filtration step efficiently removes free histones, while histones incorporated into DNA-bound nucleosomes are retained and recovered with the DNA. We added ORC, Cdc6, and Mcm2-7/Cdt1 to assemble MCM double hexamer on the nucleosome substrate (Fig. 1A). Following MCM activation by DDK, we initiated DNA replication by adding firing factors (Sld2, Sld3/7, Dpb11, Cdc45, GINS, Csm3/Tof1, Ctf4, Mrc1, RFC, PCNA, RPA, Pol α, Pol δ, Pol ε, and Mcm10), S-CDK and histone chaperone FACT in the reaction (Fig. 1A). Newly replicated DNA was labeled by incorporation of biotin-dUTP in the reaction mixture (Fig. 1A). Denaturing gel analysis confirmed the occurrence of replisome-dependent DNA replication, which was charactered by the productions of continuous leading (~2 kb) and discontinuous lagging (~0.2 kb) strands (Fig. 1B lane 2 & Supplementary Fig. 1B). The result showed that FACT was indispensable for replicating chromatin (Fig. 1B lane 1 & Supplementary Fig. 1B), consistent with the previous biochemical experiments^39,46^.

Previous studies have demonstrated that nucleosome assembly by salt gradient dialysis (SGD) results in irregularly spaced nucleosome arrays, while Nap1 and ISW1a (NI) facilitate evenly spaced nucleosome assembly^41,49^. In addition, ISW1a has been shown to promote phased arrays when ORC binds to the replication initiation site (ACS)^40^. To assess whether chromatin assembled by SGD or by NI provides a suitable substrate for in vitro chromatin replication, we assembled nucleosomes on the plasmid DNA using SGD and analyzed the resulting replication products (Supplementary Fig. 1C). Replication proceeded on SGD-assembled chromatin when histones were mixed with plasmid DNA at an equimolar ratio, although nucleosome assembly reduced overall replication efficiency compared with naked DNA (Supplementary Fig. 1C, lanes 1, 2, and 7). Furthermore, little or no signal from the replicated DNA strand was detected when the histone amount was increased to twice that of the plasmid DNA (Supplementary Fig. 1C, lanes 3–6). In contrast, replication reactions on NI-chromatin successfully produced both leading and lagging strands, despite being reconstituted with histones at a 100-fold excess over DNA. These results indicated that SGD-chromatin is an unsuitable substrate for efficient chromatin replication rather than NI-chromatin, consistent with the previous study^40^. Therefore, we used the NI-chromatin for all the experiments hereafter.

To test nucleosome formation after DNA replication, we treated the products with MNase to digest naked DNA regions (Fig. 1A). After separating the digested product by native polyacrylamide gel electrophoresis and subsequent gel stain with SYBR^TM^ Gold for detecting all DNA species, we observed ~150 bp DNA fragments in the reaction with and without Mcm10, replication initiation factor^50,51^ (Fig. 1C, lanes 1 of the top panel). When detecting replicated (biotinylated) DNA, a ~ 150 bp DNA band was only seen in the reaction containing Mcm10 (Fig. 1C, lanes 2 of the bottom panel). No detectable signal was observed either in chromatin-containing reactions lacking Mcm10 (Fig. 1C, bottom panel, lanes 1) or in replication reactions using naked DNA substrates (Supplementary Fig. 1D), ruling out the presence of ~150 bp biotinylated DNA fragments independent of chromatin replication. Furthermore, to examine whether Nap1 can mediate replication-independent histone deposition, we prepared a control fraction by mixing Nap1, ISW1a, and histones at the same concentrations used for nucleosome assembly, but without naked DNA substrates, and then performing gel filtration. Replication reactions performed with naked DNA substrates in this fraction produced only a barely detectable ~150 bp biotinylated DNA band (Supplementary Fig. 1E and 1F, lanes 3 and 6), indicating that Nap1- and ISW1a-mediated nucleosome assembly in the absence of chromatin replication is negligible. These data support that the purified proteins listed in Fig. 1A are sufficient for successful recycling of histones onto newly replicated DNA, consistent with the previous results^39,47^.

Next, we omitted proteins demonstrated to be involved in histone recycling in living cells^20–23^ from the current reconstituted chromatin replication. Previous studies have demonstrated that Pol δ, Ctf4, and Csm3/Tof1 are dispensable for the reconstituted system with naked DNA while omission of Mrc1 slows down replication reaction^38,43,52^. Pol δ is responsible for the lagging-strand synthesis^53,54^, which Pol α can replace. Ctf4, Csm3/Tof1, and Mrc1 are at the front edge of the replisome and work as scaffolds for other replication proteins (e.g., Pol α and FACT)^20,23,25–27,42,55,56^. In addition, Pol32 and Dpb3/4 which are histone-binding subunits of Pol δ and ε, respectively, are dispensable for DNA synthesis reaction^57,58^. We repeated the replication assays without Pol δ, Ctf4, Csm3/Tof1, Mrc1, Pol32, or Dpb3/4 (Pol δ − , Ctf4 − , Csm3/Tof1 − , Mrc1 − , Pol32 − , or Dpb3/4 − ). Dpb3/4 and Pol32 are protein subunits but are listed alongside other replication proteins for simplicity. The replicated products in the Ctf4 − , Csm3/Tof1 − , Mrc1 − , Pol32 − , and Dpb3/4− reactions were similar in their presence (Fig. 1B, lanes 2 vs 4 and 5, lanes 7 vs 8, and lanes 9 vs 11, and lanes 14 vs 17 & Supplementary Fig. 1B). On the other hand, removing Pol δ reduced the length and amount of the lagging strand while effects on the leading strand were moderate (Fig.1B lane 2 vs 3 & Supplementary Fig. 1B), consistent with the results using naked DNA as a substrate^38^.

Inspection of MNase-digested replicated (biotinylated) DNA revealed clear nucleosome-protected fragments after replication both in the presence and absence of Pol δ, Ctf4, Csm3/Tof1, Mrc1, Pol32, or Dpb3/4 (Fig. 1D–I). As a representative example, analysis of the bottom panel of Fig. 1D shows that clear MNase-protected bands are detected on replicated DNA both in the presence and absence of Pol δ (lanes 2 vs 4), whereas little to no protected signal is observed prior to replication under either condition (lanes 1 vs 3). This indicates that nucleosomes are assembled on daughter DNA regardless of the presence of Pol δ. Consistent with this observation, MNase-protected fragments were detected after replication in the absence of other replisome components, including Ctf4, Csm3/Tof1, Mrc1, Pol32, and Dpb3/4 (Fig.1E–I). Although substantial nucleosome deposition on replicated DNA was still observed, omission of Pol32 or Dpb3/Dpb4 moderately reduced the levels of newly synthesized nucleosomal fragments compared with reactions containing these factors (Supplementary Fig. 1G). These results suggest that Pol32 and Dpb3/Dpb4 contribute to efficient histone recycling, while other factors may partially support parental histone transfer in their absence. Together, these results indicated that Pol δ, Ctf4, Csm3/Tof1, Mrc1, Pol32, and Dpb3/4 are dispensable for obtaining nucleosome footprints on replicated DNA for histone deposition to replicated DNA.

### Deep learning model to detect biotinylated DNA with nanopore sequencing

**Fig. 2:**
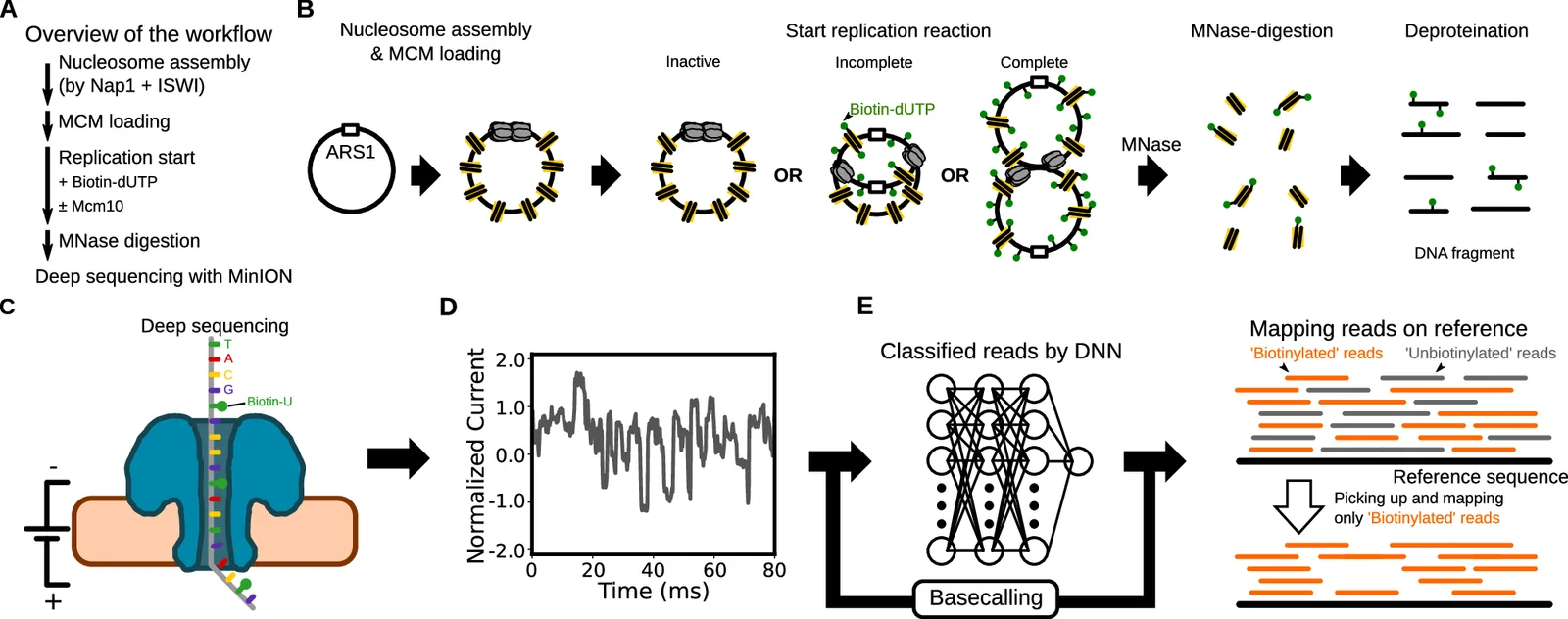
The Repli-pore-seq pipeline. **A** The protocol of the pipeline. **B** Schematic of In vitro chromatin reSchematic of the deep-neural network model. The model is composed of residual blocks with convolution and LSTM layers. The values in the arrays obtained in (**D**) were converted into a scholar within the range [0, 1] and were fed into the network. The reads were labeled as ‘biotinylated’ if the output was ≥0.5; otherwise, they were labeled as ‘unbiotinylated’.

To analyze the recycling ratio to the lagging and leading strands and recycling position, we established a pipeline called Repli-pore-seq by combining nanopore sequencing and deep-learning-based classification. In this pipeline, we first performed in vitro chromatin replication assays (Figs. 1A, 2A, B). After the MNase digestion, DNA fragments are a mixture of template unmodified DNA and replicated biotinylated DNA since all substrates could not consistently be replicated (Fig. 2B). To computationally rather than biochemically “purify” the replicated DNA, we performed nanopore sequencing of the mixture with the MinION device (Oxford Nanopore Technologies) and classified the template unmodified DNA and the replicated biotinylated DNA based on their electric current signals (Fig. 2A–E). In nanopore sequencing, one single-stranded DNA strand passes through a nanopore embedded in the membrane, and its sequence is identified based on changes in the current signal through occluding the pore by the nucleotides (Fig. 2C, D). To visualize how incorporation of biotin-dUTP perturbs nanopore current signals, we aligned raw current traces to the reference DNA sequence using the Adaptive Banded Event Alignment (ABEA) algorithm^59^ (Supplementary Fig. 2A, B). This analysis revealed that biotinylated nucleotides exhibit characteristic current patterns that are distinct from those of the four canonical nucleotides, consistent with previous reports^60,61^. These results support the feasibility of classifying biotinylated DNA based on nanopore signal features. Although biotin-dUTP incorporation modulated nanopore current signals and reduced basecalling quality scores (Q-scores) (Supplementary Fig. 2C), the fraction of reads successfully aligned to the reference sequence was comparable for unbiotinylated and biotinylated DNA (93.3% and 91.2%, respectively). Together, we developed a deep-learning-based classifier to detect the current signals unique to the incorporated biotin-dUTP.

We combined the residual neural network with Long Short-Term Memory (LSTM) to establish the classifier (Fig. 2E and Supplementary Fig. 2D, E; See Methods for details). For training, we used biotinylated and unbiotinylated DNA substrates, which were generated by PCR and measured the current signals of each substrate using the MinION device. Importantly, classification of biotinylated reads is performed solely on raw nanopore current signals, whereas base-called sequence information is used after classification for sequence mapping and downstream positional analyses. Because both the training data and the experimental Repli-pore-seq data derive from the same defined DNA sequence, splitting the dataset by sequence properties (such as GC content or strand orientation) would artificially segregate specific sequence contexts between the training and validation sets. We therefore adopted random splitting to ensure equivalent representation of sequence features across both datasets. Consistent with this design, AT content and strand orientation were nearly identical between the training and validation sets (53.2 ± 3.1% AT content and 46.7% vs. 46.9% Watson-strand reads), indicating that classification performance is not driven by sequence-specific bias. Also, because the same DNA sequence was used for both the replication assays and classifier training, and all proteins were removed prior to library preparation, the classification relies solely on biotin-dUTP incorporation into DNA fragments. The current signal trajectories were segmented according to their stepwise change, and each segment’s mean, standard deviation, and duration were calculated (Fig. 2D). Each series of the segments from unbiotinylated and biotinylated DNA was labeled as ‘0’ and ‘1’ respectively, and an equal number of the signals from each class were mixed to create the training data. We trained the classifier to predict whether each read contains biotin-dUTP–incorporated nucleotides, as illustrated by the training and validation loss curves in Supplementary Fig. 2G.

We evaluated the performance of the trained classifier. Predicted labels of unbiotinylated and biotinylated DNA substrates for the validation dataset were distributed around 0 and 1, respectively (Supplementary Fig. 2F). Few instances in both substrates showed the predicted labels around 0.5. Therefore, we considered the label below 0.5 as ‘unbiotinylated’ and the others as ‘biotinylated.’ A confusion matrix showed that the accuracy and precision of the model were 98% and 99%, respectively (Supplementary Fig. 2F inset). The accuracies of simpler classification models, such as logistic regression and random forests, were 65% and 67%, respectively, indicating that the classification task in the present study requires the expressive power of a deep neural network.

### Repli-pore-seq confirmed reconstituted symmetric histone recycling

**Fig. 3:**
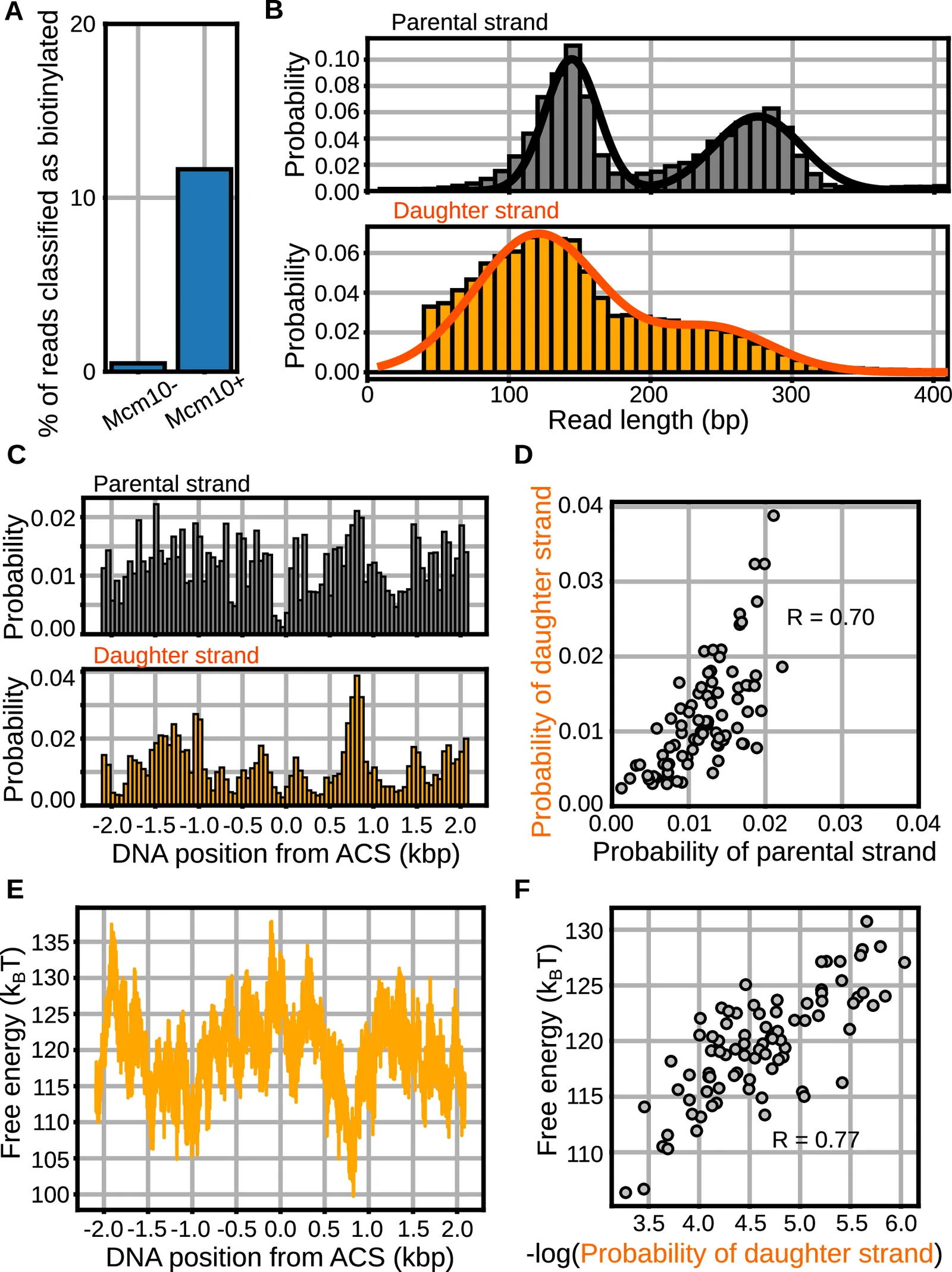
Repli-pore-seq of the reconstituted chromatin replication. **A** Fractions of biotinylated reads in the Mcm10− and Mcm10+ reactions. **B** The read length distributions of parental DNA fragments (top, gray) and daughter DNA fragments (bottom, orange). The distributions were fitted using two Gaussians. **C** The position distributions of histones on the parental DNA (top; gray) and the daughter DNA (bottom; orange). **D** The correlation plot for the position distributions of histones on the parental and daughter DNA in (**C**). **E** The theoretically calculated energy landscape of nucleosome formation along the template DNA. **F** The correlation plot for the energy landscape in (**E**) and the logarithm of the position distribution of histones on the daughter DNA. Source data are provided as a Source Data file.

We performed Repli-pore-seq to analyze the chromatin replication products digested by MNase in our in vitro reconstitution system. We found that 11.6% and 0.5% of the reads were classified as ‘biotinylated’ in the reaction with and without Mcm10 (Mcm10+ and Mcm10 − , respectively) (Fig. 3A). The increased proportion of ‘biotinylated’ reads in the Mcm10+ reaction was consistent with the biotinylated DNA fragments detected on the gel (Fig. 1D). On the other hand, biotinylated DNA fragments were not detected on the gel in the Mcm10− reaction (Fig. 1D). Importantly, the ~0.5% of reads classified as biotinylated in the Mcm10− reaction represent a false-positive background arising from misclassification of unbiotinylated DNA fragments. The false-positive rate is therefore estimated at ~0.5% and is independent of biotin-dUTP incorporation during replication. Based on this, we estimated the fraction of genuinely biotinylated reads in the Mcm10+ reaction (See Methods for details). Hereafter, we assume that all reads in the Mcm10− reaction correspond to parental DNA fragments, and that the reads classified as genuinely biotinylated in the Mcm10+ reaction correspond to daughter DNA fragments.

To get insights into the oligomeric state of the recycled histones, we compared the read length of the parental DNA fragments with that of the daughter DNA fragments. Because DNA fragments shorter than 40 bp are not efficiently recovered during library preparation using the magnetic bead–based protocol, reads shorter than this length were excluded, and we therefore analyzed DNA fragments in the range of 40–400 bp. We observed two distinct peaks in the read-length distribution of parental DNA fragments, corresponding to mono-nucleosome– (144 ± 19 bp) and di-nucleosome–protected fragments (275 ± 31 bp), indicating that nucleosomes are well packed on the DNA substrate prior to replication (Fig. 3B and Supplementary Fig. 3A). The peak read length of daughter DNA fragments was 121 ± 45 bp, shorter than that of parental DNA fragments (Fig. 3B & Supplementary Fig. 3A). Also, the fractions ranging from 40 to 120 bp increased for the daughter DNA fragments (Fig. 3B & Supplementary Fig. 3A). These results suggested that some nucleosomes lost one or two H2A/H2B dimers to form hexasomes or tetrasomes after histone recycling.

Next, we compared the histone positions on the parental and daughter DNA. We used 200 bp or shorter reads for the analysis and defined a midpoint of the read as a position of histones. The position distributions of the parental and daughter DNA fragments were not uniform, but the patterns were reproducible among three repetitive experiments (R = 0.94 ~ 0.96 and 0.97 ~ 0.99, respectively) (Fig. 3C and Supplementary Fig. 3B–D). On the other hand, the position distribution was almost uniform when we sequenced the naked plasmid DNA sheared by sonication (Supplementary Fig. 2H), showing that the read bias was negligible. The less frequent localization of histones around the ACS site supported the successful loading of the replication initiation complex to chromatinized DNA (Fig. 3C). The position distribution of the daughter DNA fragments was moderately correlated with that of the parental DNA fragments (R = 0.70) (Fig. 3D), indicating that histone positions were conserved before and after recycling. Then, we theoretically calculated the free energy landscape of nucleosome formation from the DNA sequence using the Markov-state model^62^ (Fig. 3E) and compared it with the logarithm of the position distribution of the daughter DNA fragments, finding that they are also moderately correlated (R = 0.77) (Fig. 3F). These results suggested that the histones are positioned on the DNA sequences energetically favorable for forming nucleosomes after recycling.

**Fig. 4:**
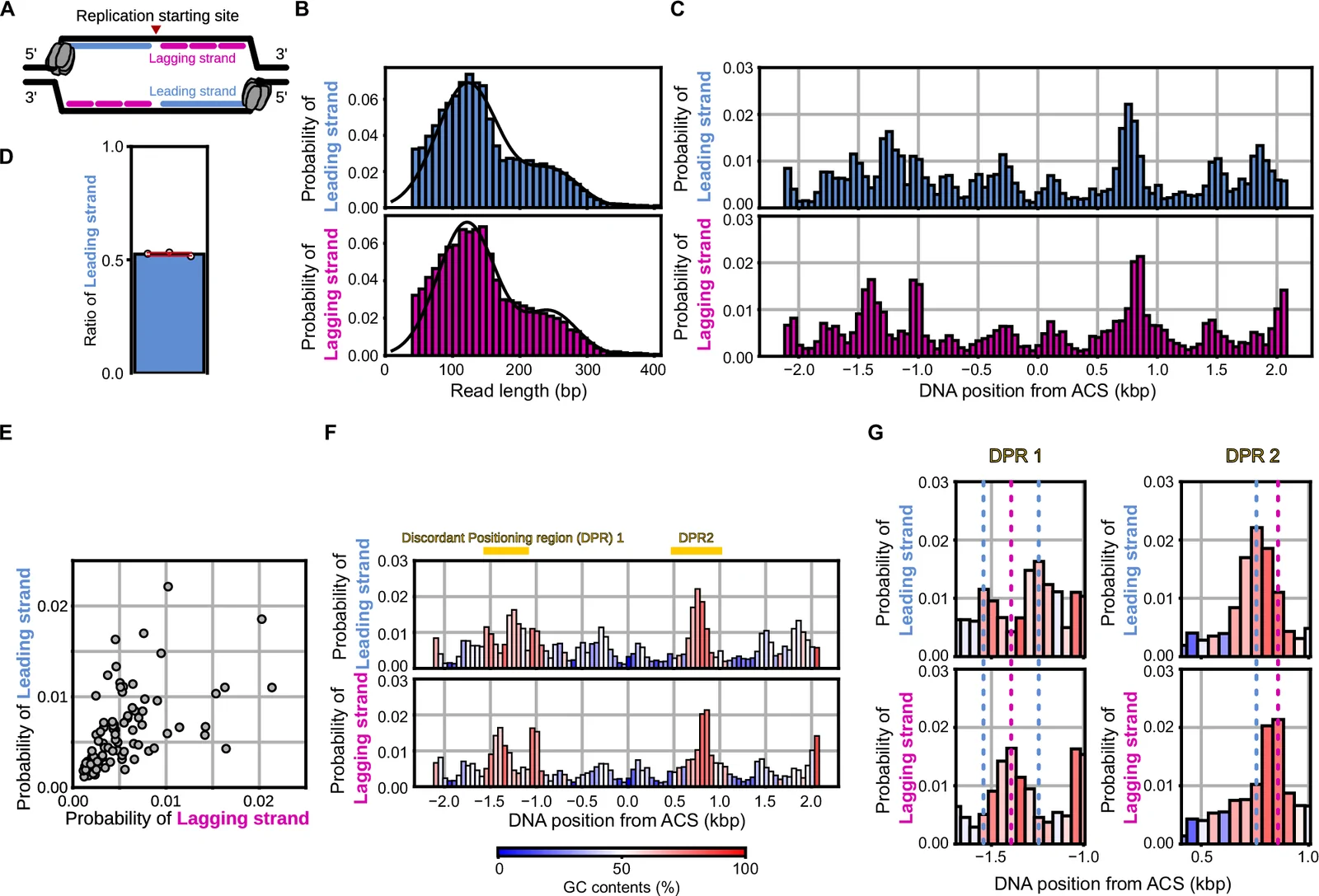
The positions of the recycled histones on the lagging and leading strands. **A** Definition of the leading (cyan) and lagging (magenta) strands. **B** Read-length distribution of fragments derived from the leading (top, cyan) and lagging (bottom, magenta) strands. **C** Position distribution of recycled histones on the leading (top, cyan) and lagging (bottom, magenta) strands. **D** Fraction of leading-strand fragments across three independent experiments. Red error bars indicate mean ± standard deviation. **E** Correlation between the position distributions of recycled histones on the lagging and leading strands. **F** Position distribution of recycled histones on the leading (top) and lagging (bottom) strands, with bin colors indicating GC content. **G** Enlarged views of discordant positioning regions (DPRs) shown in (**F**). Dashed cyan and magenta lines indicate the peak positions of recycled histones on the leading and lagging strands, respectively. Source data are provided as a Source Data file.

Since nanopore sequencing reads single-stranded DNA, we can classify reads of daughter DNA fragments into the lagging and leading strands according to their relative positions from the ACS site (Fig. 4A). The read length distributions of the lagging and leading strand fragments were not significantly different (Fig. 4B). Mapping reads from the leading and lagging strand fragments onto the reference DNA sequence successfully revealed not only the positions of the recycled histones, but also on which strand (leading or lagging) they were recycled, thereby clarifying the recycling symmetry. (Fig. 4C). Interestingly, 52.5 ± 0.7% of the daughter DNA fragments were classified as leading-strand fragments across three independent experiments (Fig. 4D), indicating that histones are recycled almost symmetrically between the lagging and leading strands with slight leading-strand bias in our in vitro reconstitution system, consistent with observations in vivo^19–31^. Based on the correction for misclassified reads (see Methods for details), we estimated that the fraction of reads misclassified as leading-strand fragments was approximately 0.2%. This value is far smaller than the strand-specific recycling biases observed in our experiments (on the order of several percent) and also smaller than the standard deviation across three independent measurements (0.7%), indicating that any distortion of the recycling ratio due to misclassification is negligible. Pairwise correlations of positional distributions among the three replicates were high (R = 0.97–0.99; Supplementary Fig. 4A), whereas the correlation between lagging- and leading-strand fragments was moderate (R = 0.52; Fig. 4E). In three repeated experiments, we reproducibly observed that the recycled positions between lagging and leading strands were roughly similar but not entirely consistent (Fig. 4C–E and Supplementary Fig. 4A).

We focused on two discordant positioning regions (DPRs) in which the recycled positions were reproducibly inconsistent between the lagging and leading strand (Fig. 4F, G and Supplementary Fig. 4B–E). In the DPR1 ( − 1.65 to −1.15 kbp away from the ACS site), we found two peaks on the leading strand and one peak on the lagging strand (Fig. 4G, Supplementary Fig. S4D, E, left). The two peaks on the leading strand were located 100–200 bp away from the single peak on the lagging strand. Moreover, we found that one peak on the lagging strand shifted by ~100 bp away from the peak on the leading strand in the DPR2 (0.55 to 0.95 kbp away from the ACS site) (Fig. 4G and Supplementary Fig. 4D, E, right). Since the DNA sequences of the lagging and leading strands are the same, the free energy landscape of nucleosome formation cannot explain the discordance. Instead, the kinetics of replication progression (e.g., the speed of DNA polymerases) might be a possible factor in the discordant positioning. Interestingly, we noticed that DPRs overlap with GC-rich potential quadruplex-forming sequences where the speed of helicases and DNA polymerases can be slowed down^63^ or uncoupled^64,65^ (Supplementary Fig. 4B–F). Together, we propose that the discordant positioning of nucleosomes on replicated strands reflects uncoupled DNA synthesis kinetics between the lagging and leading strands at GC-rich DNA sequences, which can indirectly influence nucleosome positioning on daughter DNA.

### Removal of Pol δ, Ctf4, Csm3/Tof1, Mrc1, Pol32, and Dpb3/4 affected histone recycling

As shown in Fig. 1C–H, several proteins that are dispensable for chromatin replication (Pol δ, Ctf4, Csm3/Tof1, Mrc1, Pol32, and Dpb3/4) were not strictly required for histone deposition, as sufficient amounts of nucleosome-protected DNA fragments were obtained after replication. To examine the roles of these factors in regulating the symmetry of histone recycling, we performed Repli-pore-seq analyses on replication reactions lacking each factor.

The mean read lengths of lagging-strand fragments in the Pol δ − , Ctf4 − , Csm3/Tof1 − , Mrc1 − , Pol32 − , and Dpb3/4− reactions were 125 ± 43, 128 ± 49, 127 ± 42, 122 ± 41, 132 ± 41, and 127 ± 43 bp, respectively, whereas the corresponding values for leading-strand fragments were 124 ± 40, 118 ± 45, 124 ± 35, 123 ± 40, 131 ± 39, and 125 ± 42 bp (Supplementary Fig. 5A–F). The removal of each factor did not significantly alter the read length, consistent with the gel-electrophoresis-based assays (Fig. 1C–H). The fractions ranging from 40 to 120 bp increased after replication in the same way as the presence of all factors (Figs. 3B, 4B and Supplementary Fig. 5A–F). Therefore, the results suggested that some nucleosomes lost one or two H2A/H2B dimers to form hexasomes or tetrasomes after histone recycling without Pol δ, Ctf4, Csm3/Tof1, Mrc1, Pol32, or Dpb3/4.

**Fig. 5:**
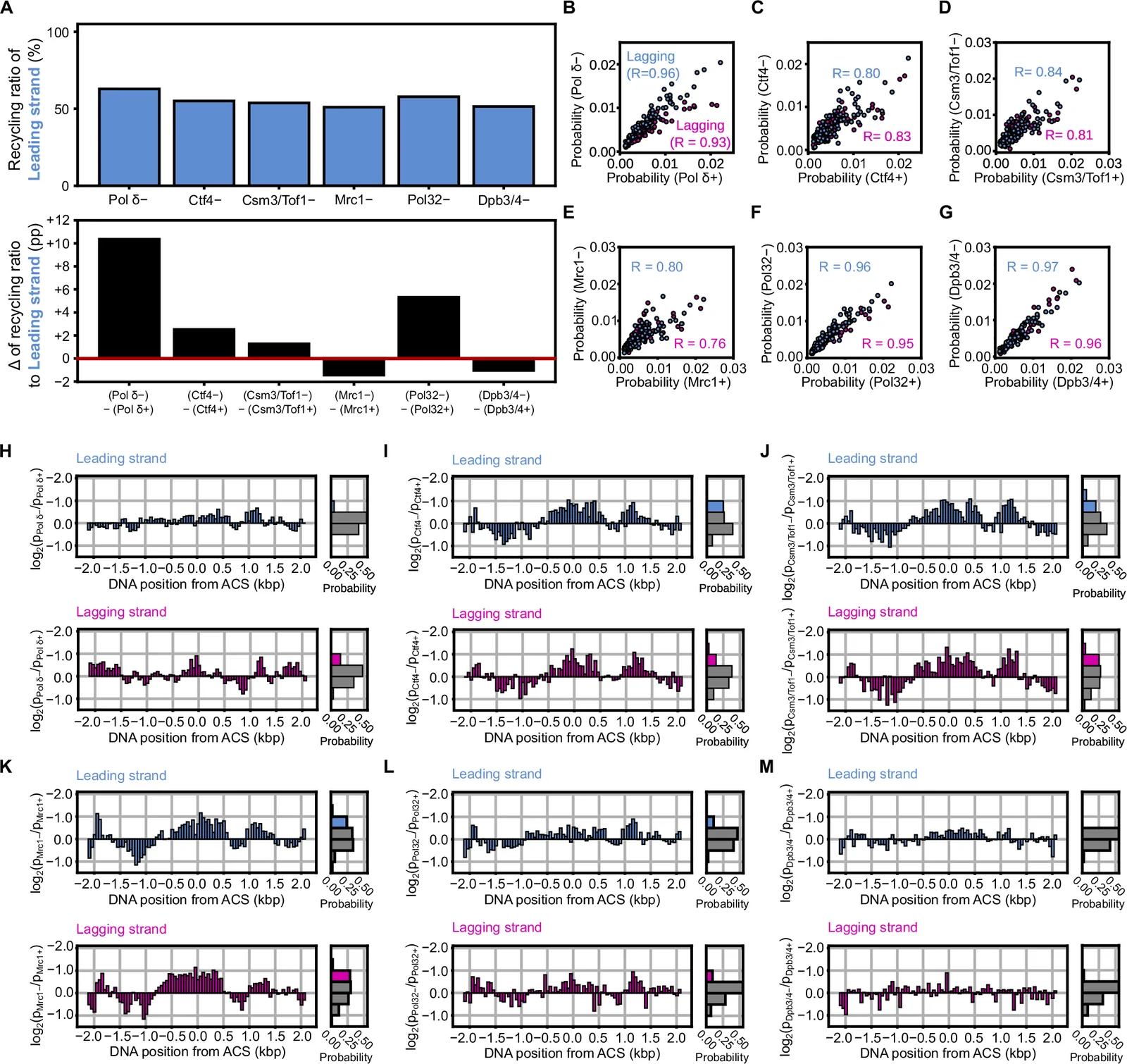
Effects of Pol δ, Ctf4, and Csm3/Tof1 depletion on histone recycling. **A** Fraction of leading-strand fragments in the Pol δ − , Ctf4 − , Csm3/Tof1 − , Mrc1 − , Pol32 − , and Dpb3/4− reactions (top), and the percentage-point differences in the leading-strand fraction between Pol δ− and Pol δ + , Ctf4− and Ctf4 + , Csm3/Tof1− and Csm3/Tof1 + , Mrc1− and Mrc1 + , Pol32− and Pol32 + , and Dpb3/4− and Dpb3/4+ reactions (bottom). Positive and negative values indicate leading- and lagging-strand bias, respectively. **B–G** Correlation plots comparing the position distributions of recycled histones on the lagging and leading strands in the Pol δ− and Pol δ+ reactions (**B**), Ctf4− and Ctf4+ reactions (**C**), Csm3/Tof1− and Csm3/Tof1+ reactions (**D**), Mrc1− and Mrc1+ reactions (**E**), Pol32− and Pol32+ reactions (**F**), and Dpb3/4− and Dpb3/4+ reactions (**G**). **H–M** Logarithmic fold change (log₂FC) in the position distributions of recycled histones on the leading (top) and lagging (bottom) strands for the Pol δ− and Pol δ+ reactions (**H**), Ctf4− and Ctf4+ reactions (**I**), Csm3/Tof1− and Csm3/Tof1+ reactions (**J**), Mrc1− and Mrc1+ reactions (**K**), Pol32− and Pol32+ reactions (**L**), and Dpb3/4− and Dpb3/4+ reactions (**M**). In the histograms, bars are colored gray when log₂FC < 0.5 and cyan (top) or magenta (bottom) when log₂FC ≥ 0.5. Source data are provided as a Source Data file.

Next, we investigated the effects of Pol δ, Ctf4, Csm3/Tof1, Mrc1, Pol32, and Dpb3/4 on the recycling symmetry. Of the genuinely biotinylated DNA fragments in two replicated measurements, 62.9%, 55.0%, 53.8%, 51.1%, 57.8%, and 51.5% were the leading strand fragments in the Pol δ − , Ctf4 − , Csm3/Tof1 − , Mrc1 − , Pol32 − , and Dpb3/4− reactions, respectively (Fig. 5A). We observed the statistically significant leading-strand bias in Pol δ − , Ctf4 − , Csm3/Tof1 − , and Pol32− (*p* < 0.01, Cohen’s d = 7.2, 7.4 × 10^−2^, 1.7 × 10^−2^, and 1.1; two-sided chi-square test) and lagging-strand bias in Mrc1− and Dpb3/4− (*p* < 0.01, Cohen’s d = 0.20, and 0.10; two-sided chi-square test) (Fig. 5A). The observed bias was consistent with previous results of in vivo experiments using *ctf4*Δ^20^, *mrc1*Δ^25^, *pol32*Δ^21,22^, and *dpb3*Δ^24^ mutants of *Saccharomyces cerevisiae*. Despite a different type of mutation, the leading-strand bias in Csm3/Tof1− was consistent with *tof1-3A*, which has mutations in residues interacting with the N-terminal tail of Mcm2. Furthermore, Pol δ − , in which lagging-strand synthesis was compromised, showed more intense lagging-strand bias than Pol32 − , suggesting that a balance between the lagging and leading strand synthesis was also required for symmetric histone recycling.

To examine the effects of Pol δ, Ctf4, Csm3/Tof1, Mrc1, Pol32, and Dpb3/4 on histone recycling positions, we mapped leading- and lagging-strand fragments onto the reference DNA sequence (Supplementary Fig. 5G–L). For both strands, the positional distributions obtained in the Pol δ − , Ctf4 − , Csm3/Tof1 − , Mrc1 − , Pol32 − , and Dpb3/4− reactions showed strong correlations with those observed in the presence of the corresponding factors (Fig. 5B–G). We normalized the position distributions of the lagging- and leading-strand fragments by their read ratios. Then, we calculated the fold change (FC) of the normalized distributions before and after the removal. In the Mrc1 − , Csm3/Tof1 − , Ctf4 − , Pol δ − , Pol32 − , and Dpb3/4− reactions, 31, 30, 18, 14, 10, and 2% of positions on the lagging strand, respectively, and 26, 26, 26, 5, 11, and 0% of positions on the leading strand exhibited log_2_ fold-change (log_2_FC) values greater than 0.5 (Fig. 5H–M). Note that comparisons between independent experiments performed in the presence of all corresponding factors yielded log_2_FC values centered around zero, and only ~0–4% and ~0–8% of positions on the lagging and leading strands, respectively, showed values greater than 0.5, indicating that the estimated nucleosome positions were highly robust and reproducible across independent experiments (Supplementary Fig. 5M–O). After removing Ctf4, Csm3/Tof1, or Mrc1, the log_2_FC values increased over the positions within −0.5 to 0.5 kbp and 1.0 to 1.5 kbp from the ACS site for both the lagging and leading strand fragments (Fig. 5H–M). These regions correspond to the DNA sequences energetically unfavorable for forming nucleosomes (Fig. 3E). Therefore, these results indicate that removal of Ctf4, Csm3/Tof1, or Mrc1 shifts recycled histones toward energetically less favorable positions, suggesting that these factors coordinate DNA sequence–dependent positioning of recycled histones (Fig. 5H–M).

### Lagging strand maturation enhanced histone recycling to the lagging strand

**Fig. 6:**
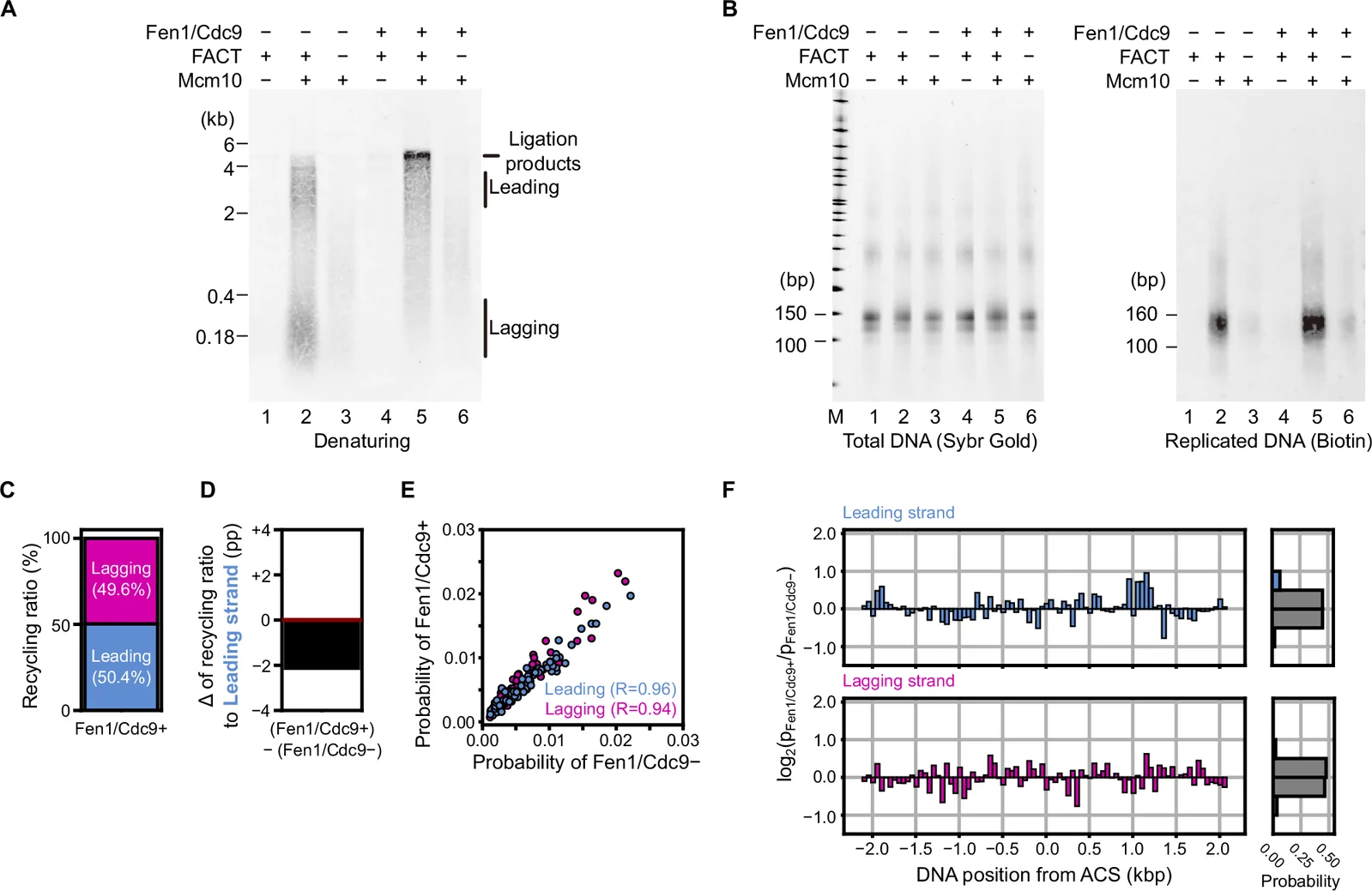
Effects of Fen1/Cdc9 addition on histone recycling. **A** Alkaline denaturing agarose gel showing replicated DNA prior to MNase digestion. ‘M’ denotes a marker (See the Methods). The experiment was repeated twice giving consistent results. **B** Native polyacrylamide gel analysis of total DNA (left) and replicated DNA (right) after MNase digestion. Reactions were performed with Fen1/Cdc9 added during the firing step shown in Fig. 1A. The experiment was repeated twice giving consistent results. **C** Fraction of leading- (cyan) and lagging-strand (magenta) fragments in the Fen1/Cdc9+ reactions. **D** Percentage-point difference in the leading-strand fraction between Fen1/Cdc9+ and Fen1/Cdc9− reactions. Negative values indicate lagging-strand bias. **E** Correlation between the position distributions of recycled histones on the lagging and leading strands in the Fen1/Cdc9+ and Fen1/Cdc9− reactions. **F** Logarithmic fold change (log₂FC) in the position distributions of recycled histones on the leading (top) and lagging (bottom) strands between the Fen1/Cdc9+ and Fen1/Cdc9− reactions. In the histograms, bars are colored gray when log₂FC < 0.5 and cyan (top) or magenta (bottom) when log₂FC ≥ 0.5. Source data are provided as a Source Data file.

Thus far, our in vitro reconstitution system has not included the Okazaki fragment maturation factors Fen1 and Cdc9. To examine the effect of lagging-strand maturation on chromatin replication, we repeated in vitro chromatin replication assays in the presence of Fen1/Cdc9 (Fen1/Cdc9 + ). The disappearance of the 100–200 bp Okazaki fragment bands and the appearance of a ~ 4 kbp ligated DNA band on alkaline denaturing gels indicate successful lagging-strand maturation in the presence of Fen1/Cdc9, even on chromatinized DNA substrates (Fig. 6A, lane 5). Following MNase digestion, nucleosome deposition on replicated DNA was readily detected in the presence of Fen1/Cdc9, similar to that observed in its absence (Fig. 6B, lanes 2 vs 5), indicating that lagging-strand maturation does not compromise histone deposition.

Next, we performed Repli-pore-seq analysis on the Fen1/Cdc9+ reaction to examine how lagging-strand maturation influences the symmetry of histone recycling. Regardless of the presence of Fen1/Cdc9, the read lengths of lagging- and leading-strand fragments were not significantly altered (133 ± 47 bp and 129 ± 42 bp, respectively; Supplementary Fig. 6A). Notably, in the presence of Fen1/Cdc9, 50.4% of biotinylated DNA fragments were classified as leading-strand fragments across two independent measurements (Fig. 6C, D), compared with a higher leading-strand fraction observed in the absence of Fen1/Cdc9 (52.5%), indicating a statistically significant increase in histone recycling to the lagging strand upon lagging-strand maturation (*p* < 0.01, two-sided chi-square test). These results suggest that lagging-strand maturation contributes to enhancing the symmetry of histone recycling.

We further examined the effects of Fen1/Cdc9 on the positions of recycled histones. The positional distributions of recycled histones on the lagging and leading strands were highly correlated between reactions performed in the absence and presence of Fen1/Cdc9 (R = 0.94 and 0.96 for the lagging and leading strands, respectively; Fig. 6E and Supplementary Fig. 6B). Comparison of the normalized distributions revealed that only 2% and 7% of positions on the lagging and leading strands, respectively, exhibited log_2_FC values greater than 0.5 upon addition of Fen1/Cdc9 (Fig. 6F). Together, these results indicate that lagging-strand maturation has only a minimal effect on the positional distribution of recycled histones.

## Discussion

**Fig. 7:**
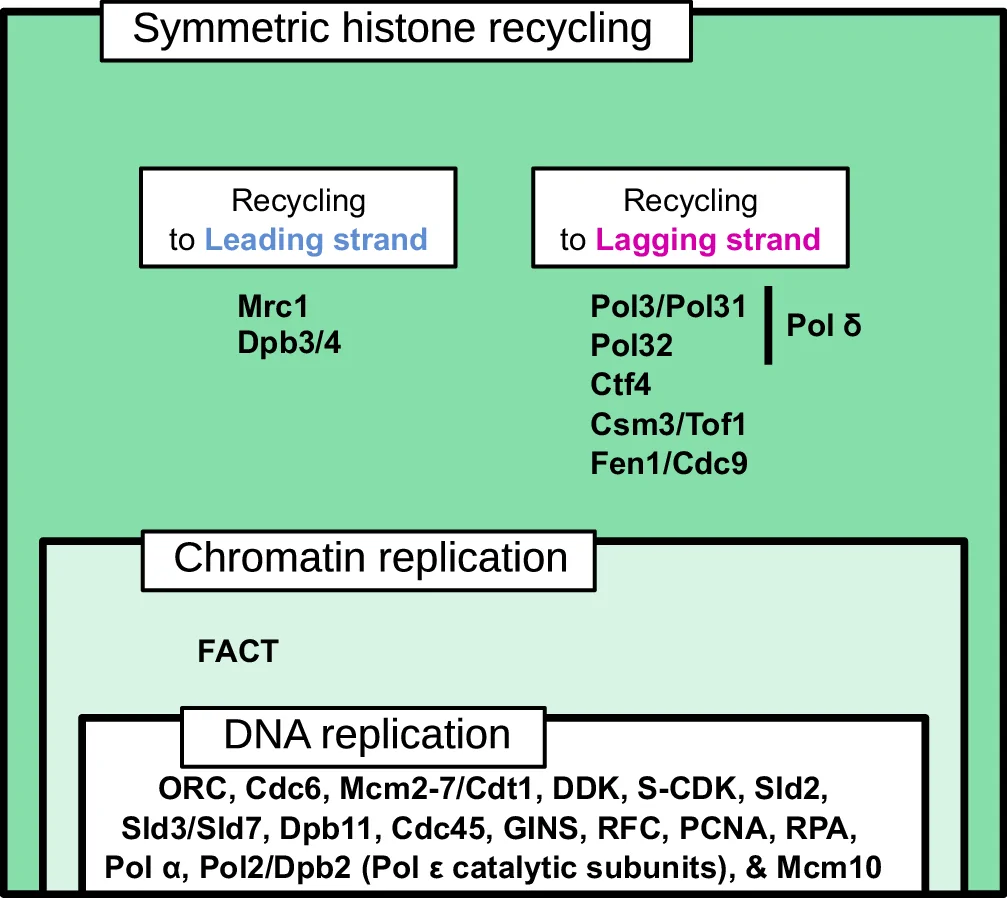
Minimal protein list for chromatin-replication-coupled symmetric histone recycling. Twenty-four purified DNA replication proteins (ORC, Cdc6, Mcm2–7/Cdt1, DDK, S-Cdk, Sld2, Sld3/Sld7, Dpb11, Cdc45, GINS, RFC, PCNA, RPA, Mrc1, Csm3/Tof1, Ctf4, Pol2/Dpb2, Dpb3/4, Pol α, Pol3/Pol31, Pol32, Mcm10, FACT, and Fen1/Cdc9) were identified as the minimal protein set required to reconstitute symmetric histone recycling. Mrc1 and Dpb3/4 contribute to recycling on the leading strand, whereas Pol δ, Pol32, Ctf4, Csm3/Tof1, and Fen1/Cdc9 contribute to recycling on the lagging strand.

In this study, we identified 24 purified proteins as the minimal set required to reconstitute symmetric histone recycling using in vitro chromatin replication and the newly developed Repli-pore-seq method (Fig. 7 and Supplementary Table 1). We found that histones are recycled as hexasomes or tetrasomes. Notably, we observed positional discordance of recycled histones between the two replicated strands in GC-rich regions despite identical DNA sequences. The absence of Pol δ, Ctf4, Csm3/Tof1, Mrc1, Pol32, and Dpb3/4 disrupted the symmetry of histone recycling in a consistent manner with previous in vivo studies. Moreover, we found that Ctf4, Csm3/Tof1, and Mrc1 contribute to precise nucleosome positioning in a manner dependent on the primary DNA sequence (Fig. 7). In addition, we demonstrated that the lagging-strand maturation factors Fen1 and Cdc9 enhance histone recycling to the lagging strand, implying that lagging-strand maturation contributes to maintaining a balanced distribution of parental histones between the lagging and leading strands. The key advance of the present work is that we successfully reconstituted parental histone recycling using only purified components and directly visualized strand-specific recycling toward the leading and lagging strands, thereby enabling (i) identification of the minimal factors required for symmetric histone recycling, (ii) uncoupling histone recycling from subsequent restoration of histone modifications, and (iii) systematic analysis of factor depletion conditions, some of them (i.e. Pol δ and Fen1/Cdc9) are difficult or lethal to examine in cellular systems.

Analysis of read-length distributions obtained by Repli-pore-seq suggests that nucleosomes lose one or two H2A–H2B dimers during histone recycling. Note that, although our computational approach enables recovery of MNase-digested DNA fragments from low-efficiency replication reactions, direct stoichiometric analysis of recycled histones remains technically challenging because the overall efficiency of histone recycling in the in vitro reconstitution is low. Therefore, future studies will require the development of methods that allow efficient isolation of histones specifically associated with replicated DNA to confirm it. Furthermore, in our experimental system, Nap1 and ISW1a, which are required for nucleosome reconstitution, could not be completely removed after the gel-filtration step. Nap1 is known to dismantle H2A–H2B dimers from partially unwrapped nucleosomes^66^ and to deposit H2A–H2B dimers onto tetrasomes^67,68^. Consequently, residual Nap1 may contribute to H2A–H2B recycling and thereby modulate the relative proportions of tetrasomes, hexasomes, and nucleosomes formed on replicated DNA. Future Repli-pore-seq analyses with improved control of Nap1 carryover would provide further insight into the molecular mechanisms of H2A–H2B recycling, which underlie short-term memory in the restoration of chromatin epigenetic states^69^.

Previous studies have identified multiple proteins—including Mcm2, Pol α, Ctf4, Mrc1, Tof1, Pol32 (Pol δ), FACT, PCNA, and Dpb3/4 (Pol ε)—that cooperatively ensure symmetric histone recycling during DNA replication^17,19–31^. In the present study, the Repli-pore-seq analysis successfully recapitulated the strand-specific phenotypes reported in vivo using mutants of these replication factors. Moreover, recent work has proposed extensive functional crosstalk among these proteins in coordinating histone recycling^20–23,25–31^. In those studies, they quantified the absolute density of recycled histones on replicated DNA in combinatorial mutants and demonstrated additive effects on histone recycling, supporting the coexistence of multiple recycling pathways. In contrast, the current Repli-pore-seq procedure is primarily designed to measure the relative strand bias of parental histone recycling rather than the absolute amount of recycled histone. Because our workflow omits the spike-in DNA normalization step, the total read count is influenced by sequencing yield and library recovery efficiency, making rigorous comparison of absolute histone recycling levels between samples difficult. Although omitting the spike-in step maximizes the recovery of low-abundance biotinylated reads from inefficient in vitro replication reactions, it limits quantitative assessment of global decreases in histone recycling efficiency. Future improvements to the Repli-pore-seq procedure, including robust spike-in normalization, together with comprehensive analyses of combinatorial mutants, will be important for fully elucidating the interaction network that governs parental histone recycling during chromatin replication.

Faithful epigenetic inheritance is essential for the maintenance of heterochromatin through generations^13,25,26^. Histone recycling is the first step of heterochromatin maintenance, followed by incorporating newly synthesized histones, restoring epigenetic marks, and reorganizing chromatin structure. The subsequent processes of chromatin maturation require additional proteins, such as histone chaperones (CAF1 and Asf1), histone-modifying enzymes, and proteins to organize chromatin (HP1, linker histones, and SMC-proteins)^4–7^. A combination of in vitro chromatin replication reconstitution with Repli-pore-seq allowed adding proteins to or removing those from the system and analyzing the chromatin structure. Therefore, the pipeline developed here should be helpful in studying molecular mechanisms of chromatin maturation.

The analyses of the recycling positions revealed discordance between the lagging and leading strands at GC-rich and G-quadruplex-prone regions. Previous studies showed the slowing down of DNA polymerases and helicase/polymerase uncoupling in GC-rich regions^63–65^. Moreover, recent studies have suggested that recycling symmetry is modulated by the rate balance of the lagging and leading DNA synthesis^47,70^. These results naturally lead to the hypothesis that the discordance in recycling positions reflects uncoupling between lagging- and leading-strand synthesis. Future Repli-pore-seq analyses using DNA substrates with distinct GC contents would further test this hypothesis. In addition, biophysical approaches that directly visualize replication dynamics, such as single-molecule fluorescence imaging and molecular dynamics simulations, could provide complementary means to evaluate the underlying mechanisms.

Furthermore, removal of Mrc1, Csm3/Tof1, or Ctf4 increased the abundance of recycled histones at energetically less favorable positions compared with reactions containing these factors. As shown in Fig. 3F, the recycled positions are largely influenced by DNA sequence, which determines the thermodynamic landscape of DNA bending and nucleosome formation. However, because parental histone recycling at the replication fork occurs in a highly dynamic non-equilibrium environment, the final nucleosome positions can deviate from the ideal sequence-dependent distribution. Our previous molecular simulation study^47^ suggested that the initial deposition positions of recycled histones are influenced by the local conformational dynamics of DNA and histones that transiently probe each other around the replication fork. Previous studies^20,25–27,48^ also suggested that Csm3/Tof1, Ctf4, and Mrc1 interact with histones or histone chaperones during DNA replication. Therefore, omission of these factors could alter the kinetics and timing of histone transfer and deposition, thereby shifting the final distribution of recycled histones. Future direct visualization of replication-coupled histone recycling dynamics will be important to test whether the kinetics of histone deposition modulate the final nucleosome positions.

Repli-pore-seq analysis of reactions containing Fen1 and Cdc9, which constitute a major pathway for Okazaki fragment maturation, showed enhanced histone recycling to the lagging strand. In contrast, a previous study^22^ reported that deletion of Fen1 in *Saccharomyces cerevisiae* did not alter the strand bias of parental histone recycling, which appears inconsistent with our result. In eukaryotic cells, however, flap structures generated during lagging-strand synthesis are processed not only by Fen1 but also by the Dna2-dependent pathway, which functions redundantly in Okazaki fragment maturation^71^. Thus, a single deletion of Fen1 or Dna2 in cells is not directly comparable to the Fen1/Cdc9− condition in our reconstituted system, which lacks this Okazaki fragment maturation module. Complete evaluation of the contribution of lagging-strand maturation to histone recycling in vivo would require simultaneous disruption of these redundant pathways. However, because Dna2 deletion causes lethality^71^, comprehensive analysis of lagging-strand maturation defects and their impact on histone recycling is technically challenging in cellular systems. In contrast, the in vitro reconstituted system bypasses such cellular constraints and enabled us to directly demonstrate that perturbation of Okazaki fragment maturation alters parental histone recycling patterns, thereby providing mechanistic insight into the relationship between lagging-strand maturation and histone recycling.

Although the addition of Fen1/Cdc9 increased histone recycling to the lagging strand, nucleosome assembly on the lagging strand was still observed in the absence of Fen1/Cdc9. This finding indicates that histone recycling can occur prior to completion of lagging-strand maturation. During Okazaki-fragment (OF) maturation, Pol δ extends an OF and displaces the 5′ end of the adjacent OF to generate a flap structure, which is subsequently removed by Fen1, followed by ligation of the fragments by Cdc9. Faithful processing of Okazaki fragments is essential for maintaining genome integrity. Previous studies have shown that Okazaki fragment junctions are enriched near the nucleosome dyad and that their distribution can be altered by defects in chromatin assembly^72–74^. Together, these observations suggest that histone recycling and lagging-strand maturation reciprocally influence one another to preserve genetic and epigenetic information during DNA replication.

The Repli-pore-seq pipeline combines the reconstituted eukaryotic DNA replication and computational “purification” of the replicated DNA using deep learning-based classification. In vitro reconstitution studies offer an advantage for investigating drug treatments as well as protein mutations or depletions that can cause pleiotropic or lethal effects in cellular systems. On the other hand, the reconstitution has limitations in reaction scale and efficiency. Even a single biochemical purification step after the reconstitution reaction sometimes has a detrimental effect on the yield of the subsequent analysis. Our computational purification procedure allows us to bypass immunoprecipitation, which resulted in the loss of replicated DNA in our initial trial. Furthermore, previous studies have shown that incorporation of nucleotide analogs, such as BrdU and EdU, also induces characteristic perturbations in nanopore current signals and enables reliable detection of nascent DNA^60,61^. These findings support the notion that the computational purification strategy developed here is, in principle, transferable to other experimental systems employing distinct nucleotide analogs. At the same time, because the classifier in the present study was trained on a defined DNA sequence and a specific analog (biotin-dUTP), application to different sequence contexts or analogs would require fine-tuning of the model using appropriate training data. Taken together, the Repli-pore-seq pipeline expands the scope of sequencing-based analyses to defined in vitro reconstitution systems, providing insights that may be difficult to obtain in vivo due to the pleiotropic effects of genetic perturbations or drug treatments.

## Methods

### Material preparations

The 4.1 kbp plasmid, pARS1-4.1^46,77^, was used as the DNA substrate of in vitro replication assays. The DNA fragments to make training data for the deep-learning model were amplified with primers 5’-CCATTTGTCTCCACACCTCC-3’ and 5’-CCATTTGTCTCCACACCTCC-3’ by PCR. The biotinylated fragments were amplified in the reaction containing 200 μM each of dGTP, dCTP, and dATP, and 100 μM each of dTTP and biotin-dUTP. The budding yeast histone octamer (H3, H4, H2A, and H2B) ISW1a (Isw1, Ioc3), Nap1, FACT (Spt16, Pob3), Nhp6A and replication proteins (ORC, Cdc6, Mcm2-7/Cdt1, DDK, S-CDK, Sld2, Sld3/7, Dpb11, Cdc45, GINS, PCNA, RFC, Mcm10, RPA, DNA polymerase α, DNA polymerase δ, DNA polymerase ε, Ctf4, Csm3/Tof1, Mrc1, Top1 and Fen1/Cdc9) were purified as described previously^46^. In brief, histones, Nap1, PCNA, Nhp6A, Mcm10 and Fen1 were expressed in Escherichia coli, and the other proteins were expressed in Saccharomyces cerevisiae. Cell lysates were prepared by sonication or cryogenic grinding, respectively, followed by ultracentrifugation. Replication proteins were then purified as described below.

### Protein purification

#### Histone octamer

Native histone octamer was purified sequentially using Heparin Sepharose FF (Cytiva) and a Superdex 200 (Cytiva) gel filtration column, followed by recovery of the flow-through fractions from hydroxyapatite chromatography.

#### ISW1a

ISW1a, containing C-terminally TAP-tagged Ioc3, was affinity-purified using Human IgG Sepharose (Cytiva) and eluted by TEV protease cleavage. The eluate was further purified using Calmodulin Sepharose 4B (Cytiva), eluted with EGTA, and then fractionated on an SP-XL (Cytiva) column.

#### Nap1

N-terminally GST-tagged Nap1 was purified using Glutathione Sepharose 4B (Cytiva) and eluted with glutathione. After removal of the GST tag by 3 C protease cleavage, the protein was further purified on Resource Q (Cytiva) and Superdex 200 columns.

#### FACT

FACT, containing N-terminally 12xHis-tagged Pob3, was purified sequentially using Heparin Sepharose FF (Cytiva) and Ni-NTA agarose (Qiagen). After imidazole elution, the complex was further purified on Q-XL (Cytiva) and Superdex 200 columns.

#### Nhp6A

Native Nhp6A was subjected to trichloroacetic acid precipitation and further purified on a Resource S (Cytiva) column.

#### ORC

ORC, carrying an N-terminal 3xFLAG tag on Orc1, was affinity-purified using anti-FLAG M2 agarose (Merck) and eluted by 3 C protease cleavage. The eluate was further fractionated on Resource Q and Resource S columns.

#### Cdc6

N-terminally 6xHis-tagged Cdc6 was precipitated with ammonium sulfate and purified using Ni-NTA agarose. After imidazole elution, the protein was further purified on a Resource S column.

#### Mcm2-7/Cdt1

Cdt1-Mcm2-7, carrying a C-terminal 5xFLAG tag on Mcm4, was affinity-purified using anti-FLAG M2 agarose and eluted by 3 C protease cleavage. The eluate was further purified by Superose 6 (Cytiva) size-exclusion chromatography.

#### DDK

DDK, containing N-terminally 3xFLAG-tagged Dbf4 and N-terminally 10xHis-tagged Cdc7, was affinity-purified using anti-FLAG M2 agarose and eluted with FLAG peptide. The complex was further purified on Heparin HP (Cytiva) and Resource Q columns.

#### S-CDK

S-CDK, containing N-terminally CBP-tagged Clb5, was purified using Calmodulin Sepharose 4B and eluted by TEV protease cleavage. The eluate was further purified on Resource Q and Superdex 200 columns.

#### Sld2

N-terminally 3xFLAG-tagged Sld2 was affinity-purified using anti-FLAG M2 agarose and eluted by 3 C protease cleavage. The eluate was further purified on Heparin HP and Phenyl (Cytiva) columns.

#### Sld3/7

Sld3-Sld7, containing C-terminally 5xFLAG-tagged Sld3, was affinity-purified using anti-FLAG M2 agarose and eluted by 3 C protease cleavage. The eluate was further purified by Superdex 200 size-exclusion chromatography.

#### Dpb11

C-terminally 3xFLAG-tagged Dpb11 was affinity-purified using anti-FLAG M2 agarose and eluted with FLAG peptide. The protein was further purified on Heparin HP and SP-XL columns.

#### Cdc45

Cdc45, containing an internal 2xFLAG tag, was affinity-purified using anti-FLAG M2 agarose and eluted with FLAG peptide. The protein was further purified on a hydroxyapatite column.

#### GINS

GINS, containing N-terminally 3xFLAG-tagged Psf1, was affinity-purified using anti-FLAG M2 agarose and eluted by 3 C protease cleavage. The eluate was further purified on Resource Q and Resource S columns.

#### PCNA

Native PCNA was subjected to Polymin P precipitation, followed by ammonium sulfate precipitation. The protein was then further purified on a Resource Q column.

#### RFC

RFC, containing C-terminally 2xProtein A-tagged Rfc1, was affinity-purified using rabbit IgG agarose (Merck) and eluted by 3 C protease cleavage. The complex was further purified on Capto S (Cytiva) and Superdex 200 columns.

#### Mcm10

N-terminally 6xHis-tagged Mcm10 was purified using Ni-NTA agarose and eluted with imidazole. After removal of the His tag by 3 C protease cleavage, the protein was further purified on a Resource S column.

#### RPA

Native RPA was purified sequentially using Affi-Gel Blue (Bio-Rad), hydroxyapatite, and Resource Q columns.

#### Pol α

Pol α, containing N-terminally CBP-tagged Pri1, was purified using Calmodulin Sepharose 4B and eluted with EDTA/EGTA. The complex was further purified on Heparin HP, Resource Q, and Superose 6 columns.

#### Pol δ

Pol δ, containing N-terminally 3xFLAG-tagged Pol3, was affinity-purified using anti-FLAG M2 agarose and eluted by 3 C protease cleavage. The eluate was further purified on Heparin HP, Resource S, and Resource Q columns.

#### Pol ε

Pol ε, containing C-terminally 5xFLAG-tagged Dpb3, was affinity-purified using anti-FLAG M2 agarose and eluted by 3 C protease cleavage. The eluate was further purified on Heparin HP and Resource Q columns.

#### Ctf4

C-terminally 2xProtein A-tagged Ctf4 was affinity-purified using rabbit IgG agarose and eluted by 3 C protease cleavage. The eluate was further purified on Resource Q and Superdex 200 columns.

#### Csm3/Tof1

Csm3/Tof1, containing C-terminally 2xProtein A-tagged Csm3, was affinity-purified using rabbit IgG agarose and eluted by 3 C protease cleavage. The complex was further purified on Resource Q and Superdex 200 columns.

#### Mrc1

C-terminally 5xFLAG-tagged Mrc1 was affinity-purified using anti-FLAG M2 agarose and eluted with FLAG peptide. The protein was further purified by Superdex 200 size-exclusion chromatography.

#### Top1

N-terminally 2xProtein A-tagged Top1 was affinity-purified using rabbit IgG agarose and eluted by 3 C protease cleavage. The eluate was further purified on Heparin HP and Superdex 200 columns.

#### Fen1

C-terminally 6xHis-tagged Fen1 was purified using TALON agarose and eluted with imidazole. The protein was further purified on Heparin HP and Superdex 200 columns.

#### Cdc9

C-terminally 2xProtein A-tagged Cdc9 was affinity-purified using rabbit IgG agarose and eluted by 3 C protease cleavage. The eluate was further purified on Heparin HP and Superdex 200 columns.

### In vitro reconstitution of chromatin replication

The nucleosomes were reconstituted by mixing the pARS1-4.1 plasmid DNA (4 nM) with histone octamers (400 nM), ISW1a (17.3 nM), Nap1 (3 μM), and ATP as described previously^46^. After the nucleosome assembly, we exchanged the buffer and removed residual proteins using the MicroSpin S-400 HR column (Cytiva). Then, we assembled MCM double hexamer onto the chromatinized DNA substrates by adding 20 nM ORC, 50 nM Cdc6, and 60 nM Mcm2-7/Cdt1 in the MCM loading buffer [25 mM HEPES-KOH (pH 7.5), 100 mM KOAc, 1 mM DTT, 5 mM ATP, 7.5 mM Mg(OAc)_2_, 5% glycerol, 0.01% (w/v) NP-40, and 0.1 mg/mL BSA]. After 15 minutes of incubation at 30 °C, 40 nM DDK was added to the reaction and further incubated for 15 minutes. Following the MCM loading, the reaction was diluted three-fold in the replication buffer [25 mM HEPES-KOH (pH 7.5), 100 mM KOAc, 1 mM DTT, 5 mM ATP, 7.5 mM Mg(OAc)_2_, 5% glycerol, 0.01 % (w/v) NP-40, 0.1 mg/mL BSA, 0.1 mM CTP, 0.1 mM GTP, 0.1 mM TTP, 40 μM dATP, 40 μM dCTP, 40 μM dGTP, 25 μM dTTP, and 15 μM biotin-dUTP], and mixed with 5 nM S-CDK, 30 nM Sld2, 20 nM Sld3/7, 30 nM Dpb11, 40 nM Cdc45, 12.5 nM GINS, 20 nM Csm3/Tof1, 20 nM Ctf4, 10 nM Mrc1, 20 nM RFC, 20 nM PCNA, 100 nM RPA, 5 nM Pol α, 10 nM Pol δ, and 20 nM Pol ε, 6.7 nM ORC, 16.7 nM Cdc6, and 20 nM Mcm2-7/Cdt1, 13.3 nM DDK. We started the replication reaction by adding a histone chaperone FACT (40 nM FACT and 200 nM Nhp6A) and a firing factor Mcm10 (5 nM) to the reaction mixture and incubated at 30 °C for 20 min. After the replication reactions, we adjusted the salt concentration to 50 mM NaCl and 2 mM CaCl_2_ and digested the replication products by adding 1 U/μL of Micrococcal nuclease (MNase) and placing it at 30 °C for 30 min. Then, the reaction was mixed with 15 mM EDTA, 0.1% (w/v) SDS, and 0.5 mg/mL protease K and incubated at 37 °C for 20 min to stop the reaction.

For native polyacrylamide gel electrophoresis, the samples were mixed with the 6× loading buffer [15% (w/v) ficol, 10 mM HEPES-KOH (pH 7.5), 0.05% Orange G] and were applied to 7.5% polyacrylamide gel in EzRun TG buffer (ATTO) at 21 mA at room temperature for 65 min. The gel was soaked in 0.5× TBE for 5 min, and the DNA fragments were transferred to the Zeta-Probe membrane (Bio-Rad) using a wet-transfer blotter at 80 V at 4 °C for 50 min. The transferred membrane was crosslinked using a UV illuminator and soaked in the blocking solution (Cytiva) at room temperature for 20 min. The membrane was incubated with Dylight680-conjugated streptavidin in the TBS-T buffer [TBS containing 0.1% (w/v) Tween 20 and 0.01% (w/v) SDS] for 30 min, followed by washing the membrane three times with the TBS-T buffer. Gel images were captured by ChemiDoc Touch imager (Bio-Rad).

For denaturing agarose gel electrophoresis, the sample was mixed with 50 mM EDTA and the 6× loading buffer [0.3 M NaOH, 6 mM EDTA, 36% (w/v) Glycerol, 0.25% (w/v) Orange G] and was applied to 0.8% agarose gel in the alkaline buffer (50 mM NaOH, 1 mM EDTA) at 3.6 V/cm at 4 °C for 2 h. The gel was dipped in 0.5× TBE for 10 min, and the DNA fragments were transferred to the nylon membrane (Hybond-N + , Cytiva) using a wet-transfer blotter at 80 V at 4 °C for 50 min. The biotinylated DNA fragments were detected as described above.

Biotinylated DNA markers used in the denaturing agarose gel were prepared as previously described^46^. Biotinylated DNA markers used for native PAGE gel analysis (100 and 160 bp) were prepared by PCR amplification using yeast genomic DNA as a template with a pair of primers for 100 bp (100/160 F: 5’-biotin-GGTGTATGCATGCTACTGTTTGAATTCCCATTATCGAAGGCAC-3’ and 100 R: 5’-AAATAAAAATGAGTATCCCCGCTGAC-3’) and a different pair of primers for 160 bp (100/160 F and 160 R: 5’-TAGATTGATCTTGAAAGCCAG-3). DNA marker used for Sybr Gold staining of PAGE gels was purchased from Nippon Genetics (FastGene 50 bp DNA Ladder, MWD50).

### Nanopore sequencing

For nanopore sequencing, the DNA fragments digested by MNase were purified in sterilized water using the silica membrane spin column (Qiagen). We repaired the purified DNA fragments using the NEBNext^®^ Companion Module for Oxford Nanopore Technologies^®^ Ligation Sequencing (New England Biolabs) and ligated the adapter proteins to the repaired DNA fragments using the Ligation Sequencing Kit V14 (SQK-LSK 114, Oxford Nanopore Technologies^®^). We loaded the DNA library onto the MinION R10.4.1 flowcell (FLO-MIN 114, Oxford Nanopore Technologies^®^) and ran the sequencing on MinKNOW (Oxford Nanopore Technologies^®^). The current signals were translated into nucleotide sequences using the Guppy software (Oxford Nanopore Technologies^®^).

### Deep-learning model training

We used the current signals of the PCR-amplified biotinylated and unbiotinylated DNA substrates as training data for our deep-learning model. We segmented the current signals based on a stepwise change, calculated each segment’s mean, standard deviation, and duration time, and converted the signals into an array of segments. When the number of segments in one array exceeded 600, the 200–600 subset segments were randomly extracted. Then, we padded the arrays to the size of 3 × 600. The segment arrays derived from unbiotinylated and biotinylated DNA were labeled 0 and 1, respectively. We kept running the sequencing of unbiotinylated and biotinylated DNA so that their sequence coverages were to be (4.8 ± 0.8) × 10^4^ and (6.2 ± 1.3) × 10^4^, respectively. All the obtained current signals were converted into 538,705 arrays, each of unbiotinylated- and biotinylated-DNA, and randomly split the total (2 × 538,705) arrays into 969,669 and 107,741 arrays for training and validation data, respectively.

Each 3 × 600 size array was fed into the depth-wise and point-wise convolution, batch normalization, and ReLU activation (DSC-PSC-BN-ReLU) layers. The output tensors were fed into 8 repeats of the residual block followed by LSTM with 128 units and the max pooling layer. Each residual block contained 4 sets of DSC-PSC-BN-ReLU followed by one DSC-PSC-BN. The output from the network was fully connected and activated by sigmoid functions. We used focal cross entropy as a loss function. The model was trained using Adam optimizer with a learning rate of 0.001 for up to 1000 epochs with a batch size of 1024. We used early stopping of epochs to avoid overfitting to training data. The output values from the final layer below 0.5 were regarded as ‘unbiotinylated’ while those of 0.5 and above were considered ‘biotinylated.’

### Correction for misclassified reads

In the Mcm10− reactions, ~0.6% of the reads were misclassified as ‘biotinylated’ (Supplementary Fig. 2I). Thus, the biotinylated reads should be a mixture of genuinely biotinylated reads and misclassified unbiotinylated reads. To obtain an ensemble of genuinely biotinylated reads, we need to subtract the contribution of the misclassified reads from an ensemble of biotinylated reads in the Mcm10+ reactions.

We define the number of total reads, biotinylated reads, unbiotinylated reads, misclassified biotinylated reads, genuinely biotinylated reads, and genuinely unbiotinylated reads as N, ${N}_{{Positive}}$, ${N}_{{Negative}}$, ${N}_{{False}-{Positive}}$, ${N}_{{Biotinylated}}$, and ${N}_{{Unbiotinylated}}$, respectively. Then, we have the following relationships.

1 $$N={N}_{{Positive}}+{N}_{{Negative}}$$

2 $$N={N}_{{Biotinylated}}+{N}_{{Unbiotinylated}}$$

Based on the assumption that biotinylated reads are a mixture of truly biotinylated reads and misclassified unbiotinylated reads, the following relationship holds.

3 $${N}_{{Positive}}={N}_{{Biotinylated}}+{N}_{{False}-{Positive}}$$

The ratios of ‘biotinylated’ reads, misclassified unbiotinylated reads, and truly biotinylated reads to total reads (${r}_{{Positive}}$, ${r}_{{False}-{Positive}}$, and ${r}_{{Biotinylated}}$) are given by,

4 $${r}_{{Positive}}=\frac{{N}_{{Positive}}}{N}=\frac{{N}_{{Biotinylated}}}{N}+\frac{{N}_{{False}-{Positive}}}{N}={r}_{{Biotinylated}}+{r}_{{False}-{Positive}}$$

5 $${r}_{{Biotinylated}}=\frac{{N}_{{Biotinylated}}}{N}$$

6 $${r}_{{False}-{Positive}}=\frac{{N}_{{False}-{Positive}}}{N}$$

Here, we assume that the classifier mistakenly classifies genuinely unbiotinylated reads as biotinylated reads at a ratio α. Then, the number and ratio of misclassified unbiotinylated reads are given by,

7 $${N}_{{False}-{Positive}}=\alpha {N}_{{Unbiotinylated}}$$

8 $${r}_{{False}-{Positive}}=\alpha \frac{{N}_{{Unbiotinylated}}}{N}=\alpha \frac{N-{N}_{{Biotinylated}}}{N}=\alpha \left(1-{r}_{{Biotinylated}}\right)$$

From Eqs. 4 and 8, we can derive the following relationships:

9 $${r}_{{Positive}}={r}_{{Biotinylated}}+\alpha \left(1-{r}_{{Biotinylated}}\right)$$

10 $${r}_{{Biotinylated}}=\frac{{r}_{{Positive}}-\alpha }{1-\alpha }$$

In the Mcm10− reaction, all reads should be unbiotinylated ones. Then, ${r}_{{Biotinylated}}$ is equal to 0 in Eq. 9, and the following relationship holds.

11 $$\alpha={r}_{-,{Positive}}={r}_{-,{False}-{Positive}}$$

Next, we estimated an ensemble of truly biotinylated reads. In this study, we obtained the position distributions of the DNA fragments classified as biotinylated in the Mcm10+ reaction. As shown in Supplementary Fig. 2I, we also obtained the position distributions of the DNA fragments misclassified as biotinylated in the Mcm10− reaction. We need to subtract this misclassification bias from the position distributions of the biotinylated reads. Here, we define the position probabilities of the total, genuine, and misclassified biotinylated reads at position x as ${p}_{{Positive}}\left(x\right)$, ${p}_{{Biotinylated}}\left(x\right)$, and $\beta \left(x\right)$, respectively. Then, the numbers of total [${n}_{{Positive}}\left(x\right)$], genuinely [${n}_{{Biotinylated}}\left(x\right)$], and misclassified [$b\left(x\right)$] biotinylated reads at position x are represented as

12 $${n}_{{Positive}}\left(x\right)={N}_{{Positive}}{p}_{{Positive}}\left(x\right)$$

13 $${n}_{{Biotinylated}}\left(x\right)={N}_{{Biotinylated}}{p}_{{Biotinylated}}\left(x\right)$$

14 $$b\left(x\right)={N}_{{False}-{Positive}}\beta \left(x\right)$$

Here, the following relationships hold as in Eq. 3.

15 $${n}_{{Positive}}\left(x\right)={n}_{{Biotinylated}}\left(x\right)+b\left(x\right)$$

16 $${N}_{{Positive}}{p}_{{Positive}}\left(x\right)={N}_{{Biotinylated}}{p}_{{Biotinylated}}\left(x\right)+{N}_{{False}-{Positive}}\beta \left(x\right)$$

17 $$\frac{{N}_{{Positive}}}{N}{p}_{{Positive}}\left(x\right)=\frac{{N}_{{Biotinylated}}}{N}{p}_{{Biotinylated}}\left(x\right)+\frac{{N}_{{False}-{Positive}}}{N}\beta \left(x\right)$$

18 $${r}_{{Positive}}{p}_{{Positive}}\left(x\right)={r}_{{Biotinylated}}{p}_{{Biotinylated}}\left(x\right)+{r}_{{False}-{Positive}}\beta \left(x\right)$$

From Eqs. 8 and 18, we can derive the following equations.

19 $${r}_{{Positive}}{p}_{{Positive}}\left(x\right)={r}_{{Biotinylated}}{p}_{{Biotinylated}}\left(x\right)+\alpha \left(1-{r}_{{Biotinylated}}\right)\beta \left(x\right)$$

20 $${p}_{{Biotinylated}}\left(x\right)=\frac{{r}_{{Positive}}}{{r}_{{Biotinylated}}}{p}_{{Positive}}\left(x\right)-\frac{\alpha \left(1-{r}_{{Biotinylated}}\right)}{{r}_{{Biotinylated}}}\beta \left(x\right)$$

From Eqs. 10 and 20, we can derive the following equation.

21 $${p}_{{Biotinylated}}\left(x\right)=\frac{{r}_{{Positive}}\left(1-\alpha \right)}{{r}_{{Positive}}-\alpha }{p}_{{Positive}}\left(x\right)+\frac{\alpha \left({r}_{{Positive}}-1\right)}{{r}_{{Positive}}-\alpha }\beta \left(x\right)$$

Again, in the Mcm10− reaction, the ratio of truly biotinylated reads should be 0. Thus, from Eqs. 11 and 19, we can derive the following relationships:

22 $${r}_{-,{Positive}}{p}_{-,{Positive}}\left(x\right)=\alpha {p}_{-,{Positive}}\left(x\right)=\alpha \beta \left(x\right)$$

23 $$\beta \left(x\right)={P}_{-,{Positive}}\left(x\right)$$

Therefore, $\beta \left(x\right)$ can be calculated from the position distributions of the DNA fragments misclassified to be biotinylated in the Mcm10− reaction. Then, we can subtract the contribution of the misclassified reads from an ensemble of biotinylated reads in the Mcm10+ reactions using Eq. 21.

Finally, we estimated the leading strand ratio of truly biotinylated reads. We defined the number of total, genuine, and misclassified leading-strand DNA fragments as ${N}_{{Positive},{Leading}}$, ${N}_{{Biotinylated},{Leading}}$, and ${N}_{{False}-{Positive},{Leading}}$. Also, we define the number of total, genuinely, and misclassified biotinylated reads at position x of Watson and Click strands as [${n}_{{Positive},{Watson}}\left(x\right)$ & ${n}_{{Positive},{Click}}\left(x\right)$], [${n}_{{Biotinylated},{Watson}}\left(x\right)$ & ${n}_{{Biotinylated},{Click}}\left(x\right)$], and [${b}_{{Watson}}\left(x\right)\&{b}_{{Click}}\left(x\right)$]. The number of total, genuinely, and misclassified leading-strand DNA fragments are represented as

24 $${N}_{{Positive},{Leading}}={\sum}_{x\ge 0}{n}_{{positive},{Watson}}\left(x\right)+{\sum}_{x\ge 0}{n}_{{positive},{Watson}}\left(x\right)$$

25 $${N}_{{Biotinylated},{Leading}}={\sum}_{x\ge 0}{n}_{{positive},{Watson}}\left(x\right)+{\sum}_{x\ge 0}{n}_{{positive},{Watson}}\left(x\right)$$

26 $${N}_{{False}-{Positive},{Leading}}={\sum}_{x\ge 0}{b}_{{Watson}}\left(x\right)+{\sum}_{x < 0}{b}_{{Click}}\left(x\right)$$

Here, the following relationships hold as in Eq. 3.

27 $${n}_{{Positive},{Watson}}\left(x\right)={n}_{{Biotinylated},{Watson}}\left(x\right)+{b}_{{Watson}}\left(x\right)$$

28 $${n}_{{Positive},{Click}}\left(x\right)={n}_{{Biotinylated},{Click}}\left(x\right)+{b}_{{Click}}\left(x\right)$$

Thus, as shown below, the relationship in Eq. 3 also holds true.

29 $$	{N}_{{Positive},{Leading}}={\sum}_{x\ge 0}{n}_{{positive},{Watson}}\left(x\right)+{\sum}_{x < 0}{n}_{{positive},{Click}}\left(x\right)\\ 	=\,{\sum}_{x\ge 0}{n}_{{Biotinylated},{Watson}}\left(x\right)+{\sum}_{x\ge 0}{b}_{{Watson}}\left(x\right)+{\sum}_{x < 0}{n}_{{Biotinylated},{Click}}\left(x\right)+{\sum}_{x < 0}{b}_{{Click}}\left(x\right)\\ 	={N}_{{Biotinylated},{Leading}}+{N}_{{False}-{Positive},{Leading}}$$

Thus, when we define the ratios of total, genuinely, and misclassified leading-strand DNA fragments to total reads (${r}_{{Positive},{Leading}}$, ${r}_{{Biotinylated},{Leading}}$, and ${r}_{{False}-{Positive},{Leading}}$), we can derive the following equation in a similar manner to the deviation of Eqs. 1 to 9.

30 $${r}_{{Positive},{Leading}}={r}_{{Biotinylated},{Leading}}+{\alpha }_{{Leading}}\left(1-{r}_{{Biotinylated},{Leading}}\right)$$

In the Mcm10− reaction, all reads should be unbiotinylated ones. Then, ${r}_{{Biotinylated},{Leading}}$ is equal to 0 in Eq. 30, and the following relationship holds

31 $${a}_{{Leading}}={r}_{-,{Positive},{Leading}}={r}_{-,{False}-{Positive},{Leading}}$$

Then, the ratio of misclassified leading-strand DNA fragments to total reads is a difference between the ratios of total and genuinely leading-strand DNA fragments and given by,

32 $$\begin{array}{c}{r}_{{Positive},{Leading}}-{r}_{{Biotinylated},{Leading}}\\={\alpha }_{{Leading}}\left(1-{r}_{{Biotinylated},{Leading}}\right)\\={r}_{{False}-{Positive},{Leading}}\left(1-{r}_{{Biotinylated},{Leading}}\right)\end{array}$$

### Prediction of potential quadruplex-forming sequence

The quadruplex-forming sequences (PQS) were predicted using pqsfinder^75^. The PQS was searched as four guanine runs (G-runs). Each G-run was defined as a stretch of 2 to 20 guanines, with one insertion of a stretch of 0–9 other nucleotides allowed. The score function to evaluate the PQS site is based on the reward for G-tetrad stacking and on the penalty for mismatches and the insertion in G-runs. The parameters were calibrated by maximizing the correlation between the signal from the G4-seq dataset^76^ and the score.

### Reporting summary

Further information on research design is available in the Nature Portfolio Reporting Summary linked to this article.

## Supplementary information

Supplementary Information

Reporting Summary

Transparent Peer Review file

## Source data

Source Data

## Data Availability

The data that support the findings of this study are available within the article and Supplementary Information files. The plasmid DNA sequence for pARS1-4.1 can be found at GitHub (https://github.com/TerakawaLab/2024_Nagae)^77^. The Repli-pore-seq data generated in this study have been deposited in NCBI’s Sequence Read Archive (SRA) with the Bioproject accession code PRJNA1498125 [https://www.ncbi.nlm.nih.gov/bioproject/1498125]. A list of the SRA accession codes is provided in Supplementary Table 2. Source data are provided with this paper.

## Code availability

All the codes for the deep-learning based classifier can be found at GitHub (https://github.com/TerakawaLab/2024_Nagae)^77^.
