# Supplementary material for: In vitro reconstitution of chromatin replication recapitulates symmetric histone recycling
Source: Nat Commun. 2026 Aug 11;17:9627. doi: 10.1038/s41467-026-76520-5 (PMC13554108; doi:10.1038/s41467-026-76520-5)
Supplement: Supplementary file 1 [file 41467_2026_76520_MOESM1_ESM.pdf]

SUPPLEMENTARY INFORMATION

Supplementary Table 1

Name of purified protein	Subunits	Role in histone recycling
**ORC**	Orc1, Orc2, Orc3, Orc4, Orc5, Orc6	DNA replication
**Cdc6**	Cdc6	DNA replication
**Mcm2-7/Cdt1**	Mcm2, Mcm3, Mcm4, Mcm5, Mcm6, Mcm7, Cdt1	DNA replication
**DDK**	Cdc7, Dbf4	DNA replication
**S-CDK**	Clb5, Cdc28, Cks1	DNA replication
**Sld2**	Sld2	DNA replication
**Sld3/Sld7**	Sld3, Sld7	DNA replication
**Dpb11**	Dpb11	DNA replication
**Cdc45**	Cdc45	DNA replication
**GINS**	Sld5, Psf1, Psf2, Psf3	DNA replication
**RFC**	Rfc1, Rfc2, Rfc3, Rfc4, Rfc5	DNA replication
**PCNA**	PCNA	DNA replication
**RPA**	Rpa1, Rpa2, Rpa3	DNA replication
**Pol α**	Pol1, Pol12, Pri1, Pri2	DNA replication
**Pol ε**	Pol2, Dpb2	DNA replication
	Dpb3, Dpb4	Recycling to the leading strand
**Mcm10**	Mcm10	DNA replication
**FACT**	Spt16, Pob3	Chromatin replication
**Pol δ**	Pol3, Pol31	Recycling to the lagging strand
	Pol32	Recycling to the lagging strand
**Csm3/Tof1**	Csm3, Tof1	Recycling to the lagging strand
**Ctf4**	Ctf4	Recycling to the lagging strand
**Mrc1**	Mrc1	Recycling to the leading strand
**Fen1/Cdc9**	Fen1, Cdc9	Recycling to the leading strand

Supplementary Table 2

Sequence Read Archive Accession Code	Description
SRR39748826	Total reads of WT and Mcm10− condition
SRR39748820	Total reads of Pol δ− and Mcm10− condition
SRR39748816	Total reads of Ctf4− and Mcm10− condition
SRR39748818	Total reads of Csm3/Tof1− and Mcm10− condition
SRR39748824	Total reads of Mrc1− and Mcm10− condition
SRR39748813	Total reads of Dpb3/4− and Mcm10− condition
SRR39748822	Total reads of Pol32− and Mcm10− condition
SRR39748815	Total reads of Fen1/Cdc9+ and Mcm10− condition
SRR39748827	Total reads of WT and Mcm10+ condition
SRR39748821	Total reads of Pol δ− and Mcm10+ condition
SRR39748817	Total reads of Ctf4− and Mcm10+ condition
SRR39748819	Total reads of Csm3/Tof1− and Mcm10+ condition
SRR39748825	Total reads of Mrc1− and Mcm10+ condition
SRR39748814	Total reads of Dpb3/4− and Mcm10+ condition
SRR39748823	Total reads of Pol32− and Mcm10+ condition
SRR39748816	Total reads of Fen1/Cdc9+ and Mcm10+ condition
SRR39754383	Unbiotinylated reads of WT and Mcm10− condition
SRR39754377	Unbiotinylated reads of Pol δ− and Mcm10− condition
SRR39754357	Unbiotinylated reads of Ctf4− and Mcm10− condition
SRR39754361	Unbiotinylated reads of Csm3/Tof1− and Mcm10− condition
SRR39754381	Unbiotinylated reads of Mrc1− and Mcm10− condition
SRR39754355	Unbiotinylated reads of Dpb3/4− and Mcm10− condition
SRR39754379	Unbiotinylated reads of Pol32− and Mcm10− condition
SRR39754353	Unbiotinylated reads of Fen1/Cdc9+ and Mcm10− condition
SRR39754384	Unbiotinylated reads of WT and Mcm10+ condition
SRR39754378	Unbiotinylated reads of Pol δ− and Mcm10+ condition
SRR39754358	Unbiotinylated reads of Ctf4− and Mcm10+ condition
SRR39754372	Unbiotinylated reads of Csm3/Tof1− and Mcm10+ condition
SRR39754382	Unbiotinylated reads of Mrc1− and Mcm10+ condition
SRR39754356	Unbiotinylated reads of Dpb3/4− and Mcm10+ condition
SRR39754380	Unbiotinylated reads of Pol32− and Mcm10+ condition
SRR39754354	Unbiotinylated reads of Fen1/Cdc9+ and Mcm10+ condition
SRR39754375	Biotinylated reads of WT and Mcm10− condition
SRR39754359	Biotinylated reads of Pol δ− and Mcm10− condition

SRR39754370	Biotinylated reads of Ctf4− and Mcm10− condition
SRR39754373	Biotinylated reads of Csm3/Tof1− and Mcm10− condition
SRR39754364	Biotinylated reads of Mrc1− and Mcm10− condition
SRR39754368	Biotinylated reads of Dpb3/4− and Mcm10− condition
SRR39754362	Biotinylated reads of Pol32− and Mcm10− condition
SRR39754366	Biotinylated reads of Fen1/Cdc9+ and Mcm10− condition
SRR39754376	Biotinylated reads of WT and Mcm10+ condition
SRR39754360	Biotinylated reads of Pol δ− and Mcm10+ condition
SRR39754371	Biotinylated reads of Ctf4− and Mcm10+ condition
SRR39754374	Biotinylated reads of Csm3/Tof1− and Mcm10+ condition
SRR39754365	Biotinylated reads of Mrc1− and Mcm10+ condition
SRR39754369	Biotinylated reads of Dpb3/4− and Mcm10+ condition
SRR39754363	Biotinylated reads of Pol32− and Mcm10+ condition
SRR39754367	Biotinylated reads of Fen1/Cdc9+ and Mcm10+ condition

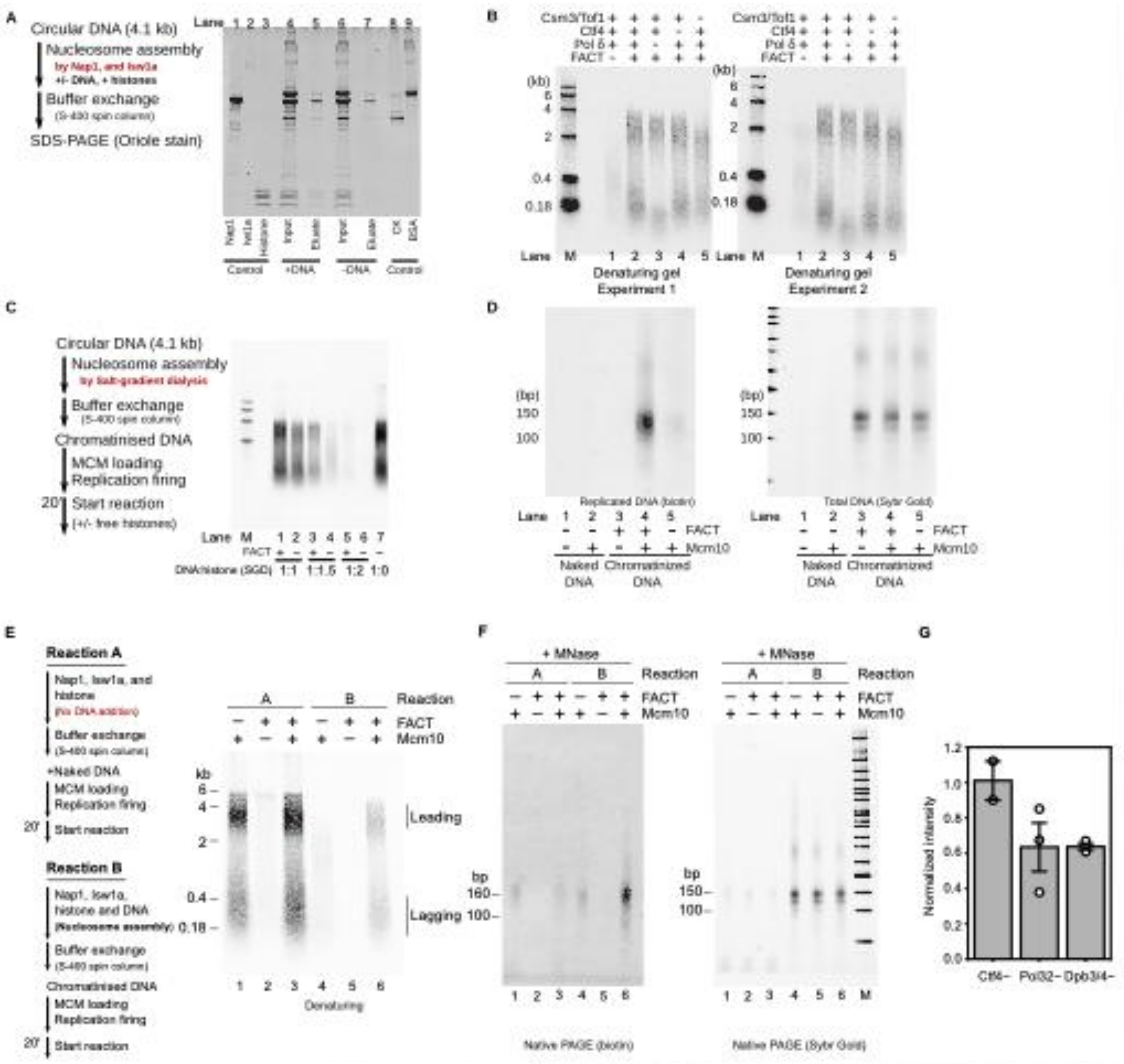

Supplementary Figure 1: *In vitro* reconstitutions of chromatin replication. **(A)** SDS–polyacrylamide gel analysis of denatured proteins in the nucleosome reconstitution reactions before (input) and after (eluate) buffer exchange using an S-400 spin column. We repeated twice giving the consistent result. **(B)** Alkaline denaturing agarose gels showing replicated DNA from two additional independent experiments using NI-chromatin as the substrate. 'M' denotes a marker (See the Methods). **(C)** Alkaline denaturing agarose gels showing replicated DNA using SGD-chromatin as the substrate. 'M' denotes a marker (See the Methods). We repeated twice giving the consistent result. **(D)** Native polyacrylamide gel analysis of total (right) and replicated (left) DNA after MNase digestion. We repeated twice giving the consistent result. **(E)** Alkaline denaturing agarose gels showing replicated DNA from reactions using naked DNA substrate with contaminating histones, Nap1, and ISW1a (Reaction A), and chromatinized DNA (Reaction B). We repeated twice giving the consistent result. **(F)** Native polyacrylamide gel analysis of total (right) and replicated (left) DNA after MNase digestion

of the reactions shown in (E). We repeated twice giving the consistent result. **(G)** Quantification of the ~150 bp biotinylated DNA fragments in the Ctf4−, Pol32−, and Dpb3/4− reactions, normalized to the corresponding signal intensity in the WT reaction. Error bars indicate mean ± standard error (n = 2 for Ctf4− and n = 3 for Pol32− and Dpb3/4− reactions). Source data are provided as a Source Data file.

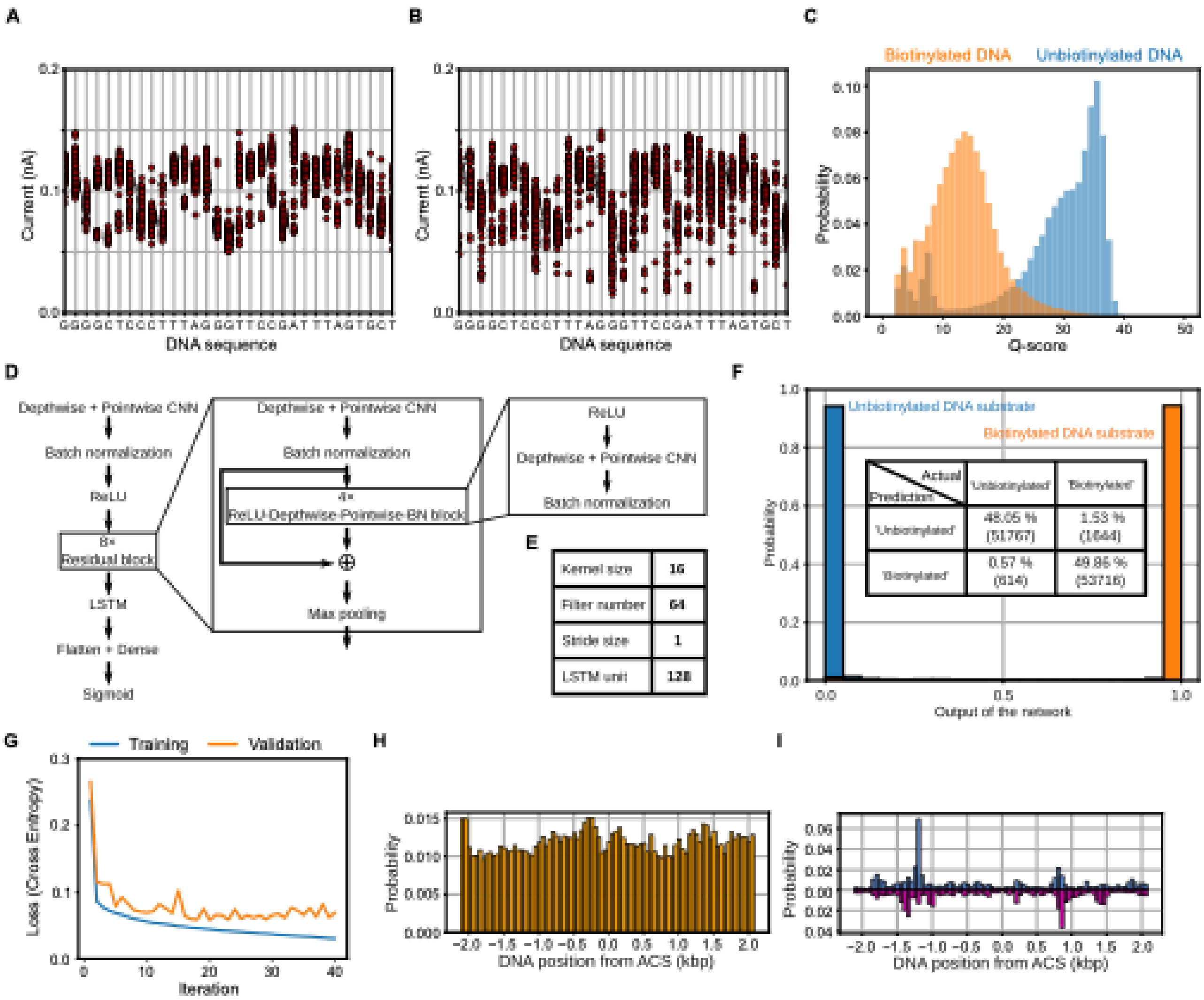

Supplementary Figure 2: Deep-learning model for detecting biotinylated DNA. **(A, B)** Current levels of unbiotinylated (A) and biotinylated (B) DNA aligned to the reference DNA sequence using the Adaptive Banded Event Alignment (ABEA) algorithm. Each dot represents the mean current level of a single read at each position. **(C)** Probability distributions of the basecalling quality score (Q-score) for unbiotinylated (blue) and biotinylated (orange) DNA. **(D)** Architecture of the neural network. **(E)** Parameters of the convolutional and LSTM layers. **(F)** Probability distributions of model outputs for the validation datasets. The inset table shows the confusion matrix. **(G)** Loss curves showing model performance evaluated on the training (blue) and validation (orange) datasets during the training process. **(H)** Position distribution of DNA fragments from naked plasmid DNA sheared by sonication. **(I)** Position distribution of histones on the lagging and leading strands in the Mcm10− reaction. Source data are provided as a Source Data file.

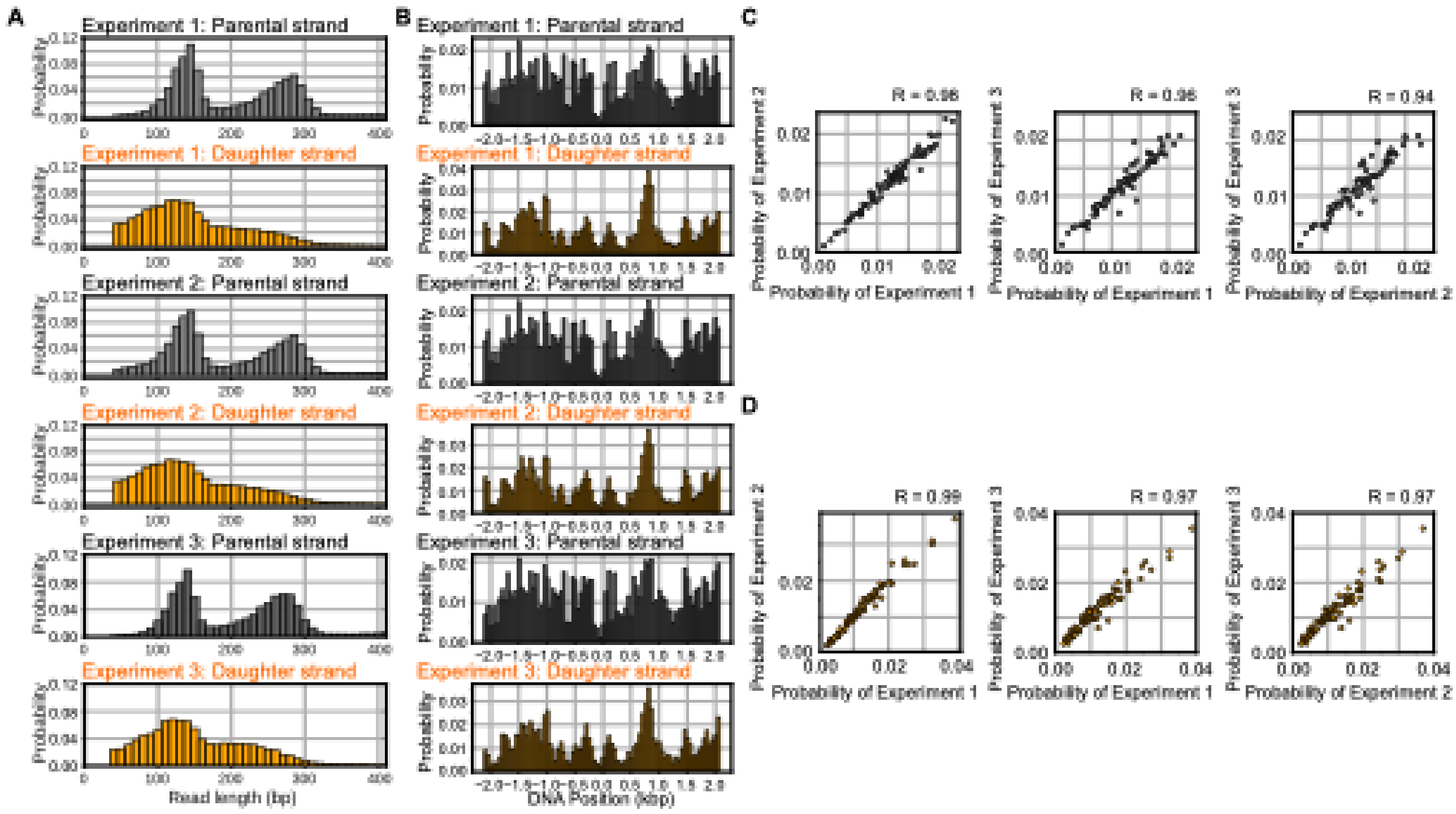

Supplementary Figure 3: Repli-pore-seq analysis of reconstituted chromatin replication. **(A)** Read-length distributions of parental DNA fragments (1st, 3rd, and 5th rows; gray) and daughter DNA fragments (2nd, 4th, and 6th rows; orange) from three independent experiments. **(B)** Position distributions of histones on parental DNA (1st, 3rd, and 5th rows; gray) and daughter DNA (2nd, 4th, and 6th rows; orange) from three independent experiments. **(C, D)** Correlation plots comparing the position distributions of histones on parental DNA (C) and daughter DNA (D) between two of the three experiments shown in (B). Source data are provided as a Source Data file.

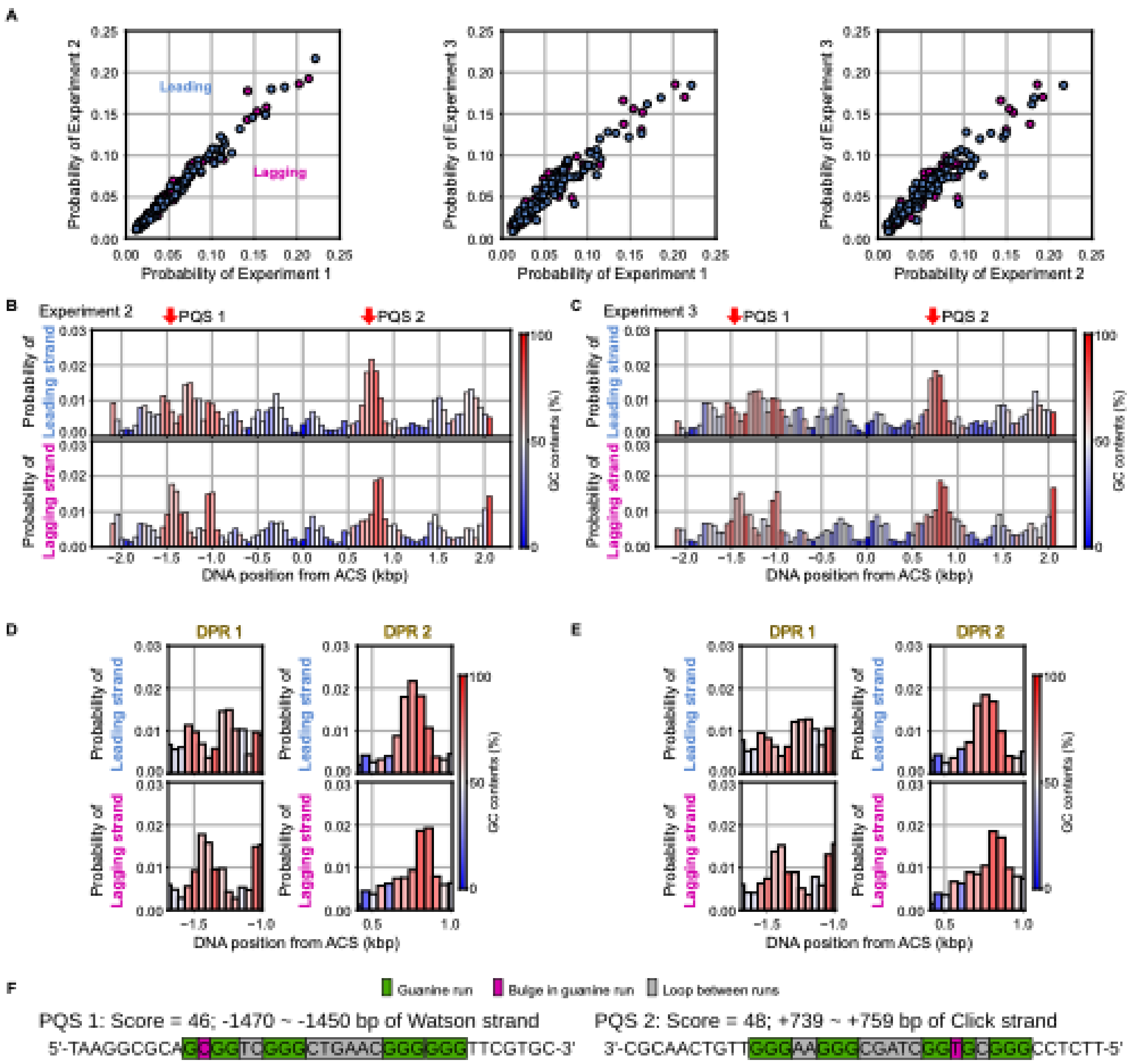

Supplementary Figure 4: Positions of recycled histones on the lagging and leading strands. **(A)** Correlation plot comparing the position distributions of recycled histones on the lagging (magenta) and leading (cyan) strands between two of the three independent experiments. **(B, C)** Position distributions of recycled histones on the leading (top) and lagging (bottom) strands for the second (B) and third (C) experiments. Colors indicate GC content. **(D, E)** Enlarged views of discordant positioning regions (DPRs) shown in Fig. 4E for the second (D) and third (E) experiments. Colors indicate GC content. **(F)** Sequences of the predicted PQS sites. Source data are provided as a Source Data file.

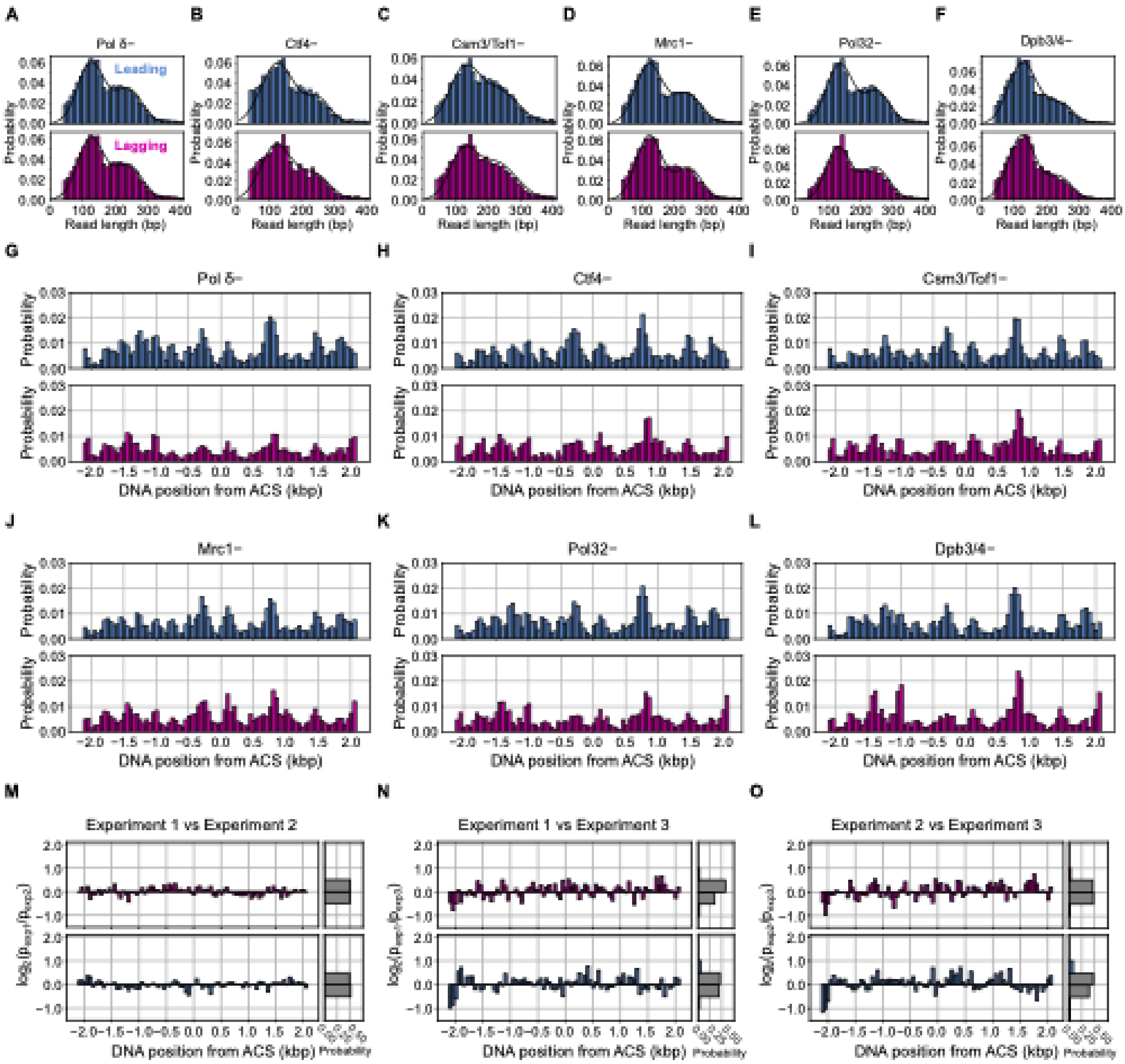

Supplementary Figure 5: Effects of Pol δ, Ctf4, Csm3/Tof1, Mrc1, Pol32, and Dpb3/4 depletion on histone recycling. **(A–F)** Read-length distributions of leading-strand (top, cyan) and lagging-strand (bottom, magenta) fragments in the Pol δ− and Pol δ+ reactions (A), Ctf4− and Ctf4+ reactions (B), Csm3/Tof1− and Csm3/Tof1+ reactions (C), Mrc1− and Mrc1+ reactions (D), Pol32− and Pol32+ reactions (E), and Dpb3/4− and Dpb3/4+ reactions (F). In all conditions, we repeated twice getting the consistent results. **(G–L)** Position distributions of recycled histones on the leading (top, cyan) and lagging (bottom, magenta) strands in the Pol δ− and Pol δ+ reactions (G), Ctf4− and Ctf4+ reactions (H), Csm3/Tof1− and Csm3/Tof1+ reactions (I), Mrc1− and Mrc1+ reactions (J), Pol32− and Pol32+ reactions (K), and Dpb3/4− and Dpb3/4+ reactions (L). In all conditions, we repeated twice getting the consistent results. **(M–O)** Logarithmic fold changes (log_2FC) in the position distributions of recycled histones on the leading (top) and lagging (bottom) strands calculated from all possible pairwise combinations of three independent experiments performed in the presence of all corresponding factors.

In the histograms, bars are colored gray for positions with $\log_2$FC < 0.5 and cyan (top) or magenta (bottom) for positions with $\log_2$FC $\geq$ 0.5. Source data are provided as a Source Data file.

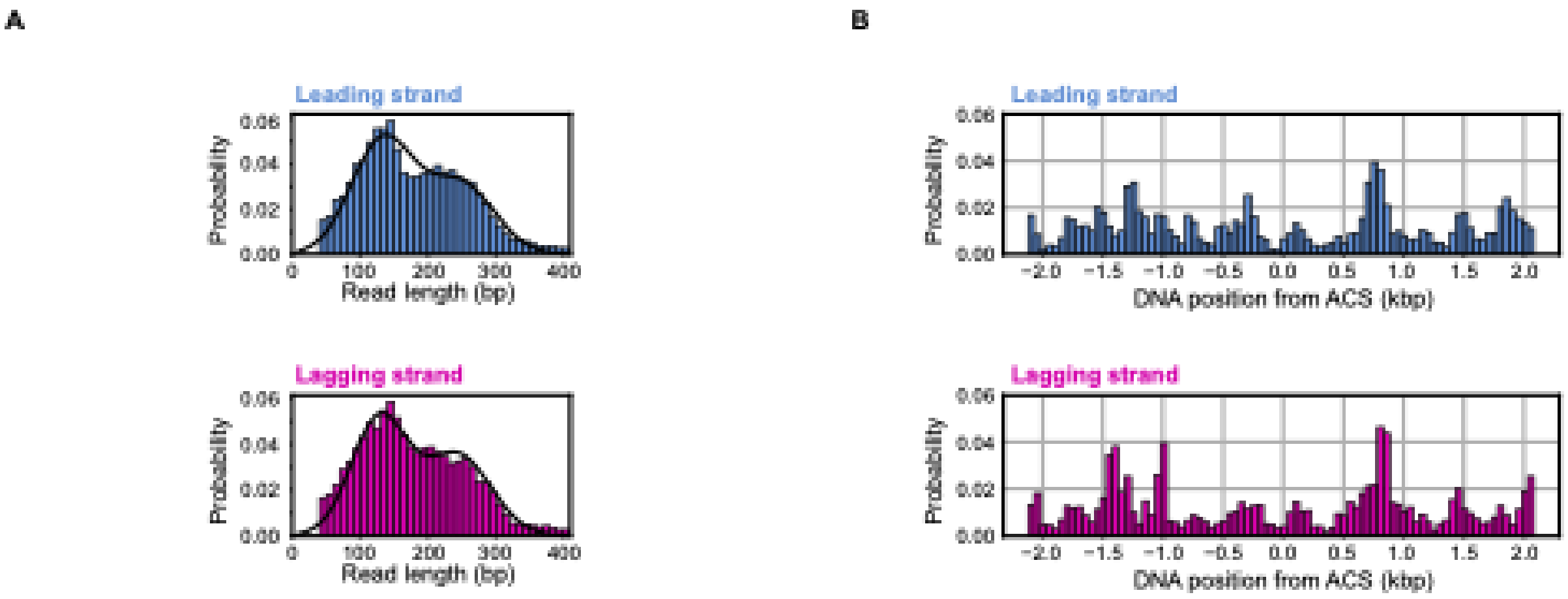

Supplementary Figure 6: Positions of recycled histones on the lagging and leading strands in Fen1/Cdc9+ reactions. (A) Read-length distributions of leading-strand (top, cyan) and lagging-strand (bottom, magenta) fragments. We repeated twice getting the consistent results. (B) Position distributions of recycled histones on the leading (top, cyan) and lagging (bottom, magenta) strands. We repeated twice getting the consistent results. Source data are provided as a Source Data file.
