# Supplementary material for: In vitro reconstitution of chromatin replication recapitulates symmetric histone recycling
Source: Nat Commun. 2026 Aug 11;17:9627. doi: 10.1038/s41467-026-76520-5 (PMC13554108; doi:10.1038/s41467-026-76520-5)
Supplement: Supplementary file 3 [file 41467_2026_76520_MOESM3_ESM.pdf]

nature portfolio

Peer Review File

In vitro reconstitution of chromatin replication recapitulates symmetric histone recycling

Corresponding Author: Professor Tsuyoshi Terakawa

This file contains all reviewer reports in order by version, followed by all author rebuttals in order by version.

Version 0:

Reviewer comments:

Reviewer #1

(Remarks to the Author)
Thank you for the opportunity to review your manuscript, which presents an innovative approach to investigating histone recycling during DNA replication. The authors introduce Repli-pore-seq, a deep learning-based method leveraging in vitro chromatin replication, nanopore sequencing, and neural network-based classification to analyze replication-coupled histone recycling. This study is innovative and addresses critical gaps in understanding epigenetic inheritance, offering valuable insights into chromatin replication dynamics.

However, several aspects require clarification and expansion to enhance the rigor, transparency, and broader applicability of the work. Specifically, I have concerns regarding data splitting, model justification, feature analysis, and validation strategies. Below, I outline several points that the authors may wish to address in a revision to strengthen your study.

- The manuscript would benefit from a supplementary descriptive analysis of the features (mean, SD, duration) used to train the model. For instance, scatterplots or density plots comparing mean vs. SD for biotinylated vs. unbiotinylated segments, and histograms of segment duration for both classes. These visualizations would help assess whether biotinylated and unbiotinylated DNA segments form clearly separable clusters or whether classification relies on complex, nonlinear relationships.

- The reported 98-99% accuracy is based on the full set of ~30 proteins, but it is unclear whether all proteins contribute equally to classification. An ablation study (removing one protein at a time) or a feature importance analysis (e.g., SHAP, permutation importance) could help assess the contribution of individual proteins. Additionally, a greedy selection approach could identify the minimal subset of proteins necessary to achieve high accuracy. This would simplify future experiments and improve interpretability of histone recycling mechanisms.

- While the deep neural network (DNN) achieves high accuracy, the rationale for choosing this architecture over simpler models (e.g., logistic regression, random forests) is unclear. Could the authors benchmark the DNN against these alternatives using the same features (mean/SD/duration)? Providing a performance comparison in a supplementary table would clarify whether deep learning offers a meaningful improvement over traditional models.

- The current training-validation split is performed randomly, which may introduce dependencies between training and validation data. In genetics, training and testing are often split across independent chromosome groups to prevent data leakage. In this study, a more independent split could involve partitioning by genomic sequence properties (e.g., AT-rich vs. GC-rich regions) or strand orientation (leading vs. lagging strand). This would help determine whether classification is driven by sequence-specific biases or differences in nanopore signal dynamics.

- To further strengthen the study's impact, it would be valuable to discuss the model's potential generalizability to other experimental systems (e.g., in vivo datasets or alternative nucleotide analogs like EdU/BrdU). A brief exploration of how signal variability or noise in external datasets might affect performance—or a discussion of strategies for model adaptation—could help readers assess its broader applicability. This would also highlight the pipeline's versatility for future epigenetic studies.

- The study elegantly leverages raw nanopore signals to detect biotin-dUTP modifications, bypassing traditional biochemical

purification. While this approach is well-justified, a future analysis incorporating basecalled sequences could reveal sequence-specific determinants of replication (e.g., motif enrichment in GC-rich discordant regions). Comparing nucleosome positions inferred from raw signals to sequence-based predictions could reveal whether observed recycling biases stem from biophysical constraints or specific DNA sequence motifs. While not essential for this study, this direction could further bridge the biochemical and genetic aspects of replication.

(Remarks on code availability)

Reviewer #2

(Remarks to the Author)

In this manuscript, Nagae et al reconstituted histone recycling in vitro using ~30 purified proteins and developed a new Repli-pore-seq pipeline to analysis the Micrococcal nuclease-digested replication products. They found that histones are symmetrically recycled to leading or lagging strands and found that the absent of Pol δ disrupted the histone recycling symmetry. In addition, they found that Fen 1 is not required for histone recycling. While these findings on Pol δ and Fen 1 were consistent with what have already reported in vivo, however, Pol δ is essential for DNA synthesis in vivo. Therefore, the conclusion on Pol δ in histone recycling without the presence of Pol δ in DNA synthesis is not reliable. To compound this issue, they found that the removal of Ctf4 or Csm3/Tof1 altered histone recycling position. These conclusions were in consistent with what have published from the in vivo study. Therefore, this in vivo system needs to be further tested and validated. Otherwise, the conclusions generated from this study will mislead the field.

First, the in vitro system should be further validated. First, the authors omitted Pol δ from the reaction to conclude that Pol δ is needed for parental histone recycling. In cells, Pol δ is essential for DNA synthesis. Furthermore, its omission also impairs lagging-strand synthesis. Differences in leading and lagging strands synthesis in the absence of Pol δ explain the asymmetry rather than a direct role of Pol δ in histone positioning? Therefore, to support this conclusion, the authors should repeat the experiments using a Pol δ mutant that cannot bind histones. At minimum, the authors should repeat the experiments using Pol δ mutant complex lacking Pol32, which is not essential for DNA synthesis.

Second, because of conflicting results on Ctf4 and Csm3/Tof1, the authors should test the effects of Mcm2 and Pol1 mutant that cannot bind histones in their system. Furthermore, the authors should test the effect of Mrc1 in their system. Without testing the in vitro system through these means, it is challenging to interpret the conflicting results generated from this study.

Third, the deep-learning model for biotinylated DNA classification is a key component of this study. However, its implementation and validation require further clarification:
a. The distribution of nanopore signal events for biotinylated vs. unbiotinylated DNA is not presented.
b. A visualization of raw signals (before and after classification) would help assess model reliability.
c. The model was trained on PCR-amplified biotinylated DNA, how similar is this dataset to biotinylated DNA from reconstituted chromatin replication? A direct comparison of signal features between these datasets would be needed.
d. Were both GC-rich and AT-rich sequences included in the training data? If PCR-amplified DNA is AT-rich, the classifier may perform poorly on GC-enriched regions in chromatinized DNA. The 98% accuracy is impressive, but potential sequence-dependent biases (e.g., GC-rich regions) are not explored.
e. How was the biotin-dUTP concentration determined? Could higher biotin concentrations artificially inflate the fraction of detected biotinylated reads (e.g., from 11%)?
f. No loss curve is provided to assess overfitting during training.
g. assessment of the impact of biotinylation on basecaller performance (e.g., Dorado) is needed.
h. What is the unit of the 600-segment feature vector? If segments correspond to base pairs (bp), why was 600 chosen instead of 400, given that Figure 4B shows read lengths in the 50–500 bp range? Figures 3B and 4B suggest a 50 bp cutoff for read filtering. Was this a hard threshold, and if so, what is its impact on the analysis?

Minor concerns:

1. Figure 1B and the third panel of Supplementary Figure 1B are same figures.

2. In Supplementary Figure 1B, why is the histone band in lane 7 weaker than in lane 5? If histones bind to DNA, the histone band in the eluate (+DNA, lane 5) should be weaker than in the eluate (-DNA, lane 7) due to reduced free histone availability. Could the authors explain this result?

3. In Supplementary Figure 1C, why is the band signal in lane 7 stronger than in lane 2? Furthermore, why are the band signals in lanes 7–9 for the DNA:histone = 1:0 condition stronger than other lanes for DNA:histone = 1:1-1:2 if the histone ratio is 0?
4. Fig. 1E-F. The authors wrote: "Inspection of replicated (biotinylated) DNA after the MNase digestion confirmed nucleosome deposition after replication in the absence of Ctf4, Csm3-Tof1, or Pol δ, in the same way as their presence (Fig. 1E, lanes 2, 4, and 6 & 1F, lanes 2, 4, 6, 8, and 10). These results indicated that Pol δ, Ctf4, and Csm3/Tof1 are dispensable for histone deposition to replicated DNA". This conclusion contradicted the results shown. The authors should quantify replicated DNA in the presence and absence of each factor before Mnase digestion. The authors then need to quantify replicated after Mnase digestion.

5. Fig. 4. The authors wrote: “we observed the discordance of the recycled position between leading and lagging strands on the GC-rich DNA sequences”. This statement is misleading. As the authors noted that the discordance may be because of GC rich DNA sequences on DNA synthesis, not necessarily on recycled histone positions.
6. As authors stated, some nucleosomes lost one or two H2A/H2B dimers to form hexasomes or tetrasomes after histone recycling. Could the authors perform other experiments to further confirm this conclusion?

7. In Figure 3B, why do the parental DNA fragments exhibit two peaks? Does this indicate that the recycled histones on parental DNA consist of a mix of mono- and di-nucleosomes?

8. Since The authors mentioned eSPAN multiple times, the following papers should be cited PMCID: PMC4362665, PMCID: PMC7449674.
9. Since the authors cited Reference Mrc1’s role in parental histone recycling, the following reference should be cited. PMCID: PMC11414769

(Remarks on code availability)

Reviewer #3

(Remarks to the Author)
Reviewer Comments for Nature Communications 576405

The manuscript, "Minimal protein list for chromatin replication-coupled symmetric histone recycling revealed through in vitro reconstitution," by Nagae and colleagues, explores the mechanism of histone recycling during chromatin replication using an elegant and sophisticated in vitro system. Additionally, the authors introduce a new bioinformatic pipeline that employs deep learning and nanopore sequencing to identify biotinylated DNA, which may become a valuable tool in the field. Altogether, this study addresses an important question in the field - identifying the replisome components necessary for promoting histone recycling symmetry, and accuracy during DNA replication.

The manuscript provides some valuable insights into the roles of Polymerase delta and Ctf4/Tof1 in recycling symmetry and accuracy, respectively, and offers initial evidence of how DNA sequence may influence histone recycling accuracy. However, the benchmarking the accuracy and sensitivity of this in vitro approach against available in vivo data/mutants remains largely limited. To address this and underscore the applicability of the in vitro replication assay, the authors should include additional controls to better estimate the degree of asymmetry detectable in vitro. Moreover, while the system developed is powerful, the study lacks some depth in addressing biological questions. Therefore, the authors should evaluate the impact of more recently discovered histone-binding mutants (potentially in combination, e.g. a leading and lagging strand recycling mutant) and/or test replication stress-inducing drugs on chromatin replication symmetry and accuracy before the manuscript can be considered for publication.

Major Comments

1. The study focuses on Polymerase delta’s role in lagging strand recycling, but additional controls are necessary. The authors should introduce mutations in Mcm2, or omit Polymerase epsilon subunits 3 and 4 (Pole3/4, or Dpb3/4 in yeast) as well as Polymerase delta subunit Pold32 to better recapitulate the in vivo effects in their in vitro system. Ideally, combinations of these mutants (disabling leading and lagging strand recycling) would also reveal to what extent histones may be lost during DNA replication and to what an “intrinsic” affinity of parental histones to newly synthesized DNA may compensate for disabled histone recycling.

2. A third biological replicate for histone recycling accuracy and positioning on the leading vs. lagging strand would significantly enhance the statistical robustness of the study.

3. The NI-system is complex and requires further clarification, in particular regarding the de novo H2A-H2B (and H3-H4) deposition activity by Nap1 on replicated chromatin. The authors should exclude the possibility that Nap1 deposits histones independently of DNA replication?

4. A 20× discrimination between true and false positives may not be sufficient to resolve the subtle differences observed in Polymerase delta mutants, especially considering potential DNA synthesis reduction, which could further skew the ratio of true to false positives.

5. Given that H2A-H2B are proposed to be also recycled, does the tetramer/hexamer ratio change if Nap1 is omitted from the reaction? If so, this would suggest that Nap1 deposits H2A-H2B independently to form hexamers/octamers on replicated chromatin rather than through histone recycling.

Minor Comments

6. Please provide a full list of proteins used in the study, especially listing the subunits of the Polymerases used.

7. Clarify the meaning of LSTM abbreviation.

8. The axis label of Figure 4 is a bit confusing. How can probability vary between 0 to 1 (Figure 4D) and be negative or slightly positive (remaining panels in Figure 4)? Do the authors refer to the relative probabilities vs absolute probability? Please elaborate.

9. To complement histone recycling mutants, the authors could also use replication stress-inducing drugs and assess their effects on histone recycling accuracy in vitro. This would provide valuable insights that may be difficult to visualize in vivo due to pleiotropic effects of these drugs (e.g. checkpoint signaling)

(Remarks on code availability)

Version 1:

Reviewer comments:

Reviewer #1

(Remarks to the Author)
The authors have addressed all of my concerns thoroughly by providing the requested benchmarks, visualizations, and clarifications. I am satisfied with the added rigor regarding model justification and data splitting, and I have no further concerns.

(Remarks on code availability)

Reviewer #2

(Remarks to the Author)
In the revised manuscript, the authors tested the effects of mission of several components (Pol32, Ctf4, Dpb3/Dpb4, Mrc1) known to be involved in parental histone recycling/transfer. While these results largely strengthened the manuscript, the results shown largely confirmed what is known about parental histone transfer. Therefore, the authors need to highlight new insights into this process gained from this study. In addition, I have additional concerns and suggestions based on new results presented.

1) “Moreover, certain PTMs, which have been used as markers of old histones, can be added to newly synthesized histones by histone-modifying enzymes6,10. These enzymes, which alter the epigenetic states of nucleosomes, complicate the interpretation of genome-wide sequencing studies”. The statement is confusing. The restoration of modifications on new histones is an integral part of chromatin replication process.

2) Fig. 1E-H. The relative amount of newly synthesized nucleosomal fragments in reactions lacking Ctf4, Pol32 and Dpb3-Dpb4 should be quantified and see whether each omission affect nucleosome assembly and/or DNA replication.

3) Fig. 5H-M. Based on these results, the authors proposed that removal of Ctf4, Csm3/Tof1, or Mrc1 shifts recycled histones toward energetically less favorable positions and that these factors coordinate DNA sequence–dependent positioning of recycled histones (Fig. 5H to 5M). These results and conclusion do not make sense. How can proteins involved in DNA replication, which are sequence independent, confer sequence-dependent nucleosome position? How accurate/confident are for the estimation of nucleosome positions using this approach? Since the chromatin is digested with MNase, the authors should use short read sequencing to map nucleosome position as an independent approach to confirm the results/conclusion. Also what is the advantage to use long-read sequencing compared to short-read sequencing to map nucleosome position?

4) Fig. 6. In vivo study indicate that Fen1 deletion does not affect parental histone transfer bias, which is in consistent with in vitro system. Did the authors perform experiments without Fen1 alone?

(Remarks on code availability)

Reviewer #3

(Remarks to the Author)
The authors have provided a substantially improved manuscript and added additional, insightful experiments that address most of this reviewer’s comments. They provide further controls using a number of in vivo derived histone recycling mutants and can recapitulate their effects in vitro. Also, they invested more time in clarity, completeness and important details throughout the whole manuscript.

One small thing remains from this reviewer's side: It should be still possible to analyse the recycling accuracy and symmetry in double mutants that do not affect DNA synthesis (e.g. the Pold32 and Dpb3/4), as in their in vitro system, one does not need to generate elaborate double mutants but rather just omit complex subunits from their mix (e.g. omit Pold32, Dpb3, Dpb4). Looking at the effect of reduced histone recycling to both daughter strand would bring in additional novelty to this manuscript.

(Remarks on code availability)

Version 2:

Reviewer comments:

Reviewer #2

(Remarks to the Author)
The authors addressed some concerns, but not two concerns described below.

1) The following statement is not accurate. “Although omission of Pol32 and Dpb3/4 moderately decreased the levels of newly synthesized nucleosomal fragments compared with reactions containing these factors (Supplementary Fig. 1G), substantial nucleosome deposition on replicated DNA was still observed, indicating that these factors are not strictly required for parental histone deposition itself.”

Substantial nucleosome deposition on replicated DNA was still observed during omission of Pol32 and Dpb3/Dpb4 do not mean that these proteins are not strictly required for parental histone deposition. Instead, the data indicate that other factors may function in parental histone transfer in their absence.

Original Concern #4). Fig. 6. In vivo study indicate that Fen1 deletion does not affect parental histone transfer bias, which is in consistent with in vitro system. Did the authors perform experiments without Fen1 alone? The reference describing the effects of Fen1 deletion and DNA2 depletion on parental histone transfer is PMID: 38838138

(Remarks on code availability)

Open Access This Peer Review File is licensed under a Creative Commons Attribution 4.0 International License, which

permits use, sharing, adaptation, distribution and reproduction in any medium or format, as long as you give appropriate credit to the original author(s) and the source, provide a link to the Creative Commons license, and indicate if changes were made.
In cases where reviewers are anonymous, credit should be given to 'Anonymous Referee' and the source.
The images or other third party material in this Peer Review File are included in the article's Creative Commons license, unless indicated otherwise in a credit line to the material. If material is not included in the article's Creative Commons license and your intended use is not permitted by statutory regulation or exceeds the permitted use, you will need to obtain permission directly from the copyright holder.
To view a copy of this license, visit https://creativecommons.org/licenses/by/4.0/

Responses to Reviewer #1

Comment 1: Thank you for the opportunity to review your manuscript, which presents an innovative approach to investigating histone recycling during DNA replication. The authors introduce Repli-pore-seq, a deep learning-based method leveraging in vitro chromatin replication, nanopore sequencing, and neural network-based classification to analyze replication-coupled histone recycling. This study is innovative and addresses critical gaps in understanding epigenetic inheritance, offering valuable insights into chromatin replication dynamics. However, several aspects require clarification and expansion to enhance the rigor, transparency, and broader applicability of the work. Specifically, I have concerns regarding data splitting, model justification, feature analysis, and validation strategies. Below, I outline several points that the authors may wish to address in a revision to strengthen your study.

Response 1: We thank Reviewer #1 for the positive assessment of our study and for the suggestions to improve rigor. In the revised manuscript, we addressed all concerns regarding (i) data splitting and potential data leakage, (ii) justification for selecting a deep-learning architecture over simpler models, (iii) additional analyses supporting feasibility of classification, and (iv) validation and limitations, including discussion of generalizability to other nucleotide analogs. Below, we provide point-by-point responses and indicate all changes in the revised manuscript.

Comment 2: The manuscript would benefit from a supplementary descriptive analysis of the features (mean, SD, duration) used to train the model. For instance, scatterplots or density plots comparing mean vs. SD for biotinylated vs. unbiotinylated segments, and histograms of segment duration for both classes. These visualizations would help assess whether biotinylated and unbiotinylated DNA segments form clearly separable clusters or whether classification relies on complex, nonlinear relationships.

Response 2: We agree that additional analyses of the signal features would help assess the feasibility of the classification. To address this point, we added supplementary visualizations comparing raw current signals between biotinylated and unbiotinylated DNA after alignment to the reference sequence using the ABEA

algorithm (Supplementary Fig. 2A & 2B). These analyses demonstrate that incorporation of biotin-dUTP produces reproducible perturbations in current signals, supporting the separability of the two classes at the signal level. Consistent with this, we further show that simpler classifiers relying on linear or shallow decision boundaries perform poorly, indicating that classification relies on nonlinear relationships among signal features rather than simple thresholding; this point is further addressed in our response to Comment 4.

To clarify these points, we added the Supplementary Figure 2A & 2B and the sentence, 'To visualize how incorporation of biotin-dUTP perturbs nanopore current signals, we aligned raw current traces to the reference DNA sequence using the Adaptive Banded Event Alignment (ABEA) algorithm (Supplementary Fig. 2A & 2B). This analysis revealed that biotinylated nucleotides exhibit characteristic current patterns that are distinct from those of the four canonical nucleotides, consistent with previous reports.' to the Result section.

Comment 3: The reported 98-99% accuracy is based on the full set of ~30 proteins, but it is unclear whether all proteins contribute equally to classification. An ablation study (removing one protein at a time) or a feature importance analysis (e.g., SHAP, permutation importance) could help assess the contribution of individual proteins. Additionally, a greedy selection approach could identify the minimal subset of proteins necessary to achieve high accuracy. This would simplify future experiments and improve interpretability of histone recycling mechanisms.

Response 3: We appreciate the reviewer's suggestion to assess the contribution of individual proteins to classification performance. However, in our study, the reported 98–99% classification accuracy does not depend on the presence or absence of replication proteins in the reaction. The deep-learning classifier was trained exclusively on nanopore current signals obtained from purified PCR-amplified DNA substrates, and all proteins were removed prior to library preparation for sequencing. Therefore, protein components in the replication reactions do not contribute to, nor influence, the classification task.

To clarify this point, we added the sentence, 'Because the same DNA sequence was used for both the replication assays and classifier training, and all proteins were removed prior to library preparation, the classification relies solely on biotin-dUTP incorporation into DNA fragments.' to the Results section.

Comment 4: While the deep neural network (DNN) achieves high accuracy, the rationale for choosing this architecture over simpler models (e.g., logistic regression, random forests) is unclear. Could the authors benchmark the DNN against these alternatives using the same features (mean/SD/duration)? Providing a performance comparison in a supplementary table would clarify whether deep learning offers a meaningful improvement over traditional models.

Response 4: We agree that justification of the deep-learning architecture requires comparison with simpler models. To address this point, we benchmarked our deep neural network against logistic regression and random forest classifiers trained on the same input features (segment mean, standard deviation, and duration). While the DNN achieved an accuracy of 98–99%, logistic regression and random forest models reached only 65% and 67% accuracy, respectively. These results indicate that the classification task cannot be adequately solved by linear or shallow decision boundaries and instead requires nonlinear integration of signal features.

To clarify this point, we added a sentence, 'The accuracies of simpler classification models, such as logistic regression and random forests, were 65% and 67%, respectively, indicating that the classification task in the present study requires the expressive power of a deep neural network.' to the Results section.

Comment 5: The current training-validation split is performed randomly, which may introduce dependencies between training and validation data. In genetics, training and testing are often split across independent chromosome groups to prevent data leakage. In this study, a more independent split could involve partitioning by genomic sequence properties (e.g., AT-rich vs. GC-rich regions) or strand orientation (leading vs. lagging strand). This would help determine whether classification is driven by sequence-specific biases or differences in nanopore signal dynamics.

Response 5: We appreciate the reviewer's concern regarding potential dependencies introduced by random splitting of training and validation data. In our system, both the training dataset and the experimental Repli-pore-seq data derive from the same defined DNA sequence. Therefore, partitioning the data by sequence properties (e.g., AT-rich vs. GC-rich regions) or by strand orientation would artificially separate specific sequence contexts between training and validation

sets and could bias the classifier toward sequence-dependent features rather than biotin-dependent signal perturbation. To ensure that the classifier generalizes across the full range of sequence contexts present in the experiment, we used random splitting so that sequence properties are well balanced between the training and validation datasets. We confirmed that AT content (53.2 ± 3.1% and 53.2 ± 3.1%) and strand orientation (46.7% and 46.9%) were nearly identical between the two sets, indicating that classification performance is not driven by sequence-specific biases but by nanopore signal features associated with biotin-dUTP incorporation.

To clarify this point, we modified the sentences, 'Because both the training data and the experimental Repli-pore-seq data derive from the same defined DNA sequence, splitting the dataset by sequence properties (such as GC content or strand orientation) would artificially segregate specific sequence contexts between the training and validation sets. We therefore adopted random splitting to ensure equivalent representation of sequence features across both datasets. Consistent with this design, AT content and strand orientation were nearly identical between the training and validation sets (53.2 ± 3.1% AT content and 46.7% vs. 46.9% Watson-strand reads), indicating that classification performance is not driven by sequence-specific bias.' in the Results section.

Comment 6: To further strengthen the study's impact, it would be valuable to discuss the model's potential generalizability to other experimental systems (e.g., in vivo datasets or alternative nucleotide analogs like EdU/BrdU). A brief exploration of how signal variability or noise in external datasets might affect performance—or a discussion of strategies for model adaptation—could help readers assess its broader applicability. This would also highlight the pipeline's versatility for future epigenetic studies

---

Response 6: We agree that discussion of the model's generalizability is important to highlight the broader applicability of the Repli-pore-seq pipeline. Previous studies [e.g. Georgieva et al. (2020) *Nucleic Acids Research* 48:e88] have demonstrated that incorporation of analogs such as BrdU and EdU also induces characteristic perturbations in nanopore current signals, supporting the feasibility of adapting our classification strategy beyond biotin-dUTP–based *in vitro* assays. At the same time, we note that the classifier in the present study was trained using a defined DNA sequence specific to this experimental system. Therefore,

application to other DNA sequences or experimental contexts would require retraining or fine-tuning of the model using appropriate training data.

To clarify this points, we added the sentence, 'Furthermore, previous studies have shown that incorporation of nucleotide analogs such as BrdU and EdU also induces characteristic perturbations in nanopore current signals and enables reliable detection of nascent DNA. These findings support the notion that the computational purification strategy developed here is, in principle, transferable to other experimental systems employing distinct nucleotide analogs. At the same time, because the classifier in the present study was trained on a defined DNA sequence and a specific analog (biotin-dUTP), application to different sequence contexts or analogs would require fine-tuning of the model using appropriate training data.' to the Discussion section.

Comment 7: The study elegantly leverages raw nanopore signals to detect biotin-dUTP modifications, bypassing traditional biochemical purification. While this approach is well-justified, a future analysis incorporating basecalled sequences could reveal sequence-specific determinants of replication (e.g., motif enrichment in GC-rich discordant regions). Comparing nucleosome positions inferred from raw signals to sequence-based predictions could reveal whether observed recycling biases stem from biophysical constraints or specific DNA sequence motifs. While not essential for this study, this direction could further bridge the biochemical and genetic aspects of replication.

Response 7: We would like to clarify that classification and sequence mapping are conceptually distinct steps in our pipeline. The classification of biotinylated versus unbiotinylated DNA fragments is performed exclusively using raw nanopore current signals and does not rely on basecalled sequence information. In contrast, basecalled sequences are used after classification to map reads onto the plasmid reference sequence for downstream analyses.

To clarify this point, we added the sentence, 'Importantly, classification of biotinylated reads is performed solely on raw nanopore current signals, whereas base-called sequence information is used after classification for sequence mapping and downstream positional analyses.' to the Discussion section.

Notably, incorporation of basecalled sequence information for downstream analyses has already provided biologically meaningful insights. As shown in Fig. 3E & 3F, a logarithmic distribution of recycled histone was correlated with free

energy landscape of nucleosome positioning predicted by primary DNA sequence. Moreover, we found that reproducible discordance between lagging- and leading-strand nucleosome positions preferentially occurs in regions enriched for GC content and predicted G-quadruplex–forming sequences, suggesting a link between local DNA secondary-structure propensity and strand-specific recycling behavior.

To extend the discussion, we added the sentence, 'Future Repli-pore-seq analyses using DNA substrates with distinct GC contents would further test this hypothesis. In addition, biophysical approaches that directly visualize replication dynamics, such as single-molecule fluorescence imaging and molecular dynamics simulations, could provide complementary means to evaluate the underlying mechanisms.' to the Discussion section.

Responses to Reviewer #2

Comment 1: In this manuscript, Nagae et al reconstituted histone recycling in vitro using ~30 purified proteins and developed a new Repli-pore-seq pipeline to analysis the Micrococcal nuclease-digested replication products. They found that histones are symmetrically recycled to leading or lagging strands and found that the absent of Pol δ disrupted the histone recycling symmetry. In addition, they found that Fen 1 is not required for histone recycling. While these findings on Pol δ and Fen 1 were consistent with what have already reported in vivo, however, Pol δ is essential for DNA synthesis in vivo. Therefore, the conclusion on Pol δ in histone recycling without the presence of Pol δ in DNA synthesis is not reliable. To compound this issue, they found that the removal of Ctf4 or Csm3/Tof1 altered histone recycling position. These conclusions were in consistent with what have published from the in vivo study. Therefore, this in vivo system needs to be further tested and validated. Otherwise, the conclusions generated from this study will mislead the field.

Response 1: We sincerely thank Reviewer 2 for the careful and critical evaluation of our manuscript. We appreciate the reviewer's concerns regarding the interpretation of Pol δ-dependent histone recycling, the apparent discrepancy with *in vivo* essentiality of Pol δ, and the seemingly contradictory effects observed for Ctf4 and Csm3/Tof1.

In response, we substantially revised the manuscript to clarify the scope and limitations of our *in vitro* reconstitution system and to strengthen the biological interpretation. Specifically, we (i) explicitly distinguished mechanistic perturbations *in vitro* from physiological states *in vivo*, (ii) performed additional Repli-pore-seq analyses using Pol32− (Pol δ mutant) and Dpb3/4− (Pol ε mutant) to disentangle the effects of impaired lagging-strand synthesis from direct histone-binding functions of DNA polymerases, and (iii) introduced rigorous statistical analyses to quantitatively assess strand bias and positional changes in histone recycling. These additions demonstrate that the observed effects are consistent with, rather than contradictory to, previously reported *in vivo* results.

As a result, guided by the reviewer's insightful comments, the revised manuscript resolves the apparent inconsistencies and presents a more coherent and robust framework for interpreting histone recycling mechanisms using *in vitro* reconstitution.

Comment 2: First, the in vitro system should be further validated. First, the authors omitted Pol δ from the reaction to conclude that Pol δ is needed for parental histone recycling. In cells, Pol δ is essential for DNA synthesis. Furthermore, its omission also impairs lagging-strand synthesis. Differences in leading and lagging strands synthesis in the absence of Pol δ explain the asymmetry rather than a direct role of Pol δ in histone positioning? Therefore, to support this conclusion, the authors should repeat the experiments using a Pol δ mutant that cannot bind histones. At minimum, the authors should repeat the experiments using Pol δ mutant complex lacking Pol32, which is not essential for DNA synthesis.

Response 2: We agree with the reviewer that deletion of Pol δ alone cannot distinguish whether the observed asymmetry in histone recycling arises from impaired lagging-strand synthesis or from a direct histone-binding function of Pol δ. We also acknowledge that complete loss of Pol δ represents a non-physiological condition *in vivo*, as Pol δ is essential for DNA replication and repair. The rationale for using Pol δ-depleted reactions in our *in vitro* system was to decouple mechanistic contributions to histone recycling from cellular viability constraints. In this defined setting, removal of essential factors provides insight into their specific roles in histone recycling, even when such perturbations are not viable in cells.

To directly address the reviewer's concern and to disentangle the effects of lagging-strand synthesis from histone binding, we performed additional Repli-pore-seq analyses using a Pol δ mutant lacking the non-essential Pol32 subunit, which mediates histone binding but does not abolish DNA synthesis. In the Pol32− mutant, histone recycling exhibited a leading-strand bias (57.8% in two biological replicates) that was intermediate between wild-type reactions (52.5% in three biological replicates) and Pol δ-depleted reactions (62.9% in two biological replicates). These results demonstrate that both efficient lagging-strand synthesis by Pol δ and direct histone binding via Pol32 contribute to symmetric histone recycling.

Guided by the reviewer's suggestion, we expanded the Results section by adding a new paragraph beginning with "Next, we investigated the effects of Pol δ, Ctf4, Csm3/Tof1, Mrc1−, Pol32−, and Dpb3/4− on the recycling symmetry." in which we systematically analyzed strand-specific recycling under each

condition (Fig. 5A). This newly added analysis is accompanied by formal statistical testing and demonstrates that the observed strand biases are consistent with previous *in vivo* studies using pol32Δ and other replication mutants in *Saccharomyces cerevisiae*. Moreover, the more pronounced bias observed upon complete Pol δ removal compared to the Pol32− mutant further supports the conclusion that balanced lagging- and leading-strand synthesis is required for symmetric histone recycling.

Comment 3: Second, because of conflicting results on Ctf4 and Csm3/Tof1, the authors should test the effects of Mcm2 and Pol1 mutant that cannot bind histones in their system. Furthermore, the authors should test the effect of Mrc1 in their system. Without testing the in vitro system through these means, it is challenging to interpret the conflicting results generated from this study.

Response 3: We thank Reviewer 2 for raising the concern regarding the seemingly conflicting effects of Ctf4 and Csm3/Tof1 and for suggesting additional analyses to clarify their roles in histone recycling. We acknowledge that, in the original version of the manuscript, conclusions regarding recycling symmetry were presented without formal statistical testing, which may have caused confusion in interpreting these results.

To address this issue, we performed chi-squared tests to quantitatively evaluate strand-specific recycling under each condition by comparing the frequencies of histone recycling to the leading strand. This analysis revealed that omission of Csm3/Tof1 and Ctf4 resulted in statistically significant increases in leading-strand recycling ($P = 2.8 \times 10^{-3}$ and 7.4×10^{-10}, respectively). Importantly, we also analyzed additional conditions, including Pol δ−, Pol32− (Pol δ mutant), Mrc1−, and Dpb3/4− (Pol ε mutant) reactions; analysis of Mcm2 histone-binding mutants was not feasible due to technical difficulties in obtaining a stable expression strain. These analyses showed that Pol δ− and Pol32− significantly increased leading-strand recycling, whereas Mrc1− and Dpb3/4− significantly reduced it, fully consistent with previous *in vivo* studies using pol32Δ, dpb3/4Δ, and mrc1Δ mutants in *Saccharomyces cerevisiae* [Tian et al. (2024) *Proc. Acad. Natl. Sci.*, 121 (**20**): e2400610121; Serra-Cardona et al. (2024) *Sci. Adv.*, 10 (**23**): eadn5175; Yu et al. (2024) *Cell*, 187 (**18**): 5010–5028; Yu et al. (2018) *Science*, 361 (**6409**): 1386–1389]. Notably, we directly tested the effect of Mrc1 in our *in vitro* system, as suggested by the reviewer, and found

that its removal caused a significant lagging-strand bias, in agreement with a cellular study using budding yeast [Yu et al. (2024) *Cell*, 187 (**18**): 5010–5028]. Together, these statistically supported analyses demonstrate that the effects of Ctf4 and Csm3/Tof1 are not contradictory [Gan et al. (2018) *Molecular Cell*, 72 (**1**): 140–151, Li et al. (2024) *Nature*, 627 (**8005**): 890–897] but instead reflect their contributions within a network of partially redundant histone-recycling pathways.

We have revised the Results section to explicitly incorporate these statistical analyses and clarify the interpretation, as described in the newly added paragraph beginning with "Next, we investigated the effects of Pol δ, Ctf4, Csm3/Tof1, Mrc1−, Pol32−, and Dpb3/4− on the recycling symmetry."

Comment 4: Third, the deep-learning model for biotinylated DNA classification is a key component of this study. However, its implementation and validation require further clarification: (a) The distribution of nanopore signal events for biotinylated vs. unbiotinylated DNA is not presented. A visualization of raw signals (before and after classification) would help assess model reliability.

Response 4: We appreciate the reviewer's request—also raised by Reviewer 1—to clarify the signal-level differences between biotinylated and unbiotinylated DNA. To address this point, we added supplementary analyses visualizing nanopore current signal distributions after alignment to a reference DNA sequence using the Adaptive Banded Event Alignment (ABEA) algorithm (Supplementary Fig. 2A,B) [Gamaarachchi et al. (2020) *BMC Bioinformatics*, 21:343]. These plots show representative distributions of mean current levels for biotinylated and unbiotinylated reads along the same DNA sequence, demonstrating characteristic perturbations induced by biotin-dUTP incorporation. This analysis provides direct evidence that biotinylated and unbiotinylated DNA fragments exhibit distinguishable nanopore signal features, supporting the feasibility of the classification.

To clarify this point, we added the Supplementary Figure 2A & 2B and the sentence, 'To visualize how incorporation of biotin-dUTP perturbs nanopore current signals, we aligned raw current traces to the reference DNA sequence using the Adaptive Banded Event Alignment (ABEA) algorithm (Supplementary Fig. 2A & 2B). This analysis revealed that biotinylated nucleotides exhibit characteristic current patterns that are distinct from those of the four canonical

nucleotides, consistent with previous reports. These results support the feasibility of classifying biotinylated DNA based on nanopore signal features.' to the Result section.

Comment 5: The model was trained on PCR-amplified biotinylated DNA, how similar is this dataset to biotinylated DNA from reconstituted chromatin replication? A direct comparison of signal features between these datasets would be needed. Were both GC-rich and AT-rich sequences included in the training data? If PCR-amplified DNA is AT-rich, the classifier may perform poorly on GC-enriched regions in chromatinized DNA. The 98% accuracy is impressive, but potential sequence-dependent biases (e.g., GC-rich regions) are not explored.

Response 5: We appreciate the reviewer's question regarding the similarity between the training dataset and the biotinylated DNA obtained from reconstituted chromatin replication. In our study, the biotinylated DNA fragments used for training were generated by PCR using the same DNA sequence as the plasmid substrate employed in the chromatin replication assays. As a result, both GC-rich and AT-rich regions present in the experimental substrate are inherently included in the training data. In addition, all proteins were removed prior to library preparation in both cases. Therefore, the nanopore signal features relevant for classification arise from biotin-dUTP incorporation into the same underlying DNA sequence, rather than from differences between PCR-derived and replication-derived DNA.

To clarify this points, we added a sentence, 'Because the same DNA sequence was used for both the replication assays and classifier training, and all proteins were removed prior to library preparation, the classification relies solely on biotin-dUTP incorporation into DNA fragments' to the Result section.

Comment 6: How was the biotin-dUTP concentration determined? Could higher biotin concentrations artificially inflate the fraction of detected biotinylated reads (e.g., from 11%)?

Response 6: For training the classifier, we generated biotinylated DNA fragments by PCR using 50% biotin-dUTP to ensure robust representation of biotin-dependent signal features. In contrast, for Repli-pore-seq measurements, we used 40% biotin-dUTP, which was empirically determined to be the highest concentration that does not interfere with efficient DNA replication, and is therefore lower than

that used for training. Importantly, the false-positive rate of the classifier—estimated from unbiotinylated control reactions—was approximately 0.5% and should be constant regardless of the biotin-dUTP concentration. Consequently, increasing the fraction of biotin-dUTP in the replication reaction would not artificially inflate the fraction of detected biotinylated reads, as unbiotinylated DNA fragments are not misclassified in a concentration-dependent manner. These considerations rule out the possibility that the observed fraction of biotinylated reads arises from an artifact of biotin-dUTP concentration.

To clarify this points, we added a sentence, 'Importantly, the ~0.5% of reads classified as biotinylated in the Mcm10− reaction represent a false-positive background arising from misclassification of unbiotinylated DNA fragments. The false-positive rate is therefore estimated to be approximately ~0.5% and is independent of biotin-dUTP incorporation during replication.' to the Result section.

Comment 7: No loss curve is provided to assess overfitting during training.

Response 7: We appreciate the reviewer's request to assess potential overfitting during classifier training. To address this point, we added a loss curve to Supplementary Fig. 2G, showing the training and validation (cross-validation) losses throughout the training process. Both losses gradually decrease and converge to low values without divergence, indicating that the model does not overfit the training data.

To clarify this point, we modified a sentence in the Result section to 'We trained the classifier to predict whether each read contains biotin-dUTP–incorporated nucleotides, as illustrated by the training and validation loss curves in Supplementary Fig. 2G'.

Comment 8: Assessment of the impact of biotinylation on basecaller performance (e.g., Dorado) is needed.

Response 8: We assessed the impact of biotinylation on basecaller performance by comparing basecalling quality scores (Q-scores) between biotinylated and unbiotinylated DNA fragments. As shown in Supplementary Fig. 2C, biotin-dUTP incorporation reduced basecalling quality relative to unbiotinylated DNA. Importantly, however, the fraction of reads successfully aligned to the reference sequence was comparable for biotinylated and unbiotinylated DNA (93.3% and

91.2%, respectively), indicating that biotinylation does not substantially impair read mapping.

To clarify this point, we added a sentence, 'Although biotin-dUTP incorporation modulated nanopore current signals and reduced basecalling quality scores (Q-scores) (Supplementary Fig. 2C), the fraction of reads successfully aligned to the reference sequence was comparable for unbiotinylated and biotinylated DNA (93.3% and 91.2%, respectively).' to the Result section.

Comment 9: What is the unit of the 600-segment feature vector? If segments correspond to base pairs (bp), why was 600 chosen instead of 400, given that Figure 4B shows read lengths in the 50–500 bp range? Figures 3B and 4B suggest a 50 bp cutoff for read filtering. Was this a hard threshold, and if so, what is its impact on the analysis?

Response 9: We thank the reviewer for requesting clarification regarding the unit and choice of the 600-segment feature vector, as well as the read-length filtering criteria. Each segment in the feature vector corresponds approximately to a k-mer–resolved nanopore current event, such that the number of segments scales roughly with base-pair length rather than representing a one-to-one correspondence with individual base pairs. We selected a maximum length of 600 segments to ensure that the feature representation fully captures nucleosome-sized DNA fragments (~150 bp), including sufficient contextual signal information surrounding each fragment. Using a shorter cutoff (e.g., 400 segments) did not alter classification performance but provided less margin for accommodating variability in signal segmentation.

Regarding read-length filtering, DNA fragments shorter than 40 bp were excluded during library preparation, as such fragments cannot be efficiently recovered using the magnetic bead–based protocol supplied by the manufacturer. This constitutes a hard lower bound imposed by the sequencing workflow rather than an analytical choice. Accordingly, we analyzed reads in the range of 40–400 bp, which encompasses tetrasome-, hexasome-, and nucleosome-sized DNA fragments.

To clarify these points, we added a sentence, "Because DNA fragments shorter than 40 bp are not efficiently recovered during library preparation using the magnetic bead–based protocol, reads shorter than this length were excluded,

and we therefore analyzed DNA fragments in the range of 40–400 bp." to the Result section.

Comment 10: Figure 1B and the third panel of Supplementary Figure 1B are same figures.

Response 10: We thank Reviewer 2 for the careful inspection. We performed three independent replicate experiments, which are shown in Fig. 1B and the first and second panels of Supplementary Fig. 1B. The third panel of Supplementary Fig. 1B was inadvertently duplicated from Fig. 1B. This duplicate panel has been removed in the revised manuscript.

Comment 11: In Supplementary Figure 1B, why is the histone band in lane 7 weaker than in lane 5? If histones bind to DNA, the histone band in the eluate (+DNA, lane 5) should be weaker than in the eluate (−DNA, lane 7) due to reduced free histone availability. Could the authors explain this result?

Response 11: We thank Reviewer 2 for this careful observation and apologize for the insufficient explanation in the original manuscript. Supplementary Fig. 1A was designed to assess the extent of free histone contamination in the eluate after buffer exchange using the S-400 spin column, rather than to compare histone binding efficiency per se. In this experiment, Nap1, ISW1a, and histones were mixed either in the presence (+DNA) or absence (−DNA) of plasmid DNA prior to column filtration. In the absence of DNA (lane 7), histones cannot assemble into nucleosomes and therefore represent free histones that may remain in solution after filtration. The faint histone band in lane 7 indicates that free histones are largely removed by the column. In contrast, in the presence of DNA (lane 5), histones are predominantly incorporated into nucleosomes and co-elute with DNA through the column. As a result, the histone signal in lane 5 reflects histones bound to DNA rather than free histones, explaining why the band intensity is higher than in lane 7.

We have revised the text to explicitly clarify this experimental design and interpretation in the Results section: 'After incubation of the 4.1-kbp plasmid DNA substrate with histones, Nap1, and ISW1a, chromatinized DNA was recovered by gel-filtration (Fig. 1A and Supplementary Fig. 1A, lane 5). In contrast, when an identical mixture lacking DNA was subjected to the same gel-filtration procedure, histones were scarcely detected in the eluate

(Supplementary Fig. 1A, lane 7). These results indicate that the gel-filtration step efficiently removes free histones, while histones incorporated into DNA-bound nucleosomes are retained and recovered with the DNA.'

Comment 12: In Supplementary Figure 1C, why is the band signal in lane 7 stronger than in lane 2? Furthermore, why are the band signals in lanes 7–9 for the DNA:histone = 1:0 condition stronger than other lanes for DNA:histone = 1:1-1:2 if the histone ratio is 0?

Response 12: We thank Reviewer 2 for pointing out the ambiguity in the presentation of Supplementary Fig. 1C and apologize for the insufficient explanation in the original manuscript.

Supplementary Fig. 1C was designed to evaluate whether chromatin assembled by salt-gradient dialysis (SGD) is suitable for *in vitro* chromatin replication. The bands represent DNA replication products before MNase digestion. Under these conditions, stronger bands reflect more efficient DNA replication. The weaker signals observed in lanes 1–6 compared with lane 7 indicate that nucleosome assembly by SGD inhibits DNA replication in a histone-density–dependent manner. In particular, lane 7 corresponds to naked DNA, which supports more efficient replication than chromatinized templates, explaining why its signal is stronger than that in lane 2.

To clarify these points, we added a sentence, "To assess whether chromatin assembled by SGD or by NI provides a suitable substrate for *in vitro* chromatin replication, we assembled nucleosomes on the plasmid DNA using SGD and analyzed the resulting replication products (Supplementary Fig. 1C). Replication proceeded on SGD-assembled chromatin when histones were mixed with plasmid DNA at an equimolar ratio, although nucleosome assembly reduced overall replication efficiency compared with naked DNA (Supplementary Fig. 1C, lanes 1, 2, and 7). Furthermore, little or no signal from the replicated DNA strand was detected when the histone amount was increased to twice that of the plasmid DNA (Supplementary Fig. 1C, lanes 3–6). In contrast, replication reactions on NI-chromatin successfully produced both leading and lagging strands, despite being reconstituted with histones at a 100-fold excess over DNA." to the Result section.

Lanes 8–9 were originally included to examine the effect of excess free histones on DNA replication in the absence of nucleosome assembly. Because

these conditions were not used in subsequent experiments and could potentially lead to confusion, we have removed lanes 8–9 from the revised figure.

Comment 13: Fig. 1E-F. The authors wrote: "Inspection of replicated (biotinylated) DNA after the MNase digestion confirmed nucleosome deposition after replication in the absence of Ctf4, Csm3-Tof1, or Pol δ, in the same way as their presence (Fig. 1E, lanes 2, 4, and 6 & 1F, lanes 2, 4, 6, 8, and 10). These results indicated that Pol δ, Ctf4, and Csm3/Tof1 are dispensable for histone deposition to replicated DNA". This conclusion contradicted the results shown. The authors should quantify replicated DNA in the presence and absence of each factor before Mnase digestion. The authors then need to quantify replicated after Mnase digestion.

Response 13: We thank Reviewer 2 for pointing out the ambiguity in the description of Fig. 1D–H (Fig. 1E–F in the original manuscript) and apologize for the lack of clarity in the original manuscript. As the reviewer correctly pointed out, in Fig. 1D–H, we analyzed MNase-digested DNA both before and after replication under conditions with or without the indicated factors, and compared these datasets to assess whether nucleosome-protected DNA fragments were retained on replicated DNA in each condition. For example, in the bottom panel of Fig. 1D, lanes 1 and 3 analyze the amount of MNase-protected DNA before replication in the absence and presence of Pol δ, respectively. Under both conditions, little to no protected band is detected, indicating that nucleosome-protected fragments are not present prior to replication regardless of Pol δ. In contrast, lanes 2 and 4 analyze the MNase-protected DNA after replication in the absence and presence of Pol δ, respectively, and clear protected bands are observed in both cases. These results demonstrate that nucleosomes are assembled on replicated DNA irrespective of the presence of Pol δ. Therefore, we concluded that Pol δ is dispensable for histone diposition on daughter strands.

To clarify this point, we added sentences 'Inspection of MNase-digested replicated (biotinylated) DNA revealed clear nucleosome-protected fragments after replication both in the presence and absence of Pol δ, Ctf4, Csm3/Tof1, Mrc1, Pol32, or Dpb3/4 (Fig. 1D–H). As a representative example, analysis of the bottom panel of Fig. 1D shows that clear MNase-protected bands are detected on replicated DNA both in the presence and absence of Pol δ (lanes 2 and 4), whereas little to no protected signal is observed prior to replication under either

condition (lanes 1 and 3). This indicates that nucleosomes are assembled on daughter DNA regardless of the presence of Pol δ. Consistent with this observation, MNase-protected fragments were similarly detected after replication in the absence of other replisome components, including Ctf4, Csm3/Tof1, Mrc1, Pol32, and Dpb3/4, at levels comparable to their presence (Fig. 1E–H), demonstrating that these factors are dispensable for nucleosome deposition on replicated DNA.'.

Comment 14: Fig. 4. The authors wrote: "we observed the discordance of the recycled position between leading and lagging strands on the GC-rich DNA sequences". This statement is misleading. As the authors noted that the discordance may be because of GC rich DNA sequences on DNA synthesis, not necessarily on recycled histone positions.

Response 14: We agree with Reviewer 2 that the original wording could be misleading, as it may be interpreted as implying a direct effect of GC-rich DNA sequences on recycled histone positions themselves. Our intention was instead to convey that the observed positional discordance reflects differences in DNA synthesis dynamics on GC-rich regions, which can indirectly influence nucleosome positioning on replicated DNA by altering the relative timing and coordination of lagging- and leading-strand synthesis.

To clarify this point, we modified the sentence to 'Together, we propose that the discordant positioning of nucleosomes on replicated strands reflects uncoupled kinetics of DNA synthesis between the lagging and leading strands on GC-rich DNA sequences, which can indirectly influence nucleosome positioning on daughter DNA.'

Comment 15: As authors stated, some nucleosomes lost one or two H2A/H2B dimers to form hexasomes or tetrasomes after histone recycling. Could the authors perform other experiments to further confirm this conclusion?

Response 15: We agree that direct confirmation of the oligomeric states of recycled histones would require stoichiometric analyses of histone composition on replicated DNA. However, it is technically challenging because the overall efficiency of histone recycling in the *in vitro* reconstitution is low, and immunoprecipitation-based purification of recycled histones did not yield sufficient material, as described in

the Results section.

To clarify this point, we added a sentence "Note that, although our computational approach enables recovery of MNase-digested DNA fragments from low-efficiency replication reactions, direct stoichiometric analysis of recycled histones remains technically challenging because the overall efficiency of histone recycling in the *in vitro* reconstitution is low. Therefore, future studies will require the development of methods that allow efficient isolation of histones specifically associated with replicated DNA to confirm it." to the Discussion section.

Comment 16: In Figure 3B, why do the parental DNA fragments exhibit two peaks? Does this indicate that the recycled histones on parental DNA consist of a mix of mono- and di-nucleosomes?

Response 16: We thank Reviewer 2 for this question. The two peaks observed in the read-length distribution of parental DNA fragments reflect the chromatin organization of the DNA substrate before replication. Specifically, the first and second peaks correspond to mono-nucleosome– and di-nucleosome–protected DNA fragments, respectively. The mean read length of the second peak (275 ± 31 bp) is approximately twice that of a single nucleosome footprint (144 ± 19 bp), indicating the presence of di-nucleosome fragments generated by MNase digestion.

To clarify this point, we modified a sentence in the Result section to 'We observed two distinct peaks in the read-length distribution of parental DNA fragments, corresponding to mono-nucleosome– (144 ± 19 bp) and di-nucleosome–protected fragments (275 ± 31 bp), indicating that nucleosomes are well packed on the DNA substrate prior to replication (Fig. 3B and Supplementary Fig. 3A).'

Comment 17: Since The authors mentioned eSPAN multiple times, the following papers should be cited PMCID: PMC4362665, PMCID: PMC7449674. Since the authors cited Reference Mrc1's role in parental histone recycling, the following reference should be cited. PMCID: PMC11414769

Response 17: We thank the reviewer for pointing out these important references. We have now cited these papers to appropriately reflect prior work.

Responses to Reviewer #3

Comment 1: The manuscript, "Minimal protein list for chromatin replication-coupled symmetric histone recycling revealed through in vitro reconstitution," by Nagae and colleagues, explores the mechanism of histone recycling during chromatin replication using an elegant and sophisticated in vitro system. Additionally, the authors introduce a new bioinformatic pipeline that employs deep learning and nanopore sequencing to identify biotinylated DNA, which may become a valuable tool in the field. Altogether, this study addresses an important question in the field - identifying the replisome components necessary for promoting histone recycling symmetry, and accuracy during DNA replication. The manuscript provides some valuable insights into the roles of Polymerase delta and Ctf4/Tof1 in recycling symmetry and accuracy, respectively, and offers initial evidence of how DNA sequence may influence histone recycling accuracy. However, the benchmarking the accuracy and sensitivity of this in vitro approach against available in vivo data/mutants remains largely limited. To address this and underscore the applicability of the in vitro replication assay, the authors should include additional controls to better estimate the degree of asymmetry detectable in vitro. Moreover, while the system developed is powerful, the study lacks some depth in addressing biological questions. Therefore, the authors should evaluate the impact of more recently discovered histone-binding mutants (potentially in combination, e.g. a leading and lagging strand recycling mutant) and/or test replication stress-inducing drugs on chromatin replication symmetry and accuracy before the manuscript can be considered for publication.

Response 1: We sincerely thank Reviewer 3 for the careful evaluation of our manuscript and for recognizing both the experimental system and the computational framework introduced in this study. We appreciate the constructive comments regarding benchmarking the accuracy of our *in vitro* assay, as well as the suggestion to deepen the analysis by examining additional histone-binding mutants.

As pointed out by the Reviewer 3, the original manuscript lacked a statistical evaluation of histone recycling symmetry. To address this issue, we performed chi-square tests to assess strand-specific recycling frequencies under each experimental condition and evaluated effect sizes using Cohen's *d*. These analyses allowed us to quantify the degree of asymmetry and to benchmark it against known genetic perturbations.

Specifically, omission of Csm3/Tof1 and Ctf4 significantly increased recycling to the leading strand ($P = 2.8 \times 10^{-3}$ and 7.4×10^{-10}, respectively). However, the small effect sizes (Cohen's d = 0.017 and 0.074) indicate that the contributions of these components are modest, consistent with redundancy among histone recycling pathways. In contrast, removal of Pol δ and Pol32− (a histone-binding–defective Pol δ mutant) led to strong leading-strand bias ($P < 2.2 \times 10^{-308}$ and 6.6×10^{-137}), whereas omission of Mrc1 and Dpb3/4− (a histone-binding-defective Pol ε mutant) resulted in significant lagging-strand bias ($P = 8.0 \times 10^{-27}$ and 4.4×10^{-14}). These trends are in excellent agreement with previously reported *in vivo* phenotypes.

In addition, as suggested by the Reviewer 3, we expanded our analysis to include histone-binding mutants of the replisome (the strand-bias effects already described in the preceding paragraph), which necessitated the establishment of new expression strains and purification of the corresponding mutant protein complexes. We performed repli-pore-seq analyses in Pol32− and Dpb3/4− conditions. These results demonstrate that histone binding by Pol δ and Pol ε directly regulates strand-specific histone recycling, further supporting conclusions from earlier *in vivo* studies. Moreover, analysis of the Fen1/Cdc9+ condition revealed increased lagging-strand recycling ($P = 3.7 \times 10^{-36}$), suggesting that lagging-strand maturation contributes to histone recycling.

To clarify these points, we added the following paragraph to the Results section:

'Next, we investigated the effects of Pol δ, Ctf4, Csm3/Tof1, Mrc1−, Pol32−, and Dpb3/4− on the recycling symmetry. Of the genuinely biotinylated DNA fragments in two replicated measurements, 62.9%, 55.0%, 53.8%, 51.1%, 57.8%, and 51.5% were the leading strand fragments in the Pol δ−, Ctf4−, Csm3/Tof1−, Mrc1−, Pol32−, and Dpb3/4− reactions, respectively (Fig. 5A). We observed the statistically significant leading-strand bias in Pol δ−, Ctf4−, Csm3/Tof1−, and Pol32− ($p < 0.01$, Cohen's d = 7.2, $7.4 \times 10{-2}$, $1.7 \times 10{-2}$, and 1.1; chi-square test) and lagging-strand bias in Mrc1− and Dpb3/4− ($p < 0.01$, Cohen's d = 0.20, and 0.10; chi-square test) (Fig. 5A). The observed bias was consistent with previous results of *in vivo* experiments using ctf4Δ20, mrc1Δ25, pol32Δ21,22, and dpb3Δ24 mutants of Saccharomyces cerevisiae. Despite a different type of mutation, the leading-strand bias in Csm3/Tof1− was consistent with tof1-3A, which has mutations in residues interacting with the N-terminal tail of Mcm2. Furthermore, Pol δ−, in which lagging-strand synthesis was compromised

showed more intense lagging-strand bias than Pol32−, suggesting that a balance between the lagging and leading strand synthesis was also required for symmetric histone recycling.'

Overall, these additional statistical analyses and mutant validations demonstrate that our *in vitro* reconstitution system quantitatively recapitulates known *in vivo* behaviors and provides sufficient sensitivity to detect asymmetries in histone recycling. We thank Reviewer 3 for prompting these analyses, which have significantly strengthened the rigor of our conclusions.

Comment 2: The study focuses on Polymerase delta's role in lagging strand recycling, but additional controls are necessary. The authors should introduce mutations in Mcm2, or omit Polymerase epsilon subunits 3 and 4 (Pole3/4, or Dpb3/4 in yeast) as well as Polymerase delta subunit Pold32 to better recapitulate the in vivo effects in their in vitro system. Ideally, combinations of these mutants (disabling leading and lagging strand recycling) would also reveal to what extent histones may be lost during DNA replication and to what an "intrinsic" affinity of parental histones to newly synthesized DNA may compensate for disabled histone recycling.

Response 2: We thank Reviewer 3 for the suggestions regarding additional controls to better benchmark our *in vitro* system against *in vivo* histone recycling pathways. As described in Response 1, we performed Repli-pore-seq analyses using Pol32− and Dpb3/4− mutants (generation of a expression strain of Mcm2 mutant was not successful due to technical limitations) and observed strand-specific recycling biases that are consistent with previously reported *in vivo* phenotypes.

Regarding the Reviewer's suggestion to assess the "intrinsic" affinity of parental histones for newly synthesized DNA, we note that quantification of such an effect would require disabling multiple histone recycling pathways simultaneously. This would involve omission or mutation of components that are essential for efficient chromatin replication, which in our *in vitro* system reduces nucleosome footprint signals, precluding reliable evaluation. Nevertheless, we agree that combinatorial depletion or mutation of histone recycling factors represents an important future direction.

To clarify this point, we added a sentence 'Previous studies have identified multiple proteins—including Mcm2, Pol α, Ctf4, Mrc1, Tof1, Pol32 (Pol δ), FACT, PCNA, and Dpb3/4 (Polα)—that cooperatively ensure symmetric histone

recycling during DNA replication[17,19–30]. In the present study, the Repli-pore-seq analysis successfully recapitulated the strand-specific phenotypes reported *in vivo* using mutants of these replication factors. Moreover, recent work has proposed extensive functional crosstalk among these proteins in coordinating histone recycling[20–23,25–30]. Future comprehensive analyses using combinatorial mutants will be essential to fully elucidate the interaction network governing histone recycling during chromatin replication.' to the Discussion section.

Comment 3: A third biological replicate for histone recycling accuracy and positioning on the leading vs. lagging strand would significantly enhance the statistical robustness of the study.

Response 3: We thank Reviewer 3 for the suggestion to include an additional biological replicate to strengthen the statistical robustness of our analysis. In response, we performed a third independent biological replicate of the repli-pore-seq analysis using the minimal reconstitution system. Across three biological replicates, the fraction of histones recycled to the leading strand was 52.5 ± 0.7%, indicating highly symmetric recycling between the leading and lagging strands. In addition, pairwise comparisons of histone positioning between replicates showed strong correlations ($R = 0.99$ for replicate 1 vs. 2, $R = 0.97$ for replicate 2 vs. 3, and $R = 0.97$ for replicate 3 vs. 1), demonstrating high reproducibility of both recycling accuracy and positional distributions.

To clarify this point, we added sentences 'Interestingly, 52.5 ± 0.7% of the daughter DNA fragments were classified as leading-strand fragments across three independent experiments (Fig. 4D), indicating that histones are recycled almost symmetrically between the lagging and leading strands with slight leading strand bias in our *in vitro* reconstitution system, consistent with observations *in vivo*. Based on the correction for misclassified reads (see Methods for details), we estimated that the fraction of reads misclassified as leading-strand fragments was approximately 0.2%. This value is far smaller than the strand-specific recycling biases observed in our experiments (on the order of several percent) and also smaller than the standard deviation across three independent measurements (0.7%), indicating that any distortion of the recycling ratio due to misclassification is negligible. Pairwise correlations of positional distributions among the three replicates were high ($R = 0.97$–0.99; Supplementary Fig. 4A), whereas the correlation between lagging- and leading-

strand fragments was moderate (R = 0.52; Fig. 4E).' to the Result section.

Moreover, motivated by the reviewer's emphasis on statistical rigor, we extended our analyses to systematically re-evaluate strand-specific recycling biases under all conditions using formal statistical testing. Through this more rigorous analysis, we found that addition of the lagging-strand maturation factors Fen1/Cdc9 exerts a statistically significant effect on strand bias, specifically enhancing histone recycling to the lagging strand. Accordingly, we revised the Results and Discussion throughout the manuscript to incorporate this finding. We again thank Reviewer 3 for this constructive suggestion.

Comment 4: The NI-system is complex and requires further clarification, in particular regarding the de novo H2A-H2B (and H3-H4) deposition activity by Nap1 on replicated chromatin. The authors should exclude the possibility that Nap1 deposits histones independently of DNA replication?

Response 4: We agree with Reviewer 3 that the contribution of Nap1 to de novo histone deposition requires careful clarification. To address this concern, we examined whether Nap1 could deposit free histones independently of DNA replication. Nap1, ISW1a, and histones were mixed at the same concentrations used for nucleosome assembly but in the absence of naked DNA substrates, and the mixture was subjected to gel filtration following the same procedure as in Supplementary Fig. 1A to recover contaminated proteins. Because DNA was omitted in this control preparation, the resulting fraction is expected to contain higher levels of free Nap1, ISW1a, and histones than in the nucleosome assembly reaction. Using this solution, we performed replication reactions with naked DNA substrates and compared the results with those obtained from chromatin replication. In reaction A, the ~150 bp biotinylated DNA band was barely detectable, whereas a clear band was observed in the chromatin replication reaction (Supplementary Fig. 1E and 1F, lanes 3 and 6). These results indicate that DNA replication-independent nucleosome assembly mediated by Nap1 and ISW1a is negligibl

To clarity this points, we added a sentence 'Furthermore, to examine whether Nap1 can mediate replication-independent histone deposition, we prepared a control fraction by mixing Nap1, ISW1a, and histones at the same concentrations used for nucleosome assembly but without naked DNA substrates, followed by gel filtration. Replication reactions performed with naked DNA substrates in this

fraction produced only a barely detectable ~150 bp biotinylated DNA band (Supplementary Fig. 1E and 1F, lanes 3 and 6), indicating that Nap1- and ISW1a-mediated nucleosome assembly in the absence of chromatin replication is negligible.'

Comment 5: A 20× discrimination between true and false positives may not be sufficient to resolve the subtle differences observed in Polymerase delta mutants, especially considering potential DNA synthesis reduction, which could further skew the ratio of true to false positives.

Response 5: We agree with Reviewer 3 that misclassification of biotinylated reads could, in principle, distort the estimated recycling ratio between the lagging and leading strands.

To address this concern, we quantified the false-positive rate using Replipore-seq data obtained from the Mcm10− reaction, in which no genuine biotin incorporation is expected. Based on this dataset, we estimated the fraction of genuinely biotinylated reads and corrected the strand-specific recycling ratios according to the procedure described in the "Correction for misclassified reads" section of the Methods. Importantly, after applying this correction, the fraction of reads misclassified as leading-strand fragments was estimated to be approximately 0.2%, which is far smaller than the strand-specific recycling biases observed in our experiments (e.g., ~10% in Pol δ− and ~6% in the Pol32− mutant), and also substantially smaller than the standard deviation across three independent biological replicates (~0.7%). This indicates that potential distortion of the recycling ratio due to misclassification is negligible relative to experimental variability.

To clarify this point, we added a sentence 'Based on the correction for misclassified reads (see Methods for details), we estimated that the fraction of reads misclassified as leading-strand fragments was approximately 0.2%. This value is far smaller than the strand-specific recycling biases observed in our experiments (on the order of several percent) and also smaller than the standard deviation across three independent measurements (0.7%), indicating that any distortion of the recycling ratio due to misclassification is negligible.' to the Result section.

Comment 6: Given that H2A-H2B are proposed to be also recycled, does the tetramer/hexamer ratio change if Nap1 is omitted from the reaction? If so, this would suggest that Nap1 deposits H2A-H2B independently to form hexamers/octamers on replicated chromatin rather than through histone recycling.

Response 6: We agree that examining how the tetramer/hexamer ratio changes upon modulation or omission of Nap1 is an intriguing and important question, as it could provide direct insight into whether Nap1 contributes to H2A–H2B deposition independently of histone recycling. At present, however, addressing this question is technically challenging. First, control of Nap1 concentration after chromatin reconstitution is difficult because residual Nap1 cannot be quantitatively tuned using the current spin-column–based procedure. Second, distinguishing between tetrasomes and hexasomes based solely on the length of MNase-protected DNA fragments is inherently challenging. For these reasons, we are not able to assess changes in the tetramer/hexamer ratio upon Nap1 modulation. We therefore consider this analysis an important goal for future work. We appreciate the reviewer for highlighting this promising direction.

To clarify these points, we added a sentences "Furthermore, in our experimental system, Nap1 and ISW1a, which are required for nucleosome reconstitution, could not be completely removed after the gel-filtration step. Nap1 is known to dismantle H2A–H2B dimers from partially unwrapped nucleosomes and to deposit H2A–H2B dimers onto tetrasomes. Consequently, residual Nap1 may contribute to H2A–H2B recycling and thereby modulate the relative proportions of tetrasomes, hexasomes, and nucleosomes formed on replicated DNA. Future Repli-pore-seq analyses with improved control of Nap1 carryover would provide further insight into the molecular mechanisms of H2A–H2B recycling, which underlie short-term memory in the restoration of chromatin epigenetic states." to the Discussion section.

Comment 7: Please provide a full list of proteins used in the study, especially listing the subunits of the Polymerases used.

Response 7: We have provided a supporting table and now explicitly refer to it in the revised manuscript, as stated in the sentence: 'In this study, we identified 24 purified

proteins as the minimal set required to reconstitute symmetric histone recycling using *in vitro* chromatin replication and the newly developed Repli-pore-seq method (Fig. 7 and Supplementary Table 1).'

Comment 8: Clarify the meaning of LSTM abbreviation.

Response 8: LSTM stands for Long Short-Term Memory, and the full term is spelled out at its first mention in the manuscript.

Comment 9: The axis label of Figure 4 is a bit confusing. How can probability vary between 0 to 1 (Figure 4D) and be negative or slightly positive (remaining panels in Figure 4)? Do the authors refer to the relative probabilities vs absolute probability? Please elaborate.

Response 9: We apologize for the confusion caused by the axis labels in Fig. 4 (More specifically Fig. 4C & 4F). In these figure pannels, we analyzed strand-specific recycling by calculating probability distributions of recycled histone positions separately for the lagging and leading strands, where frequencies were normalized by the total number of events on each strand. In the previous version, probabilities of histone recycling to the leading strand were displayed above zero, whereas probabilities of recycling to the lagging strand were displayed below zero. In this representation, values appear positive or negative solely to visually separate the two strand-specific distributions and do not indicate negative probabilities. To avoid this confusion, we have revised the figure presentation by separating the plots into two distinct panels for the leading and lagging strands and clarified the corresponding axis labels, accordingly.

Comment 10: To complement histone recycling mutants, the authors could also use replication stress-inducing drugs and assess their effects on histone recycling accuracy in vitro. This would provide valuable insights that may be difficult to visualize in vivo due to pleiotropic effects of these drugs (e.g. checkpoint signaling)

Response 10: We agree with Reviewer 3 that replication stress–inducing drugs represent a promising avenue to further probe histone recycling mechanisms. As suggested, our *in vitro* reconstitution approach is particularly well suited for evaluating the effects of such perturbations, which often produce pleiotropic or lethal effects *in*

vivo and complicate interpretation due to checkpoint activation and other secondary responses.

To emphasize this complementary aspect, we added a sentence '*In vitro* reconstitution studies offer a advantage for investigating drug treatments as well as protein mutations or depletions that can cause pleiotropic or lethal effects in cellular systems.' and 'Taken together, the Repli-pore-seq pipeline expands the scope of sequencing-based analyses to defined *in vitro* reconstitution systems, providing insights that may be difficult to obtain *in vivo* due to the pleiotropic effects of genetic perturbations or drug treatments.' to the Discussion section.

Responses to Reviewer #2

Comment 1: In the revised manuscript, the authors tested the effects of mission of several components (Pol32, Ctf4, Dpb3/Dpb4, Mrc1) known to be involved in parental histone recycling/transfer. While these results largely strengthened the manuscript, the results shown largely confirmed what is known about parental histone transfer. Therefore, the authors need to highlight new insights into this process gained from this study. In addition, I have additional concerns and suggestions based on new results presented.

Response 1: We sincerely thank Reviewer 2 for the careful and critical evaluation of our manuscript, again. We agree that the novelty of this study should be more clearly emphasized. In particular, a key advance of the present work is that we successfully reconstituted parental histone recycling using only purified components and directly visualized strand-specific recycling toward the leading and lagging strands, thereby enabling (i) identification of the minimal factors required for symmetric histone recycling, (ii) uncoupling histone recycling from subsequent restoration of histone modifications, and (iii) systematic analysis of factor depletion conditions, some of them are difficult or lethal to examine in cellular systems.

In cellular systems, parental histone recycling is examined in the presence of numerous replication-associated and chromatin-regulatory factors, making it difficult to distinguish direct from indirect contributions to histone transfer. For example, recent proximity proteomics analyses identified hundreds of MCM2- and POLE3/4-associated proteins potentially linked to parental histone inheritance, including multiple additional histone chaperones and replication factors such as POLD3, BRD4, SSRP1, and DNAJC9 [*Cell Death & Differentiation* (2025); DOI: 10.1038/s41418-025-01619-z]. By contrast, our fully reconstituted system demonstrated that symmetric parental histone recycling can be reproduced with a minimal and defined set of purified factors, thereby enabling direct mechanistic dissection of the core recycling machinery.

Moreover, in cellular systems, parental histone recycling is tightly coupled to the restoration of histone modifications through the action of histone-modifying enzymes and chromatin assembly pathways. As a result, it is often difficult to distinguish whether observed epigenetic inheritance phenotypes arise from the recycling of parental histones themselves or from subsequent restoration of

histone modifications on newly deposited histones. In our system, the absence of histone-modifying enzymes allowed us to isolate the recycling process itself and directly evaluate how individual replication factors contribute to strand-specific parental histone transfer.

Furthermore, the reconstituted system enabled us to investigate the functions of replication factors whose contributions are difficult to isolate *in vivo* because of lethality or redundant pathways. For example, by omitting Pol δ from the reaction, we were able to directly evaluate its contribution to parental histone recycling independently of secondary cellular effects. This analysis revealed not only the importance of the histone-binding activity associated with Pol δ subunits, but also the effect of replication-speed decoupling on the symmetry of parental histone transfer. In addition, our Fen1/Cdc9 omission experiments provided mechanistic insight into the relationship between lagging-strand maturation and histone recycling. In cellular systems, analysis of this process is complicated by the presence of the Dna2-dependent redundant pathway, while complete disruption of both pathways is difficult because Dna2 deletion causes lethality. These points became clear through the revisions based on Reviewer 2's comments, and we deeply appreciate these important suggestions, which substantially improved the manuscript.

To clarify these points, we modified and added sentences "The key advance of the present work is that we successfully reconstituted parental histone recycling using only purified components and directly visualized strand-specific recycling toward the leading and lagging strands, thereby enabling (i) identification of the minimal factors required for symmetric histone recycling, (ii) uncoupling histone recycling from subsequent restoration of histone modifications, and (iii) systematic analysis of factor depletion conditions, some of them (i.e. Pol δ and Fen1/Cdc9) are difficult or lethal to examine in cellular systems." to the Discussion section.

Comment 2: "Moreover, certain PTMs, which have been used as markers of old histones, can be added to newly synthesized histones by histone-modifying enzymes6,10. These enzymes, which alter the epigenetic states of nucleosomes, complicate the interpretation of genome-wide sequencing studies". The statement is confusing. The restoration of modifications on new histones is an integral part of chromatin replication process.

Response 2: We apologize that our original explanation was unclear and did not sufficiently convey the intended point of this statement. As Reviewer 2 correctly pointed out, parental histone recycling and restoration of histone modifications on newly deposited histones are tightly integrated processes during chromatin replication. Our intention was to emphasize that this tight coupling between parental histone recycling and restoration of histone modifications makes it difficult to isolate each protein factor's contribution on histone recycling and thereby complicates mechanistic investigation of the recycling process in cellular systems. Thus, as described in Response 1, one of the key advances of the present study is that our purified reconstituted system allowed us to eliminate the influence of epigenetic modification enzymes and directly examine DNA replication-coupled parental histone recycling itself in isolation from downstream chromatin restoration processes. To clarify these points, we modified the sentences to "Moreover, epigenetic modification enzymes restore histone modification patterns by introducing certain PTMs, including those commonly used as markers of parental histones, onto newly synthesized histones during chromatin replication. Because this restoration process is tightly coupled to parental histone recycling at the replication fork *in vivo*, it is difficult to distinguish the direct contribution of histone recycling itself from downstream chromatin restoration processes and to mechanistically dissect the role of individual replication factors in epigenetic inheritance." in the Introduction sections.

Comment 3: Fig. 1E-H. The relative amount of newly synthesized nucleosomal fragments in reactions lacking Ctf4, Pol32 and Dpb3-Dpb4 should be quantified and see whether each omission affect nucleosome assembly and/or DNA replication.

Response 3: We quantified the signal intensities of newly synthesized nucleosomal fragments in reactions lacking Ctf4, Pol32, and Dpb3/4 and normalized each value to the corresponding reaction containing these factors. The normalized intensity in the Ctf4− reaction was approximately 1.0, indicating that omission of Ctf4 did not substantially affect overall nucleosome assembly. In contrast, the normalized intensities in the Pol32− and Dpb3/4− reactions were approximately 0.6, suggesting that omission of Pol32 or Dpb3/4 moderately reduced nucleosome assembly and/or DNA replication efficiency. Nevertheless, these reactions still produced sufficient levels of nucleosome footprints on newly synthesized DNA

strands for downstream sequencing analysis. These results further support our conclusion that Pol32 and Dpb3/4, similar to Pol δ, Csm3/Tof1, and Mrc1, are not strictly required for parental histone recycling itself, but instead contribute to the efficiency and symmetry of the process. To clarify these points, we modified the sentence to "Although omission of Pol32 and Dpb3/4 moderately decreased the levels of newly synthesized nucleosomal fragments compared with reactions containing these factors (Supplementary Fig. 1G), substantial nucleosome deposition on replicated DNA was still observed, indicating that these factors are not strictly required for parental histone deposition itself." in the Result section.

Comment 4: Fig. 5H-M. Based on these results, the authors proposed that removal of Ctf4, Csm3/Tof1, or Mrc1 shifts recycled histones toward energetically less favorable positions and that these factors coordinate DNA sequence–dependent positioning of recycled histones (Fig. 5H to 5M). These results and conclusion do not make sense. How can proteins involved in DNA replication, which are sequence independent, confer sequence-dependent nucleosome position? How accurate/confident are for the estimation of nucleosome positions using this approach? Since the chromatin is digested with MNase, the authors should use short read sequencing to map nucleosome position as an independent approach to confirm the results/conclusion. Also what is the advantage to use long-read sequencing compared to short-read sequencing to map nucleosome position?

Response 4: We sincerely thank Reviewer 2 for raising this important point and for prompting us to clarify both the reliability of our nucleosome-position analysis and the interpretation of these results. First, we would like to emphasize that we carefully evaluated the reproducibility and robustness of the estimated nucleosome positions before drawing mechanistic conclusions. To assess experimental variability, we calculated the fold changes of normalized nucleosome distributions between all possible combinations of three independent experiments. The resulting $\log_2$FC values were centered around zero, and only ~0–4% and ~0–8% of positions on the lagging and leading strands, respectively, showed values greater than 0.5 (Supplementary Fig. 5M to 5O). These analyses indicate that the estimated nucleosome positions are highly reproducible across independent experiments and that the vast majority of observed positional changes cannot be explained by coincidental fluctuations or experimental noise.

To clarify this point, we added a sentence. "Note that between independent experiments in the presence all the corresponding factors, the $\log_2$FC values were around zero, and 0–4 % and 0–8 % of positions on the lagging and leading strand were greater than 0.5, respectively, indicative of robust and reproducible positioning of nucleosome (Supplementary Fig. 5M to 5O)." to the Result section.

We next sought to clarify the possible mechanism underlying the altered nucleosome-position distributions observed upon omission of specific replication factors. Because parental histone recycling at the replication fork occurs in a highly dynamic non-equilibrium environment, the final nucleosome positions are not determined solely by intrinsic DNA sequence preference, but can also be influenced by the kinetics and timing of histone deposition during DNA replication. In our previous molecular simulation study, we suggested that the initial deposition positions of recycled histones are affected by the local conformational dynamics of DNA and histones around the replication fork [*Nature Communications* (2024) doi: 10.1038/s41467-024-53187-4]. Since Csm3/Tof1, Ctf4, and Mrc1 interact with histones or histone chaperones and participate in parental histone recycling pathways, omission of these factors could alter the kinetics of histone transfer and deposition, thereby shifting the final distribution of recycled histones. To clarify these points, we added the sentences "Furthermore, removal of Mrc1, Csm3/Tof1, or Ctf4 increased the abundance of recycled histones at energetically less favorable positions compared with reactions containing these factors. As shown in Fig. 3F, the recycled positions are largely influenced by DNA sequence, which determines the thermodynamic landscape of DNA bending and nucleosome formation. However, because parental histone recycling at the replication fork occurs in a highly dynamic non-equilibrium environment, the final nucleosome positions can deviate from the ideal sequence-dependent distribution. Our previous molecular simulation study suggested that the initial deposition positions of recycled histones are influenced by the local conformational dynamics of DNA and histones that transiently probe each other around the replication fork. Previous studies[20,25–27,48] also suggested that Csm3/Tof1, Ctf4, and Mrc1 interact with histones or histone chaperones during DNA replication. Therefore, omission of these factors could alter the kinetics and timing of histone transfer and deposition, thereby shifting the final distribution of recycled histones. Future direct visualization of replication-coupled histone recycling dynamics will be

important to test whether the kinetics of histone deposition modulates the final nucleosome positions." to the Discussion section.

We agree that independent validation using short-read sequencing platforms such as Illumina sequencing would be an attractive approach. However, in our experimental system, such validation is technically difficult because conventional short-read sequencing platforms cannot directly detect nucleotide analogues incorporated into newly synthesized DNA and therefore require biochemical purification or enrichment of labeled nascent DNA prior to sequencing. In contrast, nanopore sequencing enables direct detection of nucleotide analogues from individual DNA molecules in the sequencing library, allowing us to bypass biochemical purification steps. Because the amount of newly synthesized DNA obtained from our *in vitro* reconstituted reactions is extremely limited, immunoprecipitation-based enrichment required for short-read sequencing is not practically feasible in the current system. Therefore, nanopore sequencing provides a unique advantage for analyzing replication-coupled histone recycling in our low-input reconstituted assay.

Comment 5: Fig. 6. In vivo study indicate that Fen1 deletion does not affect parental histone transfer bias, which is in consistent with in vitro system. Did the authors perform experiments without Fen1 alone?

Response 5: We thank Reviewer 2 for this important suggestion regarding additional comparison with *in vivo* studies. To our knowledge, however, there has been no previous direct demonstration in the literature that Fen1 deletion does not affect parental histone recycling bias *in vivo*. If any, the absence of a strong phenotype in Fen1-deficient cells would not necessarily indicate that lagging-strand maturation is dispensable for histone recycling, because the Dna2 pathway can function redundantly with Fen1 during Okazaki fragment maturation [*Cold Spring Harb. Perspect. Biol.* (2013) DOI: 10.1101/cshperspect.a010173]. Therefore, omission of Fen1/Cdc9 in our reconstituted system does not directly correspond to a simple Fen1 deletion in cellular systems. Moreover, complete disruption of lagging-strand maturation pathways *in vivo* might be technically challenging because Dna2 deletion causes lethality. In contrast, our purified *in vitro* system bypasses such cellular constraints and enabled us to directly investigate how defects in lagging-strand maturation influence parental histone recycling. We believe this represents a unique advantage of the reconstituted

system and provides new mechanistic insight into the relationship between Okazaki fragment maturation and histone recycling. To clarify these points, we added sentences, "In the current study, to investigate the effect of lagging-strand maturation on parental histone recycling, we performed Repli-pore-seq analysis using reactions lacking Fen1/Cdc9, which constitute a primary pathway for Okazaki fragment maturation. In eukaryotic cells, however, flap structures generated during lagging-strand synthesis are processed not only by Fen1, but also by the Dna2-dependent pathway, which functions redundantly in Okazaki fragment maturation[74]. Therefore, complete evaluation of the contribution of lagging-strand maturation to histone recycling *in vivo* would require simultaneous disruption of these redundant pathways. However, because Dna2 deletion causes lethality[74], comprehensive analysis of lagging-strand maturation defects and their impact on histone recycling is technically challenging in cellular systems. In contrast, the in vitro reconstituted system bypasses such cellular constraints and enabled us to directly demonstrate that perturbation of Okazaki fragment maturation alters parental histone recycling patterns, thereby providing new mechanistic insight into the relationship between lagging-strand maturation and histone recycling." to the Discussion sections.

Responses to Reviewer #3

Comment 1: The authors have provided a substantially improved manuscript and added additional, insightful experiments that address most of this reviewer's comments. They provide further controls using a number of in vivo derived histone recycling mutants and can recapitulate their effects in vitro. Also, they invested more time in clarity, completeness and important details throughout the whole manuscript. One small thing remains from this reviewer's side: It should be still possible to analyse the recycling accuracy and symmetry in double mutants that do not affect DNA synthesis (e.g. the Pold32 and Dpb3/4), as in their in vitro system, one does not need to generate elaborate double mutants but rather just omit complex subunits from their mix (e.g. omit Pold32, Dpb3, Dpb4). Looking at the effect of reduced histone recycling to both daughter strand would bring in additional novelty to this manuscript.

Response 1: We sincerely thank Reviewer 3 for the careful evaluation of our manuscript and for this constructive suggestion regarding analysis of double mutants in the

reconstituted system. As Reviewer 3 pointed out, omission of multiple histone recycling factors could provide important insight into whether distinct recycling pathways function additively, redundantly, or cooperatively during parental histone transfer.

However, an important limitation of the current Repli-pore-seq pipeline is that it primarily measures the relative strand bias of parental histone recycling (i.e., the ratio of recycling toward the leading versus lagging strands), rather than the absolute amount of recycled histones. In the current experimental setup, the total number of recovered reads is strongly influenced by sequencing yield and library recovery efficiency, making it difficult to quantitatively compare absolute histone recycling levels between different reactions. Therefore, while our method can robustly detect shifts in recycling symmetry, it is currently difficult to conclude that omission of multiple factors reduces parental histone recycling equally on both daughter strands.

To quantitatively compare absolute histone recycling efficiencies across samples, it would be necessary to introduce an external spike-in DNA standard and normalize the enrichment of target reads against the spike-in signal, as performed in previous *in vivo* studies [e.g. *Nat. Protoc.* (2021) doi: 10.1038/s41596-021-00585-3]. However, during development of the current Repli-pore-seq workflow, we removed the spike-in normalization step because competition between spike-in DNA and target DNA substantially reduced recovery of biotinylated reads from the low-input in vitro reactions. Consequently, the present version of the Repli-pore-seq method is not yet optimized for rigorous quantification of absolute histone recycling efficiency in double-mutant conditions. Nevertheless, we greatly appreciate Reviewer 3's insightful suggestion, which provides an important direction for future methodological improvement.

To clarify these points, we modified to sentences "In those studies, they quantified the absolute density of recycled histones on replicated DNA in combinatorial mutants and demonstrated additive effects on histone recycling, supporting the coexistence of multiple recycling pathways. In contrast, the current Repli-pore-seq procedure is primarily designed to measure the relative strand bias of parental histone recycling rather than the absolute amount of recycled histones. Because our workflow omits the spike-in DNA normalization step, the total read count is influenced by sequencing yield and library recovery efficiency, making rigorous comparison of absolute histone recycling levels

between samples difficult. Although omission of the spike-in step maximizes recovery of low-abundance biotinylated reads from inefficient in vitro replication reactions, it limits quantitative assessment of global decreases in histone recycling efficiency. Future improvement of the Repli-pore-seq procedure, including robust spike-in normalization, together with comprehensive analyses of combinatorial mutants, will be important for fully elucidating the interaction network governing parental histone recycling during chromatin replication." in the Discussion section.

Responses to Reviewer #2

Comment 1: The following statement is not accurate. "Although omission of Pol32 and Dpb3/4 moderately decreased the levels of newly synthesized nucleosomal fragments compared with reactions containing these factors (Supplementary Fig. 1G), substantial nucleosome deposition on replicated DNA was still observed, indicating that these factors are not strictly required for parental histone deposition itself." Substantial nucleosome deposition on replicated DNA was still observed during omission of Pol32 and Dpb3/Dpb4 do not mean that these proteins are not strictly required for parental histone deposition. Instead, the data indicate that other factors may function in parental histone transfer in their absence.

Response 1: We sincerely thank Reviewer 2 for carefully reviewing our manuscript and offering valuable advice. We agree with the Reviewer's concern that the current statement about effects of Pol32Δ and Dpb3/4Δ might cause misleading and confusion about conclusion of this study. We modified the sentence as "Although substantial nucleosome deposition on replicated DNA was still observed, omission of Pol32 or Dpb3/Dpb4 moderately reduced the levels of newly synthesized nucleosomal fragments compared with reactions containing these factors (Supplementary Fig. 1G). These results suggest that Pol32 and Dpb3/Dpb4 contribute to efficient histone recycling, while other factors may partially support parental histone transfer in their absence." in the Results section.

Comment 2: Fig. 6. In vivo study indicate that Fen1 deletion does not affect parental histone transfer bias, which is in consistent with in vitro system. Did the authors perform experiments without Fen1 alone? The reference describing the effects of Fen1 deletion and DNA2 depletion on parental histone transfer is PMID: 38838138.

Response 2: We greatly appreciate Reviewer 2 for bringing this important reference to our attention. As the Reviewer pointed out, a previous study reported that single deletion of Fen1 or Dna2 did not cause an obvious strand bias in parental histone recycling [Sci. Adv. (2024) DOI: 10.1126/sciadv.adn5175]. However, the Fen1- and Dna2-dependent pathways can function redundantly during Okazaki fragment maturation [Cold Spring Harb. Perspect. Biol. (2013) DOI: 10.1101/cshperspect.a010173]. Therefore, Okazaki fragment maturation is

unlikely to be completely abolished in either single mutant, making these cellular mutant conditions not directly comparable to the Fen1/Cdc9− condition in our reconstituted system. We believe that our *in vitro* reconstitution system, in which this Okazaki fragment maturation module can be omitted, provides new mechanistic insight into the relationship between Okazaki fragment maturation and parental histone recycling. Nevertheless, we agree that additional explanation is necessary to avoid confusion with the previous findings. We have therefore modified the relevant sentence in the Discussion section as follows: "Repli-pore-seq analysis of reactions containing Fen1 and Cdc9, which constitute a major pathway for Okazaki fragment maturation, showed enhanced histone recycling to the lagging strand. In contrast, a previous study reported that deletion of Fen1 in *Saccharomyces cerevisiae* did not alter the strand bias of parental histone recycling, which appears inconsistent with our result. In eukaryotic cells, however, flap structures generated during lagging-strand synthesis are processed not only by Fen1 but also by the Dna2-dependent pathway, which functions redundantly in Okazaki fragment maturation. Thus, a single deletion of Fen1 or Dna2 in cells is not directly comparable to the Fen1/Cdc9− condition in our reconstituted system, which lacks this Okazaki fragment maturation module."
